# The photosynthetic bacteria *Rhodobacter capsulatus* and *Synechocystis* sp. PCC 6803 as new hosts for cyclic plant triterpene biosynthesis

**DOI:** 10.1371/journal.pone.0189816

**Published:** 2017-12-27

**Authors:** Anita Loeschcke, Dennis Dienst, Vera Wewer, Jennifer Hage-Hülsmann, Maximilian Dietsch, Sarah Kranz-Finger, Vanessa Hüren, Sabine Metzger, Vlada B. Urlacher, Tamara Gigolashvili, Stanislav Kopriva, Ilka M. Axmann, Thomas Drepper, Karl-Erich Jaeger

**Affiliations:** 1 Institute of Molecular Enzyme Technology, Heinrich Heine University Düsseldorf, Forschungszentrum Jülich, Jülich, Germany; 2 Cluster of Excellence on Plant Sciences (CEPLAS); 3 Institute for Synthetic Microbiology, Department of Biology, Heinrich Heine University Düsseldorf, Düsseldorf, Germany; 4 MS Platform, Department of Biology, University of Cologne, Cologne, Germany; 5 Institute of Biochemistry II, Department of Chemistry, Heinrich Heine University Düsseldorf, Düsseldorf, Germany; 6 Botanical Institute, University of Cologne, Cologne, Germany; 7 Institute of Bio- and Geosciences (IBG-1), Forschungszentrum Jülich, Jülich, Germany; Simon Fraser University, CANADA

## Abstract

Cyclic triterpenes constitute one of the most diverse groups of plant natural products. Besides the intriguing biochemistry of their biosynthetic pathways, plant triterpenes exhibit versatile bioactivities, including antimicrobial effects against plant and human pathogens. While prokaryotes have been extensively used for the heterologous production of other classes of terpenes, the synthesis of cyclic triterpenes, which inherently includes the two-step catalytic formation of the universal linear precursor 2,3-oxidosqualene, is still a major challenge. We thus explored the suitability of the metabolically versatile photosynthetic α-proteobacterium *Rhodobacter capsulatus* SB1003 and cyanobacterium *Synechocystis* sp. PCC 6803 as alternative hosts for biosynthesis of cyclic plant triterpenes. Therefore, 2,3-oxidosqualene production was implemented and subsequently combined with different cyclization reactions catalyzed by the representative oxidosqualene cyclases CAS1 (cycloartenol synthase), LUP1 (lupeol synthase), THAS1 (thalianol synthase) and MRN1 (marneral synthase) derived from model plant *Arabidopsis thaliana*. While successful accumulation of 2,3-oxidosqualene could be detected by LC-MS analysis in both hosts, cyclase expression resulted in differential production profiles. CAS1 catalyzed conversion to only cycloartenol, but expression of LUP1 yielded lupeol and a triterpenoid matching an oxidation product of lupeol, in both hosts. In contrast, THAS1 expression did not lead to cyclic product formation in either host, whereas MRN1-dependent production of marnerol and hydroxymarnerol was observed in *Synechocystis* but not in *R*. *capsulatus*. Our findings thus indicate that 2,3-oxidosqualene cyclization in heterologous phototrophic bacteria is basically feasible but efficient conversion depends on both the respective cyclase enzyme and individual host properties. Therefore, photosynthetic α-proteo- and cyanobacteria are promising alternative candidates for providing new bacterial access to the broad class of triterpenes for biotechnological applications.

## Introduction

Plant secondary metabolites comprise a large variety of structurally divergent compounds that can serve as signaling molecules or as protecting agents against microbial pathogens, herbivorous attacks or plant competitors (overview in [1]). Among these secondary metabolites, terpenoids including the cyclic plant-type triterpenes constitute one of the largest and most diverse groups exhibiting important functions in plant physiology and development. For example, the linear triterpene precursor squalene plays a role in defense elicitation against herbivore attacks [2], the sterol cycloartenol is important for membrane functionality, plastid biogenesis and cell viability [3], β-amyrin is involved in root development [4], marneral affects general plant growth and development [5], and lupeol is essential for root nodule formation [6]. A group of glycosylated triterpenes, referred to as saponins, can act, for instance, as natural barriers against pathogenic microbial penetration due to their detergent-like characteristics (reviewed in [7]). Furthermore, triterpenes represent commercial potential in the pharmaceutical sector due to their bioactivities such as antiviral, anticancer, anti-inflammatory or wound-healing properties [8–13]. Antifertility effects of lupeol were recently demonstrated [14] and contraceptive application is discussed.

Like all terpenes, plant triterpenes are synthesized from C_5_ isoprene units. As a first step in the formation of triterpenes, the NAD(P)H-dependent enzyme squalene synthase (SQS) catalyzes the formation of the linear C_30_ isoprenoid squalene from C_15_ farnesyl pyrophosphate (FPP) by condensation of two FPPs to presqualene pyrophosphate and a subsequent reductive rearrangement to form squalene [15]. Subsequent squalene epoxidase (SQE)-catalyzed oxygenation of squalene yields 2,3-oxidosqualene, which is the linear key precursor of cyclic plant type triterpenes. SQE is a flavin-binding monooxygenase which requires NADPH and molecular oxygen to oxidize the substrate, and is reported to obtain electrons from cytochrome P450 reductase [16]. 2,3-oxidosqualene, in turn, can be further converted by diverse oxidosqualene cyclases (OSC) to a multitude of cyclic products. These plant triterpene products can be divided into sterol type molecules, which are generated via substrate folding into a transition state with chair-boat-chair (CBC) conformation, and the large group of triterpenes whose multiple skeletons are generated after substrate folding into a chair-chair-chair (CCC) conformation. A portion of the cyclic plant triterpene scaffolds is subsequently oxidized and further decorated thereby generating a larger structural diversity [17].

Consistent with the wide array of triterpene bioactivities, there is considerable effort in developing strategies for pathway identification and characterization of the corresponding enzymatic activities [18]. However, heterologous biosynthesis of cyclic plant-type triterpenes in microbial cells was thus far largely restricted to yeast systems, most notably *Saccharomyces cerevisiae* [17], and could only recently also be demonstrated for the triterpene dammarenediol-II in *Escherichia coli* [19]. To broaden the range of microbial systems for the functional heterologous biosynthesis of plant triterpenes, we chose to employ the physiologically versatile photosynthetic bacteria *Rhodobacter capsulatus* SB1003 and *Synechocystis* sp. PCC 6803 (hereafter: *Synechocystis*) as host strains. Both are well-established model organisms for anaerobic and aerobic bacterial photosynthesis, respectively. They have both gained attention as biotechnological production hosts for a variety of biochemicals [20, 21] and their plant growth promoting properties are of interest in the agricultural sector [22, 23]. Engineered strains synthesizing bioactive compounds in an agricultural context may contribute to novel farming strategies. Both bacteria represent promising platform hosts for triterpene biosynthesis due to unique phototrophy-associated physiological properties, i.e. a large intracellular membrane system, which can incorporate hydrophobic compounds and membrane-bound or -associated enzymes. In addition, both strains intrinsically harbor an effective isoprene metabolism for biosynthesis of carotenoids that serve as photopigments [24–26]. Isoprenoid pathway engineering has been demonstrated for different cyanobacterial and *Rhodobacter* strains, resulting in significant accumulation of hemi- (C_5_), mono- (C_10_), sesqui- (C_15_), di- (C_20_), tri- (C_30_) and tetraterpenes (C_40_) [27–35]. Recently, implementation of squalene biosynthesis has been demonstrated in both *R*. *capsulatus* [36] and *Synechocystis* [27, 37]. Notably, to enable C30 isoprenoid biosynthesis in *R*. *capsulatus*, heterologous expression of an SQS enzyme is essential [36]. In contrast, the natural hopanoid pathway of *Synechocystis* can be modified by disrupting the squalene hopene cyclase gene *shc* to accumulate squalene [37].

Here, we present for the first time the heterologous biosynthesis of the key plant triterpene precursor 2,3-oxidosqualene in the phototrophic prokaryotes *R*. *capsulatus* and *Synechocystis* by additional expression of SQE [38] in squalene producing cells. Further, the ability of both strains to accumulate cyclic plant triterpene compounds was evaluated by co-expression of selected OSC enzymes cycloartenol synthase CAS1 [39], lupeol synthase LUP1 [40], thalianol synthase THAS1 [41], and marneral synthase MRN1 [42], all derived from the model plant *Arabidopsis thaliana*.

## Materials and methods

### Bacterial strains and cultivation conditions

*Escherichia coli* strains DH5α [43], NEB5α (New England Biolabs), J53 [44] and S17-1 [45], used for cloning and conjugation, respectively, were cultivated on LB-agar plates or in liquid LB medium (Luria/Miller, Carl Roth) at 37 °C. Antibiotics were added to *E*. *coli* culture medium to the following final concentrations [μg mL^-1^]: 100 (ampicillin), 50 (kanamycin), 20 (spectinomycin). The photosynthetic purple non-sulfur α-proteobacterium *R*. *capsulatus* SB1003 [46] was cultivated on 2% (w/v) agar (Bacto agar; Difco) containing PY plates [47] or in RCV liquid medium [48] at 30 °C. The non-motile, glucose-tolerant (GT) strain of *Synechocystis* sp. PCC 6803 as well as the corresponding mutant strain Δ*shc* (*slr2089*) used in this study were kindly provided by Pia Lindberg (Uppsala University, Sweden) and cultivated on 0.75% (w/v) agar (Bacto agar; Difco) plates containing BG-11 mineral medium or in BG-11 liquid medium [49] at 30 °C. *Saccharomyces cerevisiae* strain GIL77 (gal2 hem3-6 erg7 ura3-167) [50], carrying derivatives of vector pYES/DEST-52 (Invitrogen) with MRN1 or THAS1, or as empty vector control [51], were cultivated at 30 °C using synthetic complete medium without uracil, supplemented with glucose, ergosterol, hemin, and Tween, as previously described [52].

### Construction of expression vectors

*A*. *thaliana* derived genes *SQS1* (At4g34640), *SQE1* (At1g58440), *CAS1* (At2g07050), *LUP1* (At1g78970), *THAS1* (At5g48010), and *MRN1* (At5g42600) were obtained as synthetic DNA by Eurofins Genomics with adapted codon-usage for expression in both bacterial hosts, and with RBS and restriction endonuclease recognition sequences where appropriate for initial cloning steps. An overview of constructs and genetic features, as well as primers used in this study is given in **Table 1** and **S1 Table**, respectively. All constructs were verified by sequencing of relevant sequences (performed as commercial service by Eurofins Genomics, Seqlab-Microsynth or Sequiserve), as are specified in **S2 Table**. Plasmid maps are shown in **S1 Fig.**

**Table 1:** Strain constructs used in this study and their genetic features.

strain	plasmid insert	Promoter	RBS1	CDS1	RBS2	CDS2	RBS3	CDS3
**Rc**	SQS1	P*nif*	pRhotHi-2	*SQS1*	-	-	-	-
	SQS1-SQE1			*SQS1*	pET	*SQE1*	-	-
	CAS1-SQS1-SQE1			*CAS1*	*nifK*	*SQS1*	pET	*SQE1*
	LUP1-SQS1-SQE1			*LUP1*				
	THAS1-SQS1-SQE1			*THAS1*				
	MRN1-SQS1-SQE1			*MRN1*				
**Syn**	SQE1	P*coaT*	*coaT*	SQE1*	-	*-*	-	*-*
	SQE1-CAS1				BBa0034	*CAS1*	-	-
	SQE1-LUP1					*LUP1*	-	-
	SQE1-THAS1					*THAS1*	-	-
	SQE1-MRN1					*MRN1*	-	-

Rc, *Rhodobacter capsulatus* SB1003; Syn, *Synechocystis* sp. PCC 6803; CDS, coding sequence; all Rc expression plasmids were based on pRhon5Hi-2, a derivative of pRhotHi-2 carrying the host-specific P*nif* promoter (NCBI Genbank Accession MG208548); all Syn expression plasmids were based on pVZ-spec carrying the host-specific P*coaT* promoter from pJPVCS (NCBI Genbank Accession MG191280). DNA sequences of recombinant insert sequences are specified in **S2 Table**.

*an *Nhe*I site was introduced to the 2nd and 3rd codon of *A*.*t*. *SQE1*.

#### Vectors for expression in *R*. *capsulatus*

Genes were cloned into expression vector pRhon5Hi-2, which is based on the expression vector pRhotHi-2 [53]. It harbors the native *R*. *capsulatus* promotor of the nitrogenase *nif* genes (P*nif*), which was inserted as an *Nhe*I/*Xba*I-fragment (NCBI Genbank Accession MG208548; Özgür, Drepper et al, unpublished) into the respective sites of vector pRhotHi-2 [53]. Use of this promoter enables tight regulation via ammonia provision or limitation.

Squalene synthase gene *SQS1*, which was designed as a synthetic gene with an *Nde*I-site at the 5’-end as well as an *Xho*I- and *Hind*III-site at the 3’-end, was cloned into the vector as an *Nde*I/*Hind*III fragment, creating pRhon5Hi-2-SQS1. To generate vector pRhon5Hi-2-SQS1-SQE1, both genes were first cloned in pUC18 successively, using *Nde*I/*Hind*III for *SQS1*, and *Xho*I/*Hind*III for *SQE1* with the pET vector RBS. The SQS1-SQE1 cassette was excised and cloned into the expression vector as *Nde*I/*Hind*III fragment. For construction of expression cassettes with the structure OSC-SQS1-SQE1, OSC genes were PCR-amplified for introduction into the expression vector pRhon5Hi-2-SQS1-SQE1 upstream of *SQS1*. For cloning of *CAS1*, *LUP1* and *MRN1*, the synthetic DNA fragments were used as templates, and PCR primers (#510 - #515) were adapted to introduce *Nde*I sites on both ends to enable cloning at the first position of the operon, as well as the RBS of *R*. *capsulatus nifK* at the 3’ end of the fragment for the downstream gene *SQS1*. For cloning of *THAS1*, vector pRhon5Hi-2-H6-THAS1-SQS1-SQE1 was used as template (find sequence in **S2 Table**) for amplification of the synthetic gene together with the *nifK* RBS (using primers #516/ #517), which already carried the synthetic OSC gene with the following *nifK* RBS in front of the *SQS1* gene. Therefore, each gene was equipped with its own RBS, as specified in **Table 1** and **S2 Table**: The first operon gene was preceded by the RBS of the pRhotHi-2 vector, *SQE1* was cloned with the pET vector RBS, and in OSC-containing constructs, *SQS1* was equipped with the *nifK* RBS from *R*. *capsulatus*.

#### Vectors for expression in *Synechocystis*

Expression plasmids for *Synechocystis* were all derivatives of the conjugative shuttle vector pVZ-spec [54, 55]. As basic cloning backbone, a pJET1.2 (Thermo Scientific) -based cloning plasmid pJPVCS (NCBI Genbank Accession MG191280; Schmelling, Dienst et al., unpublished) was used that harbored the native cobalt-inducible promoter P*coaT* from *Synechocystis* including the upstream-located repressor gene *coaR*. The promoter-5’UTR sequence of *coaT* was fused to the mVenus CDS via an *Nhe*I site constituting the 2^nd^ and 3^rd^ codon (Ala-Ser). The whole expression cassette was flanked by the Biobrick prefix and suffix, which were further flanked by *Nsi*I- and *Sal*I sites, respectively, on this plasmid.

For construction of plasmids pVZ-PcoaT-SQE1-MRN1 and pVZ-PcoaT-SQE1-THAS1, the sequential cassettes SQE1-MRN1 and SQE1-THAS1 were PCR-amplified from plasmids pRhofHi2-SQS1-SQE1-MRN1 and pRhofHi2-SQS1-SQE1-THAS1 (Loeschcke, unpublished), carrying the synthetic plant genes with bacterial codon usage (as specified in **S2 Table**), respectively. The used primers were #252 (introducing an *Nhe*I site to the 2^nd^ and 3^rd^ codon of SQE1) and #253 (adding transcriptional terminator T7 106 bp downstream of the stop codon and an *Spe*I site to the 3’terminus of the amplicon). Both PCR products were digested with *Spe*I/ *Nhe*I and ligated into the correspondingly cut plasmid pJPVCS, thereby placing *SQE1* downstream of the *coaR*-P*coaT*-5’UTR-construct. Translation of the OSC genes was mediated by the Biobrick RBS BBa_0034. The expression cassette was excised by *Nsi*I/ *Sal*I digestion and transferred to the *Pst*I/ *Sal*I-cut pVZ-spec plasmid. Cloning of plasmid pVZ-PcoaT-SQE1 was based on a gBlock fragment (IDT) harboring the 3’-terminal 408 bp of *SQE1* (starting with the CDS´s *Age*I site at pos. 2794), the T7 terminator with a 29 bp spacer to the stop codon and a 3’-terminal *Spe*I site. The fragment was digested with *Age*I and *Spe*I and ligated in the correspondingly cut vector pJET-PcoaT-SQE1-MRN1, thereby excising the *MRN1* cassette including the contiguous T7 terminator. The *coaR*-P*coaT*-5’UTR-SQE1 construct was excised from the pJET backbone by *Nsi*I/ *Sal*I digestion and transferred to the *Pst*I/ *Sal*I-cut pVZ-spec plasmid. For construction of plasmids pVZ-PcoaT-SQE1-CAS1 and pVZ-PcoaT-SQE1-LUP1, we used the AQUA (advanced quick assembly) technique [56] using plasmid pJET-PcoaT-SQE1-MRN1 as vector backbone. In order to exchange *MRN1* with *CAS1*, AQUA cloning was performed with each three overlapping PCR fragments, generated with primer pairs #531/ #532, #528/ #529 and #526/ #527. For corresponding *LUP1* cloning, primer pairs #533/ #532, #528/ #530 and #524/ #525 were used to amplify the AQUA fragments, before the assembly reaction was conducted in *E*. *coli* strain NEB5α. From the resulting plasmids, pJET-PcoaT-SQE1-CAS1 and pJET-PcoaT-SQE1-LUP1, inserts SQE1-CAS1 and SQE1-LUP1 were subsequently excised by *Nhe*I/ *Sal*I digestion and transferred to *Nhe*I/ *Sal*I digested plasmid pVZ-PcoaT-SQE1-MRN1.

### Heterologous expression of plant triterpene biosynthesis genes in *R*. *capsulatus*

For introduction of *A*. *thaliana* triterpene biosynthesis genes in *R*. *capsulatus*, respective pRhon5Hi-2-based plasmids were transferred to the host via conjunctional transfer as previously described [47]. Thereafter, exconjugants were selected and further cultivated on PY agar, containing 25 μg mL^-1^ kanamycin and 25 μg mL^-1^ rifampicin.

For heterologous expression of triterpene biosynthesis genes, cultivation was conducted in liquid RCV medium with 4g/L malate containing 25 μg mL^-1^ kanamycin and 25 μg mL^-1^ rifampicin under non-phototrophic, micro-aerobic conditions (which is sufficient for the induction of carotenoid synthesis and formation of the intracellular membrane system in this organism) in 100 mL Erlenmeyer flasks at 30°C under permanent agitation at 130 rpm in a Multitron Standard incubation shaker (Infors HT) in the dark. Pre-cultures of 35 mL RCV medium containing 0.1% (NH_4_)_2_SO_4_ were inoculated and incubated for 48 h. Expression cultures were inoculated from pre-cultures to an initial OD_660nm_ of 0.05 in 60 mL RCV medium containing 0.1% serine as sole nitrogen source. The omission of ammonium together with implementation of micro-aerobic conditions ensured derepression of the P_*nif*_ promoter and target protein expression. After incubation of cultures for 2 days (52 h), when an OD_660nm_ of ~1.5 was reached, cell samples corresponding to OD_660nm_ = 15 were harvested by centrifugation (10’, 1780 g, 4°C) and pellets stored at -20 °C.

### Heterologous expression of plant triterpene biosynthesis genes in *Synechocystis*

For expression of plant triterpene biosynthesis genes in *Synechocystis*, pVZ-based expression plasmids were transferred to strain Δ*shc* by conjugation [55], whereupon exconjugants were selected and further cultivated on BG11 agar containing, 20 μg mL^-1^ spectinomycin and 40 μg mL^-1^ kanamycin_._

For triterpene biosynthesis experiments, cells were grown under continuous illumination with white light (50 μmol photons m^–2^ s^–1^) at 30 °C and 150 rpm in a New Brunswick Innova42 incubator shaker (Eppendorf). The culture volume was 80 mL within 250 mL Erlenmeyer flasks. Precultures were inoculated in BG-11 medium containing 20 μg mL^-1^ spectinomycin and 40 μg mL^-1^ kanamycin, and 10 mM TES buffer (pH 8.0) without cobalt supply, in order to repress gene expression from promoter P_coaT_. For induction, cultures were supplemented with 20 μM CoCl_2_ at an optical density (OD_750nm_) of ~0.4 (Specord 200 PLUS UV/Vis spectrophotometer, Analytik Jena). After cultivation for 4 days in cobalt-repleted medium, when cultures reached an OD_750nm_ of ~1.0, cells were harvested from 20 mL culture portions by centrifugation (10 min, 4 °C and 4800 g). Resulting pellets were frozen in liquid nitrogen and stored at -80 °C.

### Analysis of *R*. *capsulatus* growth, pigmentation, and dry cell weight

Growth of *R*. *capsulatus* in expression cultures was measured as optical cell densities at 660 nm, and pigmentation by whole cell absorbance spectra determined semi-daily during the cultivation (GENESYS 10S UV-Vis-Spectrophotometer, Thermo Scientific). For measurement of absorbance spectra, cell samples were washed with water to remove rifampicin in order to avoid interference by the red colored antibiotic, and spectra of whole cells in water were recorded from 300 to 900 nm and cell-density normalized. Carotenoid-specific absorption at 500 nm was extracted from the data.

To determine the correlation of dry cell weight (DCW) of *R*. *capsulatus* grown micro-aerobically in RCV medium with antibiotics and serine to measured optical cell density at 660 nm, six differently concentrated samples of cells were harvested from expression cultures by centrifugation. Supernatants were discarded and pellets were dried at 30 °C for one hour under vacuum in a Concentrator 5301 (Eppendorf). Differential weighing of sample tubes before and after filling and drying was employed to determine cell weight. An influence of the drying procedure on the weight of sample tubes was excluded by subjecting empty control tubes to the same treatment. Linear regression enabled determining a correlation of 1 mL OD_660nm_ = 1 with 0.58 mgDCW.

### Analysis of *Synechocystis* growth, pigmentation, and dry cell weight

Growth of *Synechocystis* cultures was monitored by semi-daily measurement of the OD_750nm_ until the end of the cultivation period. Whole cell absorbance spectra of *Synechocystis* cells in the clear medium were recorded from 400 to 750 nm on a Specord 200 PLUS spectrophotometer (Analytik Jena) and were normalized to OD_750nm_. Carotenoid-specific absorption at 500 nm was extracted.

For determination of DCW of *Synechocystis*, cells of 25 mL of liquid cultures were collected on Supor 0.45-μm membrane filters (Pall) by vacuum filtration and incubated at 60 °C. The filters were weighed before and after cell collection. To exclude effects from desiccation of the filter material, empty control filters were treated the same way. Linear regression enabled determining a correlation of 1 mL OD_750nm_ = 1 with 0.44 mg DCW.

### Extraction of triterpenes from *R*. *capsulatus* and *Synechocystis*

For extraction of triterpenes from bacterial cells, all organic solvents (acetone, n-hexane, and chloroform/methanol (2:1)) were supplemented with 0.05% (w/v) butylated hydroxytoluene (BHT). Frozen cell pellets were extracted with acetone that was afore supplemented with a total of 10 nmol β-sitosterol as internal standard. *R*. *capsulatus* cell samples (equivalent to OD_660nm_ = 15) were extracted twice with 0.7 mL acetone, *Synechocystis* cell samples (equivalent to OD_750nm_ = 20) were extracted twice with 1 mL acetone under gentle agitation at 50 °C for 15 min. After centrifugation (3 min at 1780 g and RT), the supernatants were transferred to a fresh tube, supplemented with 1.5 mL 1M NaCl (*R*. *capsulatus*) or 1 mL 1M NaCl (*Synechocystis*) and mixed. Samples were subsequently extracted twice with a total volume of 1.8 ml n-hexane by vigorous mixing for 30 sec. To collect the (upper) hexane phase, samples were centrifuged for phase separation (1 min at 1780 g and RT), and n-hexane extracts were transferred to a fresh tube before drying in a vacuum centrifuge for 20 min at 30 °C. The dried extracts were resuspended in 150 μL chloroform/methanol (2:1), transferred into HPLC vials and stored at -20 °C until analysis.

### Extraction of triterpenes from *S*. *cerevisiae*

To obtain analytic references for marnerol, hydroxymarnerol and thalianol, lanosterol synthase deficient *S*. *cerevisiae* GIL77, carrying pYES/DEST-52 with *MRN1*, *THAS1* or as empty vector control [51], was cultivated in 400 mL batches for plant triterpene production as previously described [52], utilizing galactose supplemented medium for gene expression. After cultivation, 15 mL portions were harvested and pellets stored at -80 °C until extraction. Pellets were incubated with 2 mL 20% (w/v) KOH in 50% (v/v) EtOH for 30 min at 70 °C, prior to extraction of triterpenes with 2 mL n-hexane. 100 μL of the n-hexane phase were dried under a stream of nitrogen gas and re-dissolved in methanol for LC-MS (liquid chromatography–mass spectrometry) analysis.

### LC-MS analysis of triterpenes

Extracts from *Synechocystis* and *R*. *capsulatus*, dissolved in chloroform/methanol (2:1) with 0.05% BHT, were diluted 1:100 with methanol (+ 0.05% BHT) prior to injection of 10 μl sample volumes. Triterpenes were separated on a Dionex HPG 3200 HPLC system (Thermo Scientific) equipped with a 150 x 2.1 mm, 2.7 μm, Cortecs C8 column (Waters) with a binary gradient system. Mobile phase A consisted of water + 0.1% formic acid (FA) and mobile phase B consisted of methanol + 0.1% FA. The mobile phase gradient was as follows: Starting conditions were 75% mobile phase B, increased to 85% B within 4 min and then further increased to 100% B in 13.5 min, the plateau was held for another 4.5 min and the system was returned to starting conditions within 3 min. The flow rate was 0.5 mL/min. Triterpenes were analyzed by Q-TOF (quadrupole-time-of-flight) MS and MS/MS on a maXis 4G instrument (Bruker Daltonics) equipped with an ESI (electrospray ionization) source. The instrument was operated in positive-ion mode and the operating conditions were as follows: dry gas (nitrogen): 8.0 L/min, dry heater: 220 °C, nebulizer pressure: 1.8 bar, capillary voltage: 4500 V.

### Quantification of triterpenes

Squalene, 2,3-oxidosqualene, cycloartenol and lupeol were quantified using calibration curves obtained with commercial references (Sigma-aldrich). β-sitosterol (Sigma-aldrich) was used as an internal standard. Triterpene production was calculated based on LC-MS data as product titer in the culture (mg L^-1^), as cell-density normalized specific titer (mg L^-1^ OD^-1^), and as specific yield per dry cell weight (mg gDCW^-1^), all determined as end point values after the cultivation duration of 52 h (*R*. *capsulatus*) and 168 h (i.e. 116 h after cobalt-induction; *Synechocystis*), respectively. In addition, the volumetric and specific productivities (μg L^-1^ h^-1^ and μg gDCW^-1^ h^-1^) were calculated as averaged values per h cultivation. Squalene productivity in *Synechocystis* was calculated for the complete cultivation duration (168 h), since squalene production was not induced. Productivity for further triterpenes was calculated in reference to the 116 h period after induction.

## Results

### Pathway design for triterpene biosynthesis in *R*. *capsulatus* and *Synechocystis*

**Fig 1:**
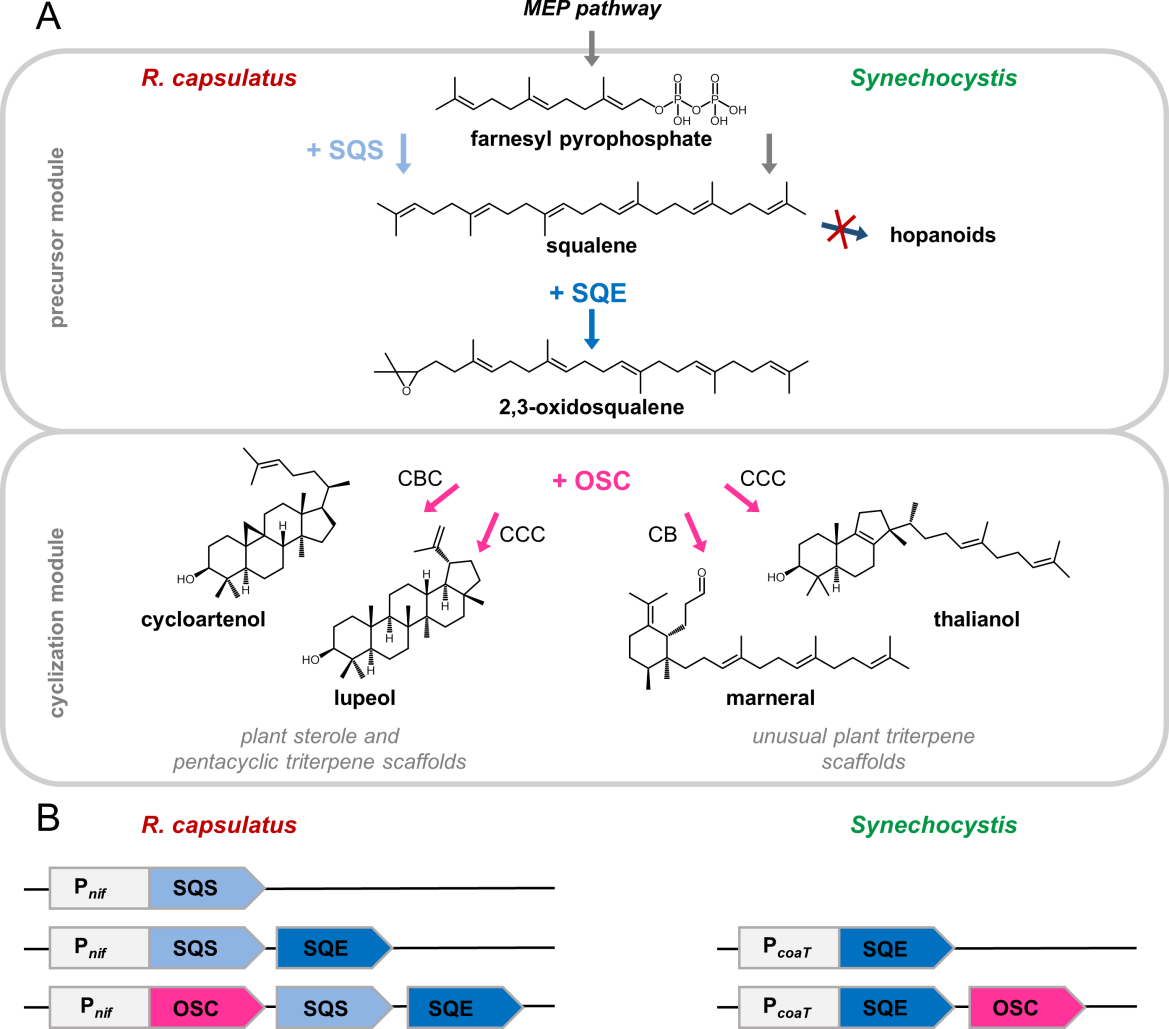
Triterpene pathway design. **(A)** Pathways for targeted biosynthesis of triterpenes in *R*. *capsulatus* and *Synechocystis* by implementation of *A*. *thaliana* biosynthesis modules. In both organisms, the common triterpene precursor FPP is provided by the native 2-C-methyl-D-erythritol-4-phosphate (MEP) pathway. To establish the first step of the triterpene-specific precursor module in *Synechocystis*, a knock-out mutant of the *shc* gene encoding the squalene-hopene cyclase of the intrinsic hopanoid biosynthesis was employed [37]. In *R*. *capsulatus*, heterologous expression of SQS1 was implemented to form squalene. For monooxygenation of squalene to the central precursor 2,3-oxidosqualene, SQE1 was heterologously expressed in both host strains. Subsequently, the cyclization module was designed to convert the linear precursor 2,3-oxidosqualene into plant sterols and further cyclic triterpenes. To cover different triterpene scaffolds, the OSC enzymes CAS1, LUP1, THAS1, and MRN1 from *A*. *thaliana* were expressed in each host to synthesize cycloartenol (sterol), lupeol (exhibiting the often occurring pentacyclic scaffold) as well as thalianol and marneral (representing more unusual tri- or monocyclic structures). Respective substrate folding is indicated (CBC, chair-boat-chair; CCC, chair-chair-chair; CB, chair-boat). **(B)** Schematic representation of expression constructs used for triterpenoid biosynthesis in *R*. *capsulatus* and *Synechocystis*. The respective genes were arranged as synthetic operons, using native ammonium (P_*nif*_) and cobalt (P_*coaT*_) regulated promoters for transcription activation. FPP, farnesyl pyrophosphate; SQS, squalene synthase; SQE, squalene epoxidase; OSC, oxidosqualene cyclase.

To implement different plant triterpene biosynthetic pathways in *R*. *capsulatus* and *Synechocystis*, we aimed to link the intrinsic isoprene metabolism of the phototrophic microbes to heterologous pathway modules, introduced as codon optimized genes from *A*. *thaliana* (**Fig 1**).

*R*. *capsulatus* harbors the 2-C-methyl-D-erythritol-4-phosphate (MEP) pathway leading to the formation of isoprene units and thereof derived FPP, which is a natural intermediate in biosynthesis of the tetraterpenes spheroidene and spheroidenone [20]. We first introduced *SQS1* (original cDNA sequence: At4g34640 [57]) encoding squalene synthase 1 in order to redirect the flux of FPP toward heterologous squalene production. In *Synechocystis*, the MEP pathway provides precursors for the biosynthesis of several carotenoids including β-carotene, echinenone and a set of xanthophylls [58]. Moreover, the pathway is directly linked to native squalene biosynthesis, leading to the formation of hopanoids. For this work, we therefore used the recently described squalene hopene cyclase deficient Δ*shc* mutant, which is characterized by squalene accumulation [37]. For subsequent squalene oxygenation and production of the triterpene precursor 2,3-oxidosqualene, squalene epoxidase 1 encoding *SQE1* (At1g58440 [38]) was introduced into both hosts.

The ability of the chosen host organisms to synthesize different triterpene scaffolds was evaluated by introducing four OSC enzymes, namely cycloartenol synthase 1, lupeol synthase 1, thalianol synthase 1 or marneral synthase 1, encoded by *CAS1* (At2g07050 [39]), *LUP1* (At1g78970 [40]), *THAS1* (At5g48010 [41]), and *MRN1* (At5g42600 [42]), respectively. While cycloartenol and lupeol represent typical plant triterpenes with tetracyclic plant sterol and pentacyclic plant triterpene scaffolds, respectively, thalianol and marneral exhibit more unusual tri- and monocyclic structures (**Fig 1A**).

For heterologous expression, synthetic operons were successively assembled encompassing the pathway genes as well as host-specific promoters and regulatory elements **(Fig 1B**). In *R*. *capsulatus*, we used the expression plasmid pRhon5Hi-2, which harbors the native NH_4_^+^-depletion inducible P_*nif*_ of the nitrogenase-encoding *nifHDK* operon. In *Synechocystis*, joint expression of *SQE1* and the OSC genes was mediated by the native Co^2+^-inducible promoter P_*coaT*_ of the Co^2+^ efflux system-encoding *coaT* gene [59] on plasmid pVZ-spec [54, 60]. The sequences of the synthetic genes and relevant operon elements are given in **S2 Table** and a graphic representation of the plasmids is shown in **S1 Fig**.

After plasmid transfer, the *R*. *capsulatus* and *Synechocystis* strains were cultivated in liquid medium as appropriate for respective expression cultures. Cells were harvested in the early stationary (after 52 h, *R*. *capsulatus*), or the late logarithmic growth phase (after 168 h, i.e. 116 h after induction, *Synechocystis*) for LC-MS (liquid chromatography mass-spectrometry) based triterpene analysis.

### Implementation of triterpene precursor supply: squalene and 2,3-oxidosqualene

**Fig 2:**
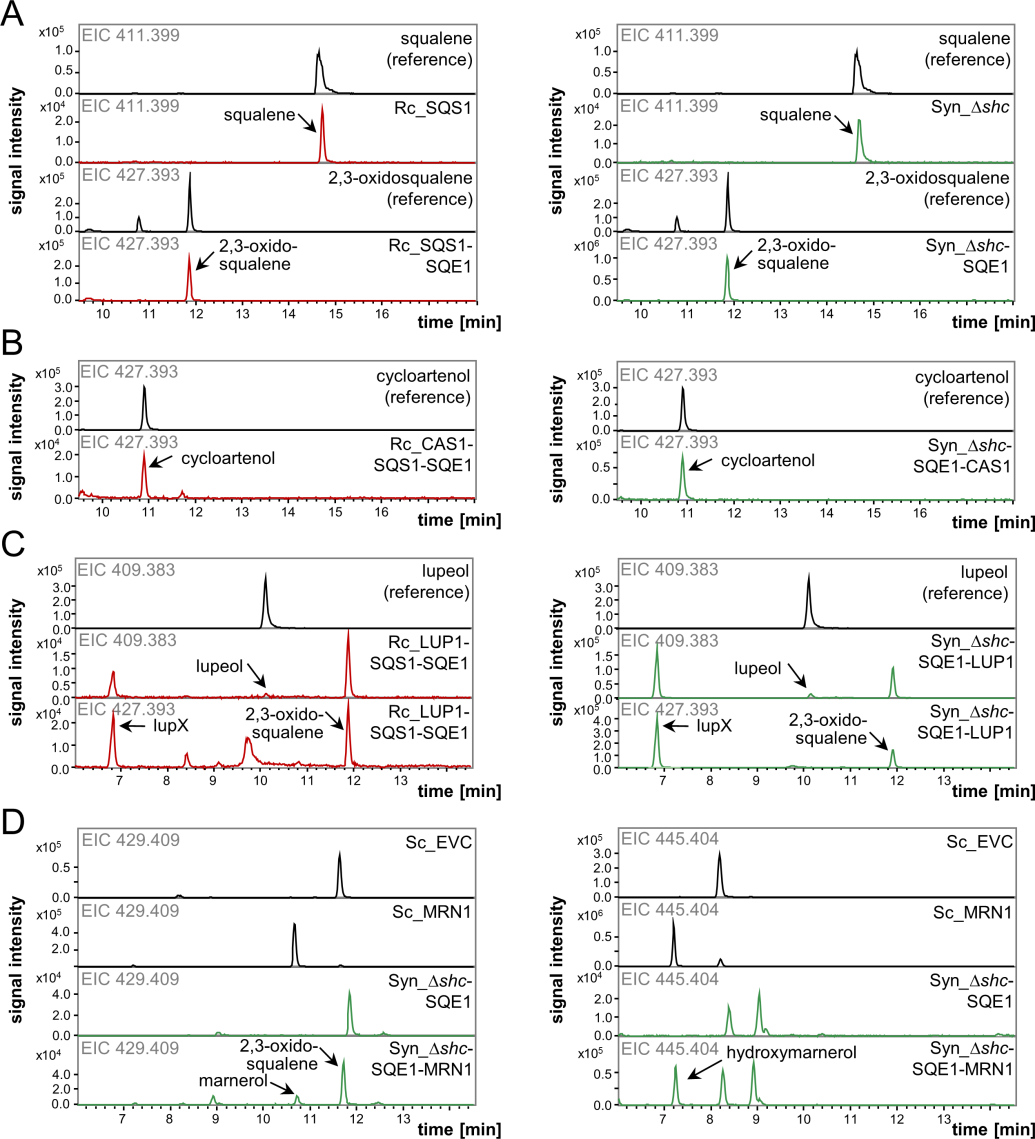
LC-MS detection of triterpenoids produced in engineered *R*. *capsulatus* SB1003 (Rc) and *Synechocystis* sp. PCC 6803 (Syn) strains. Cells of the respective strains (indicated in chromatograms and color-coded with red for Rc-, green for Syn-strains) were subjected to extraction and analysis after expression of *A*. *thaliana* triterpenoid biosynthesis genes (SQS1, squalene synthase; SQE1, squalene epoxidase; CAS1, cycloartenol synthase; LUP1, lupeol synthase; MRN1, marneral synthase). Triterpenoid identity was verified by comparison of retention times, as well as MS and MS/MS spectra to commercial or biological references (**S2 Fig**). **(A)** Signals in EICs of m/z 411.399 at RT 14.9 min correspond to squalene (C_30_H_50_), signals in EICs of m/z 427.393 at RT 11.8 min correspond to 2,3-oxidosqualene (C_30_H_50_O). **(B)** Signals in EICs of m/z 427.393 at RT 10.9 min correspond to cycloartenol (C_30_H_50_O). **(C)** Signals in EICs of m/z 409.383 at RT 9.9 min correspond to lupeol (C_30_H_50_O-H_2_O). Signals in EICs of m/z 427.393 (and 409.383) at RT 6.7 min correspond to lupX (tentatively identified as lupanediol (C_30_H_52_O_2_-H_2_O)). In both EICs, signals at RT 11.7 min correspond to 2,3-oxidosqualene (C_30_H_50_O). **(D)** Signals in EICs of m/z 429.409 at RT 10.7 min correspond to marnerol (C_30_H_52_O), signals in EICs of m/z 445.404 at RT 7.1 min correspond to hydroxymarnerol (C_30_H_52_O_2_). Signals in EICs of m/z 429.409 at RT 11.8 min correspond to the (M+2) isotopic peak of 2,3-oxidosqualene (C_30_H_52_O). Peaks in EICs of m/z 445.404 also detected in Syn*_*Δ*shc*-SQE1 (control) also correspond to 2,3-oxidosqualene-derived signals. As a reference, chromatograms of samples from *S*. *cerevisiae* GIL77 (Sc), carrying pYES/DEST-52 with *MRN1* or as empty vector control (EVC), are shown. Shown chromatograms are representative for replicate measurements from at least three independent cultivations. The corresponding quantitative data are summarized in **Table 2**.

In order to assess the applicability of *R*. *capsulatus* and *Synechocystis* for the biosynthesis of cyclic plant triterpenes, their potentials for accumulating the linear precursors squalene and 2,3-oxidosqualene were determined. To this end, cell extracts of strains Rc_SQS1 and Syn_Δ*shc* were analyzed for squalene accumulation by LC-MS. Specific accumulation of squalene could clearly be determined in specific extracted ion chromatograms (EICs) in both production strains (**Fig 2A**, two upper panels), and the identification was further validated by comparison of MS fragment spectra (MS/MS spectra) with a commercial standard (**S2 Fig**). Quantitative analysis revealed comparable specific squalene yields of 9.44 mg gDCW^-1^ and 9.70 mg gDCW^-1^ in Rc_SQS1 and Syn_Δ*shc*, respectively. Besides specific yields, also product titers, specific titers and productivities were calculated to enable consideration of respective cell masses, culture volumes, and times required for production (**Table 2**).

**Table 2:** Triterpene levels in engineered *R*. *capsulatus* SB1003 (Rc) and *Synechocystis* sp. PCC 6803 (Syn) strains.

host bacterium	compound	specific yield mg gDCW^-1^	product titer mg L^-1^	specific titer mg L^-1^OD^-1^	volumetric productivity μg L^-1^h^-1^	specific productivity μg gDCW^-1^h^-1^	precursor conversion
**Rc**	squalene	9.44 ± 3.17	8.24 ± 3.06	5.50 ± 1.84	171.43 ± 58.80	181.51 ± 60.87	-
	2,3-oxido-squalene	0.32 ± 0.03	0.28 ± 0.02	0.19 ± 0.02	5.33 ± 0.45	6.17 ± 0.52	quantitative
	cycloartenol	0.40 ± 0.08	0.34 ± 0.06	0.24 ± 0.05	6.47 ± 1.24	7.77 ± 1.56	quantitative
	lupeol	minor amounts of lupeol and proposed hydroxylated lupeol derivative detected					poor
	thalianol	not detected					-
	marneral	not detected					-
**Syn**	squalene	9.70 ± 0.59	5.08 ± 0.59	4.31 ± 0.26	30.71 ± 3.26	58.65 ± 3.08	-
	2,3-oxido-squalene	2.09 ± 0.97	1.13 ± 0.55	0.93 ± 0.43	9.81 ± 4.81	18.22 ± 8.51	quantitative
	cycloartenol	2.06 ± 0.10	1.10 ± 0.05	0.92 ± 0.04	9.67 ± 0.44	18.11 ± 0.86	quantitative
	lupeol	0.29 mg/gDCW lupeol and proposed hydroxylated lupeol derivative detected					good
	thalianol	not detected					-
	marneral	marnerol and hydroxymarnerol detected					poor

Data represent mean values and respective standard deviations from three independent cultivations. Squalene levels were determined in strains Rc_SQS1 and Syn_Δ*shc*, as well as 2,3-oxidosqualene levels in Rc_SQS1-SQE1 and Syn_Δ*shc*-SQE1, and cycloartenol levels in Rc_CAS1-SQS1-SQE1 and Syn_Δ*shc*-SQE1-CAS1. Lupeol levels and synthesis of further triterpenes were determined in strains Rc_LUP1-SQS1-SQE1 and Syn_Δ*shc*-SQE1-LUP1. Strains Rc_THAS1-SQS1-SQE1 and Syn_Δ*shc*-SQE1-THAS1, as well as Rc_MRN1-SQS1-SQE1 and Syn_Δ*shc*-SQE1-MRN1 were analyzed for accumulation of thalianol and marneral, as well as derivatives thereof, respectively.

Moreover, strains Rc_SQS1-SQE1 and Syn_Δ*shc*-SQE1, additionally carrying the epoxidase, were assayed for 2,3-oxidosqualene accumulation which was detected (**Fig 2A**, two lower panels; **S2 Fig**) with final yields of 0.32 mg gDCW^-1^ and 2.09 mg gDCW^-1^, respectively (**Table 2**). Although these quantities correspond to only ~3% and ~20% of squalene yields previously observed without *SQE1* expression, amounts of the non-oxygenated precursor were reduced in these extracts below the limit of quantification, indicating effective epoxidation of squalene in both strains.

### Biosynthesis of the tetracyclic sterol cycloartenol catalyzed by CAS1

Cycloartenol is the common precursor for the biosynthesis of most sterol compounds in plants, which is synthesized via the CBC-type cyclization of 2,3-oxidosqualene resulting in the characteristic tetracyclic arrangement. For biosynthesis of cycloartenol in *R*. *capsulatus* and *Synechocystis*, strains Rc_CAS1-SQS1-SQE1 and Syn_Δ*shc*-SQE1-CAS1 were generated, expressing the precursor module genes together with the *CAS1* gene from *A*. *thaliana*.

The specific accumulation of cycloartenol in both strains was verified by LC-MS. Cycloartenol was identified by comparison of signal retention times in characteristic EICs (**Fig 2B**) and MS/MS spectra with a commercial reference (**S2 Fig**). Quantitative analysis revealed yields of 0.4 mg gDCW^-1^ and 2.06 mg gDCW^-1^ for Rc_CAS1-SQS1-SQE1 and Syn_Δ*shc*-SQE1-CAS1, respectively, basically reflecting 2,3-oxidosqualene levels in the preceding analysis of strains not expressing *CAS1* (**Table 2**). At the same time, no significant concomitant accumulation of the precursors squalene and 2,3-oxidosqualene could be detected (compare 2,3-oxidosqualene signals in strains not expressing *CAS1* in lower panels of **Fig 2A**, and absent signals in *CAS1* expressing strains in **Fig 2B**). Therefore, engineering the triterpenoid pathway toward cycloartenol apparently has led to a virtually quantitative cyclization of the linear precursor in both host strains.

### Biosynthesis of the pentacyclic triterpene lupeol and a putative derivative by LUP1

As a representative of canonical pentacyclic plant triterpenes, lupeol was chosen as target candidate. Lupeol is synthesized from 2,3-oxidosqualene via the CCC-type cyclization. However, the selected OSC from *A*. *thaliana*, LUP1, was shown previously to catalyze the formation of multiple scaffolds besides lupeol, including β-amyrin as well as triterpene alcohols and diols [40, 61]. Thus, the detection of a variety of further minor cyclization products was expected in strains Rc_LUP1-SQS1-SQE1 and Syn_Δ*shc*-SQE1-LUP1. LC-MS analyses of extracts were therefore expanded to the corresponding masses for alternative alcohol and diol compounds. As deducible from the EICs (**Fig 2C**, two upper panels) and respective MS as well as MS/MS spectra (**S2 Fig**) in comparison to the lupeol reference, both host strains accumulated lupeol. Here, the EIC of m/z 409.383 was chosen for representation, since this signal, corresponding to [M+H-H_2_O]^+^ resulting from loss of H_2_O (**S3 Table**) due to in-source decay during electrospray ionization was predominant. While in Rc_LUP1-SQS1-SQE1, amounts were below the limits of quantification, in Syn_Δ*shc*-SQE1-LUP1, the specific yield was 0.29 mg gDCW^-1^ (**Table 2**). In addition, a stronger signal for m/z 427.393, accompanied by lower signals for m/z 409.383 and m/z 467.386 was detected deviating in retention time (**Fig 2C**, lower panels) from the lupeol standard, likely corresponding to another product of LUP1-mediated cyclization. The emerging m/z were calculated to correspond to the [M+H-H_2_O]^+^ (427.393), [M+H-(H_2_O)_2_]^+^ (409.383) and [M+Na]^+^ (467.386) of a compound with the formula C_30_H_52_O_2_. The [M+H]^+^ was not detected, presumably due to in-source decay of the compound (**S2 Fig**). The retention time of the compound and deduced formula may point to lupanediol, which has previously been described as a product of LUP1 [61, 62]. However, since the structure of the proposed hydroxylated lupeol derivative cannot be unambiguously assigned from the data, it is designated as lupX at this point. While only trace amounts of both squalene and 2,3-oxidosqualene were detected in *Synechocystis* extracts, suggesting quantitative precursor cyclization by LUP1, residual 2,3-oxidosqualene in *R*. *capsulatus* (0.05 mg gDCW^-1^) indicates poorer conversion than observed with CAS1.

### Expression of THAS1 and MRN1 for biosynthesis of unusual tricyclic and seco-triterpenes

The OSC THAS1 from *A*. *thaliana* catalyzes the formation of thalianol via the CCC-type cyclization of 2,3-oxidosqualene, constituting an atypical triterpenoid scaffold, characterized by a tricyclic arrangement [41]. Extracts of strains Rc_THAS1-SQS1-SQE1 and Syn_Δ*shc*-SQE1-THAS1 were analyzed in comparison to samples from a reference yeast strain, i.e. *S*. *cerevisiae* GIL77 carrying pYES/DEST-52 with *THAS1*, which was previously shown to accumulate the product [51]. However, in neither of the engineered strains, the conversion of the substrate to thalianol or any other cyclic product was detected (**S2 Fig**). In *Synechocystis*, this is further evident from the observation that the 2,3-oxidosqualene levels were comparable to those the control strains expressing only *SQE1*. The substrate levels in *R*. *capsulatus* co-expressing the OSC were significantly lower than in the control expressing SQS1-SQE1. However, a direct comparison of both strains is not possible here, since in *R*. *capsulatus*, the OSC gene was placed upstream of the precursor module genes *SQS1* and *SQE1* in order to ensure high expression levels of the cyclase, probably at the expense of the downstream genes. The absence of a cyclic triterpene product in *THAS1*-expressing strains can be caused by diverse problems on multiple levels, including transcription, mRNA stability, translation, protein stability, and activity of the heterologous enzyme. However, this observation most probably points to difficulties at the level of enzyme folding or assembly, since limitations at DNA and transcription levels could be excluded by DNA sequencing as well as RT-qPCR (data not shown) or precursor accumulation (indicating concerted expression of all genes within the synthetic operon THAS1-SQS-SQE).

The OSC MRN1 from *A*. *thaliana* catalyzes the formation of the seco-triterpene marneral via an unusual CB substrate conformation, resulting in a further atypical, monocyclic triterpenoid structure [42]. LC-MS analysis revealed that the primary cyclization product marneral was not detected in either the extracts from Rc_MRN1-SQS1-SQE1 or Syn_Δ*shc*-SQE1-MRN1 (data not shown). While in the *R*. *capsulatus* extracts none of the expected products were detectable, strain Syn_SQE1-MRN1 apparently accumulated both marnerol (m/z = 429.409) and an additional product with m/z signals (m/z = 427.394, 445.404, 467.386) corresponding to C_30_H_52_O_2_, presumably hydroxymarnerol (**Fig 2D**, two lower panels and **S2 Fig**). Both marnerol and the putative hydroxymarnerol were also detected in a reference yeast strain expressing MRN1 [51] (**Fig 2D**, two upper panels and **S2 Fig**). However, still significant amounts of 2,3-oxidosqualene were present in the *Synechocystis* extracts, suggesting low efficiency of substrate cyclization by MRN1.

### Cell growth and pigmentation during triterpene biosynthesis

**Fig 3:**
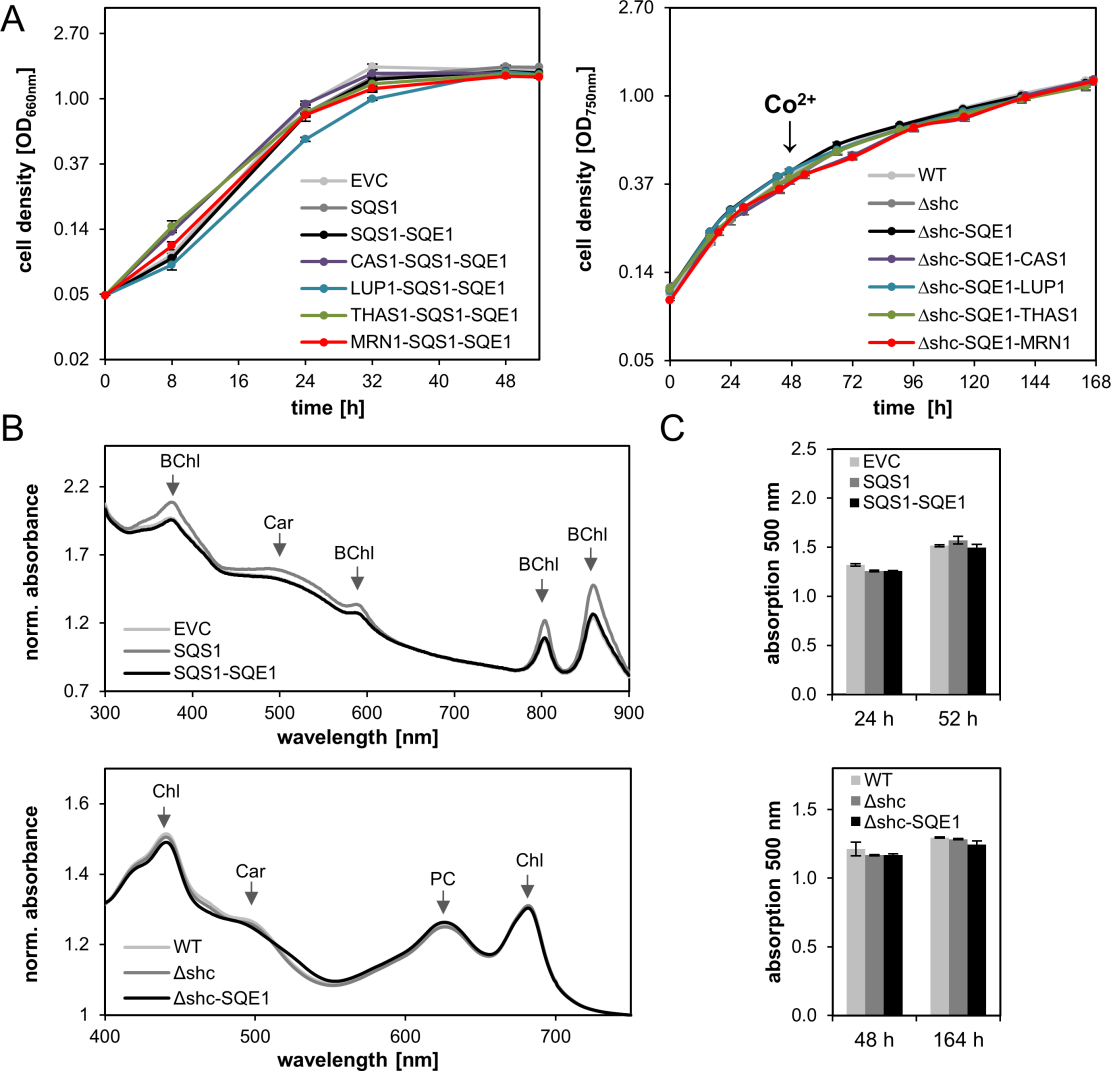
Comparative physiological characterization of triterpene producing strains of *R*. *capsulatus* and *Synechocystis*. **(A)** Growth of *R*. *capsulatus* (left) and *Synechocystis* (right) expressing *A*. *thaliana* triterpene biosynthesis genes. Cell densities were determined by turbidity measurements at 660 nm (*R*. *capsulatus*) or 750 nm (*Synechocystis*) to record growth curves. Strains carrying the precursor biosynthetic modules for the generation of squalene and 2,3-oxidosqualene were compared to strains that additionally harbored different cyclization modules or an empty vector as control. Data represent mean values from three independent cultivations, as well as the respective standard deviations. The time point of cobalt (Co^2+^)-induction in *Synechocystis* is indicated. The y-axes are scaled logarithmically (base *e*). **(B)** Whole cell absorbance spectra of *R*. *capsulatus* (upper panel) and *Synechocystis* (lower panel) expressing the precursor synthesis modules. Pigment profiles were recorded at time point 52 h (*R*. *capsulatus*) and 164 h (*Synechocystis*) of the respective cultivation period (cf. A), and are representative for replicate data obtained from at least three independent cultivations. Typical pigment absorption is indicated. All values were normalized to OD_660nm_ (*R*. *capsulatus*) and OD_750nm_ (*Synechocystis*), respectively. BChl, bacteriochlorophyll *a*; Car, carotenoids; Chl, chlorophyll *a*; PC, phycocyanin. **(C)** Carotenoid content of *R*. *capsulatus* (upper panel) and *Synechocystis* (lower panel). Normalized values at 500 nm were plotted as a measure of carotenoid content in the host cells. *R*. *capsulatus* was analyzed at time points 24 h and 52 h during the cultivation. Pigments of *Synechocystis* were measured before and after cobalt-induction at time points 48 h and 164 h, respectively. Data represent mean results from three independent experiments, as well as the respective standard deviations. EVC, empty vector control; WT, wild type and Δ*shc* both harbor the empty vector.

Bacterial growth was monitored during expression experiments to investigate potential effects of triterpene formation on cell viability (**Fig 3A**). Since all strains reached comparable cell densities during the respective cultivations, cell growth was apparently unimpaired by heterologous gene expression and triterpene biosynthesis.

In addition, photopigment accumulation was analyzed in strains with precursor modules by recording whole cell absorbance spectra. Both hosts exhibited typical absorbance spectra after cultivation with expected maxima of cellular pigments including bacteriochlorophyll *a* and spheroidenone for *R*. *capsulatus* and chlorophyll *a*, carotenoids and phycobilins for *Synechocystis*, indicating that overall pigment synthesis is not affected by transferring the chosen plant triterpene pathways (**Fig 3B**). To analyze whether triterpene precursor formation results in a decrease of carotenoid (i.e. tetraterpene) formation via FPP depletion, carotenoid-related absorption at λ = 500 was specifically investigated (**Fig 3C**). Carotenoid-specific absorption remained unchanged in comparison to the control strains suggesting that the introduction of heterologous plant triterpene pathways were supplied by the cellular isoprene pool without interference with native tetraterpene biosynthesis.

## Discussion

In this work, we have demonstrated the applicability of the two phototrophic bacteria *R*. *capsulatus* SB1003 and *Synechocystis* sp. PCC 6803 as promising new prokaryotic hosts for the heterologous biosynthesis of diverse cyclic plant triterpene scaffolds.

Basically, the functional heterologous expression of triterpene biosynthesis enzymes requires suitable expression hosts that offer: i) a compatible metabolic background providing the isoprene-derived substrate molecules in high amounts; ii) an appropriate cellular environment including sufficient membrane space, supporting the activity of enzymes, such as OSCs, that favor hydrophobic environments [63–65] as well as storage of hydrophobic intermediates and products; and iii) a broad tolerance toward heterologous biosynthetic products. The heterologous expression of plant triterpene pathway genes in microbial host systems is hitherto almost exclusively analyzed in yeast cells. Only recently, also *E*. *coli* has been used as the first bacterial strain for functional expression of an OSC from plants leading to the formation of dammarenediol-II [19]. In this case, however, 2,3-oxidosqualene synthesis further required heterologous co-expression of an NADPH-cytochrome P450 reductase (CPR) in order to efficiently transfer electrons to the employed squalene epoxidase from *Methylococcus capsulatus*. In contrast, Banta and co-workers recently described an approach using the SQE from *Methylomicrobium alcaliphilum* to produce 2,3-oxidosqualene as precursor for cyclic triterpenoid synthesis likewise in *E*. *coli*, but without CPR co-expression [66]. In this context, our data clearly demonstrate that *R*. *capsulatus* and *Synechocystis* strains expressing plant SQE effectively convert squalene without coexpressing auxiliary reductases. Therefore, the phototrophic host cells likely provide native reductases that efficiently support SQE activity. However, while squalene quantitatively dissipated upon expression of SQE1, 2,3-oxidosqualene did not accumulate to equal amounts, with an especially pronounced decline in product levels in *Rhodobacter* (~30-fold) as compared to *Synechocystis* (~5-fold), suggesting either a further conversion by so far unknown enzymes or its export from the cells or a lower stability compared to squalene.

The expression of four different OSC enzymes yielded very different results regarding observed products in the two employed hosts. As for the expression of CAS1, both strains showed effective conversion of 2,3-oxidosqualene to cycloartenol as sole cyclization product, supporting previous reports on yeast strains expressing cycloartenol synthase homologs from different plant species [39,67]. In contrast, LUP1 expression led to formation of lupeol as well as the triterpenoid product lupX with observed m/z argueing for a further oxidized cyclization product in both strains. Previous studies on LUP1 activity in yeast cells suggested the synthesis of a variety of different cyclic products, including 3,20-lupanediol [40,61,62], which would match the here proposed structure of lupX. Furthermore, Krokida and co-workers reported that a closely related OSC from *Lotus japonicus*, AMY2, converts 2,3-oxidosqualene to both β-amyrin and dihydro-lupeol, without lupeol as intermediate when expressed in *Nicotiana benthamiana* [68]. In this host, the dihydro-lupeol product was further oxidized to 20-hydroxy-lupeol and 20-hydroxy-betulinic acid by the intrinsic cytochrome P450 (CYP) enzyme CYP71D353. However, when expressed in yeast cells, AMY2 was shown to produce β-amyrin and lupeol [69]. The discrepancies of catalytic products between diverging host systems thus indicates a striking influence of the cellular environment on the nature of the cyclization product(s). In *R*. *capsulatus* and *Synechocystis*, LUP1-mediated formation of oxidized lupeol-derivatives appears to be favored, potentially supported by native, yet undefined CYP acitvities. Likewise, MRN1 expression in *Synechocystis* leads to the formation of marnerol and–intriguingly–putative hydroxymarnerol. Marnerol accumulation was previously likewise reported for *S*. *cerevisiae* strains expressing MRN1, which has been attributed to a spontaneous conversion of the primary product marneral [42,51]. Accumulation of hydroxymarnerol, as here also observed in MRN1 expressing yeast strains (cf. **Fig 2D**), was previously shown in roots of *A*. *thaliana* as a product of the cytochrome P450 type marneral oxidase (MRO) activity [51]. Therefore, the presence of promiscuous oxidative enzyme activity in *Synechocystis* cells is likely to explain this observation. Indeed, the high potential for native CYP activity in cyanobacterial cells has previously been reported and was further supported by functional studies [70–72]. Among them are CYPs of *Nostoc* sp. capable of oxygenating sesquiterpenes when heterologously expressed in *E*. *coli* [73]. For *Synechocystis*, so far, oxygenation of C_20_ retinoids by CYP120A1 (*slr0574*) was demonstrated *in vitro* [74]. Notably, no MRN1 cyclization product could be detected in *R*. *capsulatus*, further argueing for the reasonability of establishing a variety of microbial platforms for triterpenoid biosynthesis. Moreover, lacking activity of THAS1 in both strains corroborates that successful biosynthesis of cyclic plant triterpenes depends not only on the choice of bacterial host, but also on the specific catalytic properties of the chosen OSC enzyme. In this context, it is worth mentioning that prokaryotic systems appear as yet underrepresented in triterpene production—despite their otherwise thorough exploitation with regard to other terpene classes (recently reviewed in [75–77]). This factum, together with our findings may serve to define plant OSC enzymes as generally rather ‘difficult-to-express’ proteins in bacteria, again underlining the demand for increasing the diversity of expression systems.

As indicated by unimpaired cell growth during heterologous biosynthesis, our host systems represent promising candidates for robust triterpene production, despite previous reports on an antibacterial activity of compounds from this group [78]. Possibly, the enlarged membrane space especially enables photosynthetic bacteria to store certain amounts of terpene precursors and products without significant interference with cellular functions. The two hosts showed rather different production profiles (**Table 2**). While *R*. *capsulatus* features faster growth and thus shorter production times, *Synechocystis* was capable of accumulating more triterpene products. However, the product levels of 2,3-oxidosqualene and cycloartenol obtained in both host strains as yet are lower than those previously described for 2,3-oxidosqualene and dammarenediol-II in *E*. *coli* [19]. Notably, in that study, genes of the FPP building MEP pathway were heterologously overexpressed. Employment of this strategy, typically co-expressing rate-limiting enzymes of the MEP pathway, and/or introducing the entire mevalonate (MVA) pathway has proven highly effective in enhancing bacterial terpene production. In *Rhodobacter*, such optimization has led to the successful production of terpene compounds to substantial levels [20] as for example Coenzyme Q_10_, which comprises a C_10_ isoprene side chain (138 mg L^-1^ [79]), the C_15_ sesquiterpene valencene (352 mg L^-1^ [33]), and non-cyclic C_30+_ triterpene botryococcene (110 mg L^-1^ [36]). As for cyanobacteria, similar efforts were successful for establishing significant yields [72] of e.g. C_5_ isoprene in *Synechococcus elongatus* (1.26 g L^-1^ [28]) and C_10_ β-phellandrene in *Synechocystis* (10 mg gDCW^-1^ [80]). In line with the here employed strategy for *R*. *capsulatus*, overexpression of squalene synthase might further increase triterpene yields in *Synechocystis*, where the precursor’s accumulation currently relies on disruption of native hopanoid biosynthesis. Accordingly, Choi and colleagues presented elevated yields of squalene in *S*. *elongatus* by combining heterologous SQS expression with MEP pathway optimization [27]. In both hosts, down-regulation of intrinsic carotenoid biosynthesis may represent an attractive strategy to increase the available pool of isoprene building blocks for heterologous terpene production. Recently, Wang and co-workers moreover demonstrated the importance of the terpene synthase step, reporting a 100-fold increase in monoterpene productivity in *S*. *elongatus* by enhancing the product sink, which was contrasted with rather marginal improvements by upstream pathway engineering [81]. In addition to metabolite flow into triterpene biosynthetic routes, we identified 2,3-oxidosqualene availability for the pathway as one limiting aspect for product yields–especially in *R*. *capsulatus*. Potentially, implementation of downstream OSC enzymes with improved activity for fast 2,3-oxidosqualene conversion, together with strain and process optimization might further increase yields of cyclic triterpene products in the future. In summary, the unique characteristics of photosynthetic bacteria, together with metabolic engineering approaches, may lead to production strains which may be able to produce high levels of triterpenoids from CO_2_.

Besides providing the chassis for efficient biotechnological production of secondary metabolites, microorganisms can support agricultural processes [82], particularly targeting crop yields and the nutritional value of staple foods [83]. In this context, plant growth promoting bacteria can be applied in order to optimize nutrient supply, phytohormone levels or resistance against biotic as well as abiotic stresses [23,84]. Using *Rhodobacter* or cyanobacterial species for co-cultivations, e.g. as part of artificial biofilms, has been shown to have the potential to improve e.g. yields and nutritional value of rice crops [22,85–87]. Furthermore, a basic experimental setup for *in vitro* co-cultivation of rice roots with our model chassis has been established in our lab (data not shown). Engineered biosynthesis of plant-protecting agents like glycosylated triterpenes (saponins) in these bacteria might further contribute to novel strategies in desease management, particularly targeting fungal pathogens. Such strategies further require the heterologous expression of additional product-decorating enzymes [17]. Importantly, the basic suitability of photosynthetic bacteria for functional expression of plant cytochrome P450 and glycosyltransferase enzymes was recently demonstrated using the example of dhurrin pathway reconstitution in *Synechocystis* 6803 [88]. As an overall future perspective, a vast chemical space of triterpene structures has come into reach, not only by availability of increasing numbers of plant OSC genes, but also by rational enzyme engineering approaches allowing for qualitatively altering product specificities. In this sense, it was recently demonstrated that substrate and product specificities of OSC enzymes can be altered by point mutations of a single amino acid residue close to the active site of the enzyme [89]. In summary, the variety of plant triterpenes harbor a huge potential for different biotechnological applications, particularly addressing the biomedical and agricultural sectors. The here presented host systems shall contribute to realize these applications.

## Supporting information

S1 Table List of primers used in this study. (PDF)

S2 Table Sequence table. Word document containing DNA sequences of synthetic operons in expression vectors.
(DOCX)

S3 Table Sum formulas and calculated m/z of selected triterpenes. (PDF)

S1 Fig Plasmid maps. Document containing plasmid maps of vectors for expression in *R*. *capsulatus* (pRhon5Hi-2, pRhon5Hi-2-SQS1, pRhon5Hi-2-SQS1-SQE1, and pRhon5Hi-2-LUP1-SQS1-SQE1) and of vectors for expression in *Synechocystis* (pVZ-spec, pVZ-PcoaT-SQE1, and pVZ-PcoaT-SQE1-LUP1).
(DOCX)

S2 Fig LC-MS data. Document containing MS spectra of all analyzed triterpenoids as detected in bacteria; MS/MS spectra of triterpenoids squalene, 2,3-oxidosqualene, cycloartenol, lupeol and lupX as detected in bacteria; LC-MS chromatrograms of extracts from THAS1-expressing bacteria; MS/MS spectra of marnerol/hydroxymarnerol as detected in *Synechocystis*.
(DOCX)

## References

[1] Wink M. Modes of Action of Herbal Medicines and Plant Secondary Metabolites. Medicines. 2015;2:251 doi: 10.3390/medicines2030251 28930211 10.3390/medicines2030251 PMC5456217

[2] Dutton A, Mattiacci L, Amadò R, Dorn S. A novel function of the triterpene squalene in a tritrophic system. J Chem Ecol. 2002;28:103–116. 11868668 10.1023/a:1013514903036

[3] Babiychuk E, Bouvier-Navé P, Compagnon V, Suzuki M, Muranaka T, Van Montagu M, et al Allelic mutant series reveal distinct functions for *Arabidopsis* cycloartenol synthase 1 in cell viability and plastid biogenesis. Proc Natl Acad Sci USA. 2008;105:3163–3168. doi: 10.1073/pnas.0712190105 18287026 10.1073/pnas.0712190105 PMC2268602

[4] Kemen AC, Honkanen S, Melton RE, Findlay KC, Mugford ST, Hayashi K, et al Investigation of triterpene synthesis and regulation in oats reveals a role for β-amyrin in determining root epidermal cell patterning. Proc Natl Acad Sci USA. 2014;111:8679–8684. doi: 10.1073/pnas.1401553111 24912185 10.1073/pnas.1401553111 PMC4060722

[5] Go YS, Lee SB, Kim HJ, Kim J, Park H-Y, Kim J-K, et al Identification of marneral synthase, which is critical for growth and development in *Arabidopsis*. Plant J. 2012;72:791–804. doi: 10.1111/j.1365-313X.2012.05120.x 22882494 10.1111/j.1365-313X.2012.05120.x

[6] Delis C, Krokida A, Georgiou S, Peña-Rodríguez LM, Kavroulakis N, Ioannou E, et al Role of lupeol synthase in *Lotus japonicus* nodule formation. New Phytol. 2011;189:335–346. doi: 10.1111/j.1469-8137.2010.03463.x 20868395 10.1111/j.1469-8137.2010.03463.x

[7] Augustin JM, Kuzina V, Andersen SB, Bak S. Molecular activities, biosynthesis and evolution of triterpenoid saponins. Phytochemistry. 2011;72:435–457. doi: 10.1016/j.phytochem.2011.01.015 21333312 10.1016/j.phytochem.2011.01.015

[8] Chen M, Zhong Z, Tan W, Wang S, Wang Y. Recent advances in nanoparticle formulation of oleanolic acid. Chin Med. 2011;6:20 doi: 10.1186/1749-8546-6-20 21619582 10.1186/1749-8546-6-20 PMC3123256

[9] Xi J, Tang HY, Zheng Y. Oral dosage forms of oleanolic acid and their pharmacokinetics. Chin J New Drugs. 2009;18:507–515.

[10] Yamaguchi H, Noshita T, Kidachi Y, Umetsu H, Hayashi M, Komiyama K, et al Isolation of ursolic acid from apple peels and its specific efficacy as a potent antitumor agent. J Health Sci. 2008;54:654–660.

[11] Řezanka T, Siristova L, Sigler K. Sterols and triterpenoids with antiviral activity. Antiinfect Agents Med Chem. 2009;8:193–210.

[12] Díaz AM, Abad MJ, Fernández L, Recuero C, Villaescusa L, Silván AM, et al *In vitro* anti-inflammatory activity of iridoids and triterpenoid compounds isolated from *Phillyrea latifolia* L. Biol Pharm Bull 2000;23:1307–1313. 11085357 10.1248/bpb.23.1307

[13] Szakiel A, Pączkowski C, Pensec F, Bertsch C. Fruit cuticular waxes as a source of biologically active triterpenoids. Phytochem Rev. 2012;11:263–284. doi: 10.1007/s11101-012-9241-9 23519009 10.1007/s11101-012-9241-9 PMC3601259

[14] Mannowetz N, Miller MR, Lishko PV. Regulation of the sperm calcium channel CatSper by endogenous steroids and plant triterpenoids. Proc Natl Acad Sci USA. 2017;114(22):5743–5748. doi: 10.1073/pnas.1700367114 28507119 10.1073/pnas.1700367114 PMC5465908

[15] Nakashima T, Inoue T, Oka A, Nishino T, Osumi T, Hata S. Cloning, expression, and characterization of cDNAs encoding *Arabidopsis thaliana* squalene synthase. Proc Natl Acad Sci USA. 1995;92:2328–2332. 7892265 10.1073/pnas.92.6.2328 PMC42476

[16] Laden BP, Tang Y, Porter TD. Cloning, heterologous expression, and enzymological characterization of human squalene monooxygenase. Arch Biochem Biophys. 2000;374:381–388. doi: 10.1006/abbi.1999.1629 10666321 10.1006/abbi.1999.1629

[17] Thimmappa R, Geisler K, Louveau T, O'Maille P, Osbourn A. Triterpene biosynthesis in plants. Annu Rev Plant Biol. 2014;65:225–257. doi: 10.1146/annurev-arplant-050312-120229 24498976 10.1146/annurev-arplant-050312-120229

[18] Dziggel C, Schäfer H, Wink M. Tools of pathway reconstruction and production of economically relevant plant secondary metabolites in recombinant microorganisms. Biotechnol J. 2017;12:1600145. 10.1002/biot.201600145 28009095

[19] Li D, Zhang Q, Zhou Z, Zhao F, Lu W. Heterologous biosynthesis of triterpenoid dammarenediol-II in engineered *Escherichia coli*. Biotechnol Lett. 2016;38:603–609. doi: 10.1007/s10529-015-2032-9 26739962 10.1007/s10529-015-2032-9

[20] Heck A, Drepper T. Engineering Photosynthetic α-Proteobacteria for Microbial Production of Recombinant Proteins and Terpenes *In* Hallenbeck P (ed), Modern Topics in the Phototrophic Prokaryotes (Springer International Publishing); 2017 pp. 395–425.

[21] Al-Haj L, Lui YT, Abed RM, Gomaa MA, Purton S. Cyanobacteria as Chassis for Industrial Biotechnology: Progress and Prospects. Life (Basel). 2016;6(4). pii:E42. 10.3390/life6040042 PMC5198077 27916886

[22] Gamal-Eldin H, Elbanna K. Field evidence for the potential of *Rhodobacter capsulatus* as biofertilizer for flooded rice. Curr Microbiol. 2011;62:391–395. doi: 10.1007/s00284-010-9719-x 20697715 10.1007/s00284-010-9719-x

[23] Singh S. A review on possible elicitor molecules of cyanobacteria: their role in improving plant growth and providing tolerance against biotic or abiotic stress. J Appl Microbiol. 2014;117:1221–1244. doi: 10.1111/jam.12612 25069397 10.1111/jam.12612

[24] Armstrong GA, Schmidt A, Sandmann G, Hearst JE. Genetic and biochemical characterization of carotenoid biosynthesis mutants of *Rhodobacter capsulatus*. J Biol Chem. 1990;265:8329–8338. 2159477

[25] Armstrong GA. Eubacteria show their true colors: genetics of carotenoid pigment biosynthesis from microbes to plants. J Bacteriol. 1994;176:4795–4802. 8050991 10.1128/jb.176.16.4795-4802.1994 PMC196312

[26] Zhang L, Selão TT, Selstam E, Norling B. Subcellular localization of carotenoid biosynthesis in *Synechocystis* sp. PCC 6803. PLoS One. 2015;10:e0130904 doi: 10.1371/journal.pone.0130904 26083372 10.1371/journal.pone.0130904 PMC4470828

[27] Choi SY, Lee HJ, Choi J, Kim J, Sim SJ, Um Y, et al Photosynthetic conversion of CO_2_ to farnesyl diphosphate-derived phytochemicals (amorpha-4,11-diene and squalene) by engineered cyanobacteria. Biotechnol Biofuels. 2016;9:202 doi: 10.1186/s13068-016-0617-8 27688805 10.1186/s13068-016-0617-8 PMC5034544

[28] Gao X, Gao F, Liu D, Zhang H, Nie X, Yang C. Engineering the methylerythritol phosphate pathway in cyanobacteria for photosynthetic isoprene production from CO_2_. Energy & Environ Sci. 2016;9:1400–1411.

[29] Englund E, Andersen-Ranberg J, Miao R, Hamberger B, Lindberg P. Metabolic engineering of *Synechocystis* sp. PCC 6803 for production of the plant diterpenoid manoyl oxide. ACS Synth Biol. 2015;4:1270–1278. doi: 10.1021/acssynbio.5b00070 26133196 10.1021/acssynbio.5b00070 PMC4685428

[30] Davies FK, Work VH, Beliaev AS, Posewitz MC. Engineering limonene and bisabolene production in wild type and a glycogen-deficient mutant of *Synechococcus* sp. PCC 7002. Front Bioeng Biotechnol. 2014;2:21 doi: 10.3389/fbioe.2014.00021 25152894 10.3389/fbioe.2014.00021 PMC4126464

[31] Bentley FK, Zurbriggen A, Melis A. Heterologous expression of the mevalonic acid pathway in cyanobacteria enhances endogenous carbon partitioning to isoprene. Mol Plant. 2014;7:71–86. doi: 10.1093/mp/sst134 24157609 10.1093/mp/sst134

[32] Bentley FK, García-Cerdán JG, Chen H-C, Melis A. Paradigm of monoterpene (*β*-phellandrene) hydrocarbons production via photosynthesis in cyanobacteria. Bioenerg Res. 2013;6:917–929.

[33] Beekwilder J, van Houwelingen A, Cankar K, van Dijk AD, de Jong RM, Stoopen G, et al Valencene synthase from the heartwood of Nootka cypress (*Callitropsis nootkatensis*) for biotechnological production of valencene. Plant Biotechnol J. 2014;12:174–182. doi: 10.1111/pbi.12124 24112147 10.1111/pbi.12124

[34] Loeschcke A, Markert A, Wilhelm S, Wirtz A, Rosenau F, Jaeger KE, et al TREX: a universal tool for the transfer and expression of biosynthetic pathways in bacteria. ACS Synth Biol. 2013;2:22–33. doi: 10.1021/sb3000657 23656323 10.1021/sb3000657

[35] Pattanaik B, Lindberg P. Terpenoids and their biosynthesis in cyanobacteria. Life (Basel). 2015;5:269–293. 25615610 10.3390/life5010269 PMC4390852

[36] Khan NE, Nybo SE, Chappell J, Curtis WR. Triterpene hydrocarbon production engineered into a metabolically versatile host-*Rhodobacter capsulatus*. Biotechnol Bioeng. 2015;112:1523–1532. doi: 10.1002/bit.25573 25728701 10.1002/bit.25573

[37] Englund E, Pattanaik B, Ubhayasekera SJ, Stensjö K, Bergquist J, Lindberg P. Production of squalene in *Synechocystis* sp. PCC 6803. PLoS One. 2014;9:e90270 doi: 10.1371/journal.pone.0090270 24625633 10.1371/journal.pone.0090270 PMC3953072

[38] Rasbery JM, Shan H, LeClair RJ, Norman M, Matsuda SP, Bartel B. *Arabidopsis thaliana* squalene epoxidase 1 is essential for root and seed development. J Biol Chem. 2007;282:17002–17013. doi: 10.1074/jbc.M611831200 17426032 10.1074/jbc.M611831200

[39] Corey EJ, Matsuda SP, Bartel B. Isolation of an *Arabidopsis thaliana* gene encoding cycloartenol synthase by functional expression in a yeast mutant lacking lanosterol synthase by the use of a chromatographic screen. Proc Natl Acad Sci USA. 1993;90:11628–11632. 7505443 10.1073/pnas.90.24.11628 PMC48037

[40] Herrera JB, Bartel B, Wilson WK, Matsuda SP. Cloning and characterization of the *Arabidopsis thaliana* lupeol synthase gene. Phytochemistry. 1998;49:1905–1911. 9883589 10.1016/s0031-9422(98)00366-5

[41] Fazio GC, Xu R, Matsuda SP. Genome mining to identify new plant triterpenoids. J Am Chem Soc. 2004;126:5678–5679. doi: 10.1021/ja0318784 15125655 10.1021/ja0318784

[42] Xiong Q, Wilson WK, Matsuda SP. An *Arabidopsis* oxidosqualene cyclase catalyzes iridal skeleton formation by Grob fragmentation. J Am Chem Soc. 2006;45:1285–1288. 10.1002/anie.200503420 16425307

[43] Hanahan D. Studies on transformation of *Escherichia coli* with plasmids. J Mol Biol. 1983;166:557–580. 6345791 10.1016/s0022-2836(83)80284-8

[44] Elhai J, Wolk CP. Conjugal transfer of DNA to cyanobacteria. Methods Enzymol. 1988;167:747–754. 3148842 10.1016/0076-6879(88)67086-8

[45] Simon R, Priefer U, Pühler A. A broad host range mobilization system for *in vivo* genetic engineering: transposon mutagenesis in gram negative bacteria. Nat Biotechnol. 1983;1: 784–791.

[46] Strnad H, Lapidus A, Paces J, Ulbrich P, Vlcek C, Paces V, et al Complete genome sequence of the photosynthetic purple nonsulfur bacterium *Rhodobacter capsulatus* SB 1003. J Bacteriol. 2010;192:3545–3546. doi: 10.1128/JB.00366-10 20418398 10.1128/JB.00366-10 PMC2897665

[47] Klipp W, Masepohl B, Pühler A. Identification and mapping of nitrogen fixation genes of *Rhodobacter capsulatus*: duplication of a *nifA-nifB* region. J Bacteriol. 1988;170:693–699. 2828320 10.1128/jb.170.2.693-699.1988 PMC210710

[48] Weaver PF, Wall JD, Gest H. Characterization of *Rhodopseudomonas capsulata*. Arch Microbiol. 1975;105:207–216. 1103769 10.1007/BF00447139

[49] Rippka R, Deruelles J, Waterbury JB, Herdman M, Stanier RY. Generic assignments, strain histories and properties of pure cultures of cyanobacteria. J Gen Microbiol. 1979;111:1–61.

[50] Gollub EG, Liu K-P, Dayan J, Adlersberg M, Sprinson DB. Yeast mutants deficient in heme biosynthesis and a heme mutant additionally blocked in cyclization of 2,3-oxidosqualene. J Biol Chem. 1977;252:2846–2854. 323256

[51] Field B, Fiston-Lavier AS, Kemen A, Geisler K, Quesneville H, Osbourn AE. Formation of plant metabolic gene clusters within dynamic chromosomal regions. Proc Natl Acad Sci USA. 2011;108:16116–16121. doi: 10.1073/pnas.1109273108 21876149 10.1073/pnas.1109273108 PMC3179108

[52] Kushiro T, Shibuya M, Ebizuka Y. β-amyrin synthase-cloning of oxidosqualene cyclase that catalyzes the formation of the most popular triterpene among higher plants. Eur J Biochem. 1998;256:238–244. 9746369 10.1046/j.1432-1327.1998.2560238.x

[53] Katzke N, Arvani S, Bergmann R, Circolone F, Markert A, Svensson V, et al A novel T7 RNA polymerase dependent expression system for high-level protein production in the phototrophic bacterium *Rhodobacter capsulatus*. Protein Expr Purif. 2010;69:137–146. doi: 10.1016/j.pep.2009.08.008 19706327 10.1016/j.pep.2009.08.008

[54] Mitschke J, Georg J, Scholz I, Sharma CM, Dienst D, Bantscheff J, et al An experimentally anchored map of transcriptional start sites in the model cyanobacterium *Synechocystis* sp. PCC6803. Proc Natl Acad Sci USA. 2011;108:2124–2129. doi: 10.1073/pnas.1015154108 21245330 10.1073/pnas.1015154108 PMC3033270

[55] Zinchenko VV, Piven IV, Melnik VA, Shestakov SV. Vectors for the complementation analysis of cyanobacterial mutants. Russ J Genet. 1999;35:228–232.

[56] Beyer HM, Gonschorek P, Samodelov SL, Meier M, Weber W, Zurbriggen MD. AQUA cloning: a versatile and simple enzyme-free cloning approach. PLoS One. 2015;10:e0137652 doi: 10.1371/journal.pone.0137652 26360249 10.1371/journal.pone.0137652 PMC4567319

[57] Busquets A, Keim V, Closa M, del Arco A, Boronat A, Arró M, et al *Arabidopsis thaliana* contains a single gene encoding squalene synthase. Plant Mol Biol. 2008;67:25–36. doi: 10.1007/s11103-008-9299-3 18236008 10.1007/s11103-008-9299-3

[58] Takaichi S, Mochimaru M. Carotenoids and carotenogenesis in cyanobacteria: unique ketocarotenoids and carotenoid glycosides. Cell Mol Life Sci. 2007;64:2607–2619. doi: 10.1007/s00018-007-7190-z 17643187 10.1007/s00018-007-7190-z PMC11136355

[59] Peca L, Kós PB, Vass I. Characterization of the activity of heavy metal-responsive promoters in the cyanobacterium *Synechocystis* PCC 6803. Acta Biol Hung. 2007;58 Suppl:11–22. 10.1556/ABiol.58.2007.Suppl.2 18297791

[60] Georg J, Dienst D, Schürgers N, Wallner T, Kopp D, Stazic D, et al The small regulatory RNA SyR1/PsrR1 controls photosynthetic functions in cyanobacteria. Plant Cell. 2014;26:3661–3679. doi: 10.1105/tpc.114.129767 25248550 10.1105/tpc.114.129767 PMC4213160

[61] Segura MJ, Meyer MM, Matsuda SP. *Arabidopsis thaliana* LUP1 converts oxidosqualene to multiple triterpene alcohols and a triterpene diol. Org Lett. 2000;2:2257–2259. 10930257 10.1021/ol006016b

[62] Kushiro T, Hoshino M, Tsutsumi T, Kawai K, Shiro M, Shibuya M, et al Stereochemical course in water addition during LUP1-catalyzed triterpene cyclization. Org Lett. 2006;8:5589–5592. doi: 10.1021/ol062310d 17107079 10.1021/ol062310d

[63] Liang Y-L, Zhao S-J, Xu L-X, Zhang X-Y. Heterologous expression of dammarenediol synthase gene in an engineered *Saccharomyces cerevisiae*. Lett Appl Microbiol. 2012;55:323–329. doi: 10.1111/j.1472-765X.2012.03295.x 22897704 10.1111/j.1472-765X.2012.03295.x

[64] Milla P, Athenstaedt K, Viola F, Oliaro-Bosso S, Kohlwein SD, Daum G, et al Yeast oxidosqualene cyclase (Erg7p) is a major component of lipid particles. J Biol Chem. 2002;277:2406–2412. doi: 10.1074/jbc.M104195200 11706015 10.1074/jbc.M104195200

[65] Milla P, Viola F, Oliaro-Bosso S, Rocco F, Cattel L, Joubert BM, et al Subcellular localization of oxidosqualene cyclases from *Arabidopsis thaliana*, *Trypanosoma cruzi*, and *Pneumocystis carinii* expressed in yeast. Lipids. 2002;37:1171–1176. 12617471 10.1007/s11745-002-1017-9

[66] Banta AB, Wei JH, Gill CC, Giner J-L, Welander PV. Synthesis of arborane triterpenols by a bacterial oxidosqualene cyclase. Proc Natl Acad Sci USA. 2016;114:245–250. doi: 10.1073/pnas.1617231114 28028245 10.1073/pnas.1617231114 PMC5240688

[67] Hayashi H, Hiraoka N, Ikeshiro Y, Kushiro T, Morita M, Shibuya M, et al Molecular cloning and characterization of a cDNA for *Glycyrrhiza glabra* cycloartenol synthase. Biol Pharm Bull. 2000;23:231–234. 10706391 10.1248/bpb.23.231

[68] Krokida A, Delis C, Geisler K, Garagounis C, Tsikou D, Peña-Rodríguez LM, et al A metabolic gene cluster in *Lotus japonicus* discloses novel enzyme functions and products in triterpene biosynthesis. New Phytol. 2013;200:675–690. doi: 10.1111/nph.12414 23909862 10.1111/nph.12414

[69] Iturbe-Ormaetxe I, Haralampidis K, Papadopoulou K, Osbourn AE. Molecular cloning and characterization of triterpene synthases from *Medicago truncatula* and *Lotus japonicus*. Plant Mol Biol. 2003;51:731–743. 12683345 10.1023/a:1022519709298

[70] Robert FO, Pandhal J, Wright PC. Exploiting cyanobacterial P450 pathways. Curr Opin Microbiol. 2010;13:301–306. doi: 10.1016/j.mib.2010.02.007 20299274 10.1016/j.mib.2010.02.007

[71] Ke N, Baudry J, Makris TM, Schuler MA, Sligar SG. A retinoic acid binding cytochrome P450: CYP120A1 from *Synechocystis* sp. PCC 6803. Arch Biochem Biophys. 2005;436:110–120. doi: 10.1016/j.abb.2005.01.011 15752715 10.1016/j.abb.2005.01.011

[72] Xue Y, He Q. Cyanobacteria as cell factories to produce plant secondary metabolites. Front Bioeng Biotechnol. 2015;3:57 doi: 10.3389/fbioe.2015.00057 25973419 10.3389/fbioe.2015.00057 PMC4412135

[73] Agger SA, Lopez-Gallego F, Hoye TR, Schmidt-Dannert C. Identification of sesquiterpene synthases from *Nostoc punctiforme* PCC 73102 and *Nostoc* sp. strain PCC 7120. J Bacteriol. 2008;190:6084–6096. doi: 10.1128/JB.00759-08 18658271 10.1128/JB.00759-08 PMC2546793

[74] Alder A, Bigler P, Werck-Reichhart D, Al-Babili S. *In vitro* characterization of *Synechocystis* CYP120A1 revealed the first nonanimal retinoic acid hydroxylase. FEBS J. 2009;276:5416–5431. doi: 10.1111/j.1742-4658.2009.07224.x 19703230 10.1111/j.1742-4658.2009.07224.x

[75] Jongedijk E, Cankar K, Buchhaupt M, Schrader J, Bouwmeester H, Beekwilder J. Biotechnological production of limonene in microorganisms. Appl Microbiol Biotechnol. 2016;100:2927–2938. doi: 10.1007/s00253-016-7337-7 26915992 10.1007/s00253-016-7337-7 PMC4786606

[76] Xie M, Wang W, Zhang W, Chen L, Lu X. Versatility of hydrocarbon production in cyanobacteria. Appl Microbiol Biotechnol. 2017;101:905–919. doi: 10.1007/s00253-016-8064-9 28032195 10.1007/s00253-016-8064-9

[77] Majdi M, Ashengroph M, Abdollahi MR. Sesquiterpene lactone engineering in microbial and plant platforms: parthenolide and artemisinin as case studies. Appl Microbiol Biotechnol. 2016;100:1041–1059. doi: 10.1007/s00253-015-7128-6 26567019 10.1007/s00253-015-7128-6

[78] Chung PY, Navaratnam P, Chung LY. Synergistic antimicrobial activity between pentacyclic triterpenoids and antibiotics against *Staphylococcus aureus* strains. Ann Clin Microbiol Antimicrob. 2011;10:25 doi: 10.1186/1476-0711-10-25 21658242 10.1186/1476-0711-10-25 PMC3127748

[79] Lu W, Ye L, Xu H, Xie W, Gu J, Yu H. Enhanced production of coenzyme Q10 by self-regulating the engineered MEP pathway in *Rhodobacter sphaeroides*. Biotechnol Bioeng. 2014;111:761–769. doi: 10.1002/bit.25130 24122603 10.1002/bit.25130

[80] Formighieri C, Melis A. Sustainable heterologous production of terpene hydrocarbons in cyanobacteria. Photosynth Res. 2016;130: 123–135. doi: 10.1007/s11120-016-0233-2 26895437 10.1007/s11120-016-0233-2

[81] Wang X, Liu W, Xin C, Zheng Y, Cheng Y, Sun S, et al Enhanced limonene production in cyanobacteria reveals photosynthesis limitations. Proc Natl Acad Sci USA. 2016;113:14225–14230. doi: 10.1073/pnas.1613340113 27911807 10.1073/pnas.1613340113 PMC5167140

[82] Moshelion M, Altman A. Current challenges and future perspectives of plant and agricultural biotechnology. Trends Biotechnol. 2015;33:337–342. doi: 10.1016/j.tibtech.2015.03.001 25842169 10.1016/j.tibtech.2015.03.001

[83] McKersie B. Planning for food security in a changing climate. J Exp Bot. 2015;66:3435–3450. doi: 10.1093/jxb/eru547 25614663 10.1093/jxb/eru547

[84] Lugtenberg B, Kamilova F. Plant-growth-promoting rhizobacteria. Annu Rev Microbiol. 2009;63:541–556. doi: 10.1146/annurev.micro.62.081307.162918 19575558 10.1146/annurev.micro.62.081307.162918

[85] Priya H, Prasanna R, Ramakrishnan B, Bidyarani N, Babu S, Thapa S, et al Influence of cyanobacterial inoculation on the culturable microbiome and growth of rice. Microbiol Res. 2015;171:78–89. doi: 10.1016/j.micres.2014.12.011 25644956 10.1016/j.micres.2014.12.011

[86] Adak A, Prasanna R, Babu S, Bidyarani N, Verma S, Pal M, et al Micronutrient enrichment mediated by plant-microbe interactions and rice cultivation practices. J Plant Nutr. 2016;39:1216–1232.

[87] Prasanna R, Adak A, Verma S, Bidyarani N, Babu S, Pal M, et al Cyanobacterial inoculation in rice grown under flooded and SRI modes of cultivation elicits differential effects on plant growth and nutrient dynamics. Ecol Eng. 2015;84:532–541.

[88] Wlodarczyk A, Gnanasekaran T, Nielsen AZ, Zulu NN, Mellor SB, Luckner M, et al Metabolic engineering of light-driven cytochrome P450 dependent pathways into *Synechocystis* sp. PCC 6803. Metab Eng. 2016;33:1–11. doi: 10.1016/j.ymben.2015.10.009 26548317 10.1016/j.ymben.2015.10.009

[89] Salmon M, Thimmappa RB, Minto RE, Melton RE, Hughes RK, O'Maille PE, et al A conserved amino acid residue critical for product and substrate specificity in plant triterpene synthases. Proc Natl Acad Sci USA. 2016;113:E4407–4414. doi: 10.1073/pnas.1605509113 27412861 10.1073/pnas.1605509113 PMC4968742

