# Supplementary material for: The photosynthetic bacteria Rhodobacter capsulatus and Synechocystis sp. PCC 6803 as new hosts for cyclic plant triterpene biosynthesis
Source: PLoS One. 2017 Dec 27;12(12):e0189816. doi: 10.1371/journal.pone.0189816 (PMC5744966; doi:10.1371/journal.pone.0189816)
Supplement: S1 Table — (PDF) [file pone.0189816.s001.pdf]

The photosynthetic bacteria *Rhodobacter capsulatus* and *Synechocystis* sp. PCC 6803 as new hosts for cyclic plant triterpene biosynthesis

Anita Loeschcke, Dennis Dienst Dienst, Vera Wewer, Jennifer Hage-Hülsmann, Maximilian Dietsch, Sarah Kranz-Finger, Vanessa Hüren, Sabine Metzger, Vlada B. Urlacher, Tamara Gigolashvili, Stanislav Kopriva, Ilka M. Axmann, Thomas Drepper, Karl-Erich Jaeger

S1 Table. List of primers used in this study.

Primer ID	Sequence	Target	Use
#252_NheI_SQE_fw	ACACATGGCTAGCCAGCTGTGGAACTGG	*A.t. SQE1*	PCR
#253_terT7_SpeI_rev	ACACACTAGTCGCCAATCCGGATATAGTTC	T7 Terminator	PCR
#510_LUP1-Rc for	ATATACATATGTGGAAACTGAAGATCGGGAAGGG	*A.t. LUP1*	PCR
#511_LUP1-Rc rev	ATATACATATGTCAATTCCTTGTCAAAGGCTTAATTGACG ATGAAGACGACC	*A.t. LUP1*	PCR
#512_CAS1-Rc for	ATATACATATGTGGAAACTGAAAATCGCGGAAGG	*A.t. CAS1*	PCR
#513_CAS1-Rc rev	ATATACATATGTCAATTCCTTGTCAAAGGCTAATTCGCCC TGCTGCAGCAG	*A.t. CAS1*	PCR
#514_MRN1-Rc for	ATATACATATGTGGCGCCTGCGCATCGGCG	*A.t. MRN1*	PCR
#515_MRN1-Rc rev	ATATACATATGTCAATTCCTTGTCAAAGGCTTAGCTCACC AGCAGGCG	*A.t. MRN1*	PCR
#516_THAS1-Rc for	ATATACATATGGGCGCCTGCGCACCG	*A.t. THAS1*	PCR
#517_THAS1-Rc rev	ATATACATATGTCAATTCCTTGTCAAAGGC	*A.t. THAS1*	PCR
#524_Aq_LUP1_SQE_fw	TAAAAGAGGAGAAATACTAGATGTGGAAACTGAAG	*A.t. LUP1*/*SQE1*	AQUA
#525_Aq_LUP1_pJET_rev	TCGAGTGCGGCCGCAAGCTTTTAATTGACGATGAAGACG ACC	*A.t. LUP1*/pJET	AQUA
#526_Aq_CAS1_SQE_fw	TAAAAGAGGAGAAATACTAGATGTGGAAACTGAAAATCG CGGAAGGCGGCA	*A.t. CAS1*/*SQE1*	AQUA
#527_Aq_CAS1_pJET_rev	TCGAGTGCGGCCGCAAGCTTTTATTCGCCCTGCTGCAGC AG	*A.t. CAS1*/pJET	AQUA
#528_Aq_PcoaT_fw	AGCAAGATAGGTCAATGCATGAATTCGCGGCC	Syn6803 PcoaT	AQUA
#529_Aq_SQE_CAS1_rev	GCGATTTTCAGTTTCCACATCTAGTATTTCTCCTCTTTTAC GCG	*A.t. SQE1*/*CAS1*	AQUA
#530_Aq_SQE_LUP1_rev	CCGATCTTCAGTTTCCACATCTAGTATTTCTCCTCTTTTAC G	*A.t. SQE1*/*LUP1*	AQUA
#531_Aq_pJET_back_CAS1_fw	TGCTGCAGCAGGGCGAATAAAAGCTTGCGGCCGCAC	pJET/*A.t. CAS1*	AQUA
#532_Aq_pJET_back_coaT_rev	CTAGAAGCGGCCGCGAATTCATGCATTGACCT	pJET/Syn P*coaT*	AQUA
#533_Aq_pJET_back_LUP1_fw	TCGTCTTCATCGTCAATTAAAAGCTTGCGGCCGCAC	pJET/*A.t. LUP1*	AQUA
