# Supplementary material for: The photosynthetic bacteria Rhodobacter capsulatus and Synechocystis sp. PCC 6803 as new hosts for cyclic plant triterpene biosynthesis
Source: PLoS One. 2017 Dec 27;12(12):e0189816. doi: 10.1371/journal.pone.0189816 (PMC5744966; doi:10.1371/journal.pone.0189816)
Supplement: S2 Table — Word document containing DNA sequences of synthetic operons in expression vectors. (DOCX) [file pone.0189816.s002.docx]

**The photosynthetic bacteria *Rhodobacter capsulatus* and *Synechocystis* sp. PCC 6803 as new hosts for cyclic plant triterpene biosynthesis**

Anita Loeschcke, Dennis Dienst Dienst, Vera Wewer, Jennifer Hage-Hülsmann, Maximilian Dietsch, Sarah Kranz-Finger, Vanessa Hüren, Sabine Metzger, Vlada B. Urlacher, Tamara Gigolashvili, Stanislav Kopriva, Ilka M. Axmann, Thomas Drepper, Karl-Erich Jaeger

**S2 Table. Sequence Table.**

**content**

**page**

**Inserts in pRhon5Hi-2-based vectors for expression in *R. capsulatus***

SQS1 2

SQS1-SQE1 3

CAS1-SQS1-SQE1 5

LUP1-SQS1-SQE1 8

THAS1-SQS1-SQE1 11

MRN1-SQS1-SQE1 14

**Inserts in pVZ-based vectors for expression in *Synechocystis***

PcoaT-SQE1 17

PcoaT-SQE1-CAS1 19

PcoaT-SQE1-LUP1 22

PcoaT-SQE1-THAS1 25

PcoaT-SQE1-MRN1 28

pVZ-spec 31 **Inserts in template vectors for cloning**

H6-THAS1-SQS1-SQE1 – template for THAS1-nifK RBS 36

SQS1-SQE1-MRN1 – template for SQE1-MRN1 39

SQS1-SQE1-THAS1 – template for SQE1-THAS1 42

**Inserts in pRhon5Hi-2-based vectors for expression in *R. capsulatus***

**pRhon5Hi-2-SQS1**

LOCUS SQS1 1439 bp DNA linear SYN 24-OCT-2017

KEYWORDS pRhon5Hi-2-SQS1

FEATURES Location/Qualifiers

regulatory 29..34

/regulatory_class=ribosome_binding_site

/gene="pRhotHi-2 RBS"

/product="RBS of vector pRhotHi-2"

CDS 43..1275

/gene="SQS1"

/product="A. thaliana squalene synthase SQS1"

/translation="MGSLGTMLRYPDDIYPLLKMKRAIEKAEKQIPPEPHWGFCYSML

HKVSRSFSLVIQQLNTELRNAVCVFYLVLRALDTVEDDTSIPTDEKVPILIAFHRHIY

DTDWHYSCGTKEYKILMDQFHHVSAAFLELEKGYQEAIEEITRRMGAGMAKFICQEVE

TVDDYDEYCHYVAGLVGLGLSKLFLAAGSEVLTPDWEAISNSMGLFLQKTNIIRDYLE

DINEIPKSRMFWPREIWGKYADKLEDLKYEENTNKSVQCLNEMVTNALMHIEDCLKYM

VSLRDPSIFRFCAIPQIMAIGTLALCYNNEQVFRGVVKLRRGLTAKVIDRTKTMADVY

GAFYDFSCMLKTKVDKNDPNASKTLNRLEAVQKLCRDAGVLQNRKSYVNDKGQPNSVF

IIMVVILLAIVFAYLRAN"

regulatory 1392..1439

/regulatory_class=terminator

/gene="T7 terminator"

/product="bacteriophage T7 transcription terminator"

ORIGIN

1 tctagaaata attttgttta actttaagaa ggagatatac atatgggcag cctgggcacc

61 atgctgcgct atccggatga tatctatccg ctgctgaaaa tgaaacgcgc gatcgaaaaa

121 gcggaaaaac agatcccgcc ggaaccgcat tggggcttct gctatagcat gctgcataaa

181 gtgagccgca gcttcagcct ggtgatccag cagctgaaca ccgaactgcg caacgcggtg

241 tgcgtgttct atctggtgct gcgcgcgctg gataccgtgg aagatgatac cagcatcccg

301 accgatgaaa aagtgccgat cctgatcgcg ttccatcgcc atatctatga taccgattgg

361 cattatagct gcggcaccaa agaatataaa atcctgatgg atcagttcca tcatgtgagc

421 gcggcgttcc tggaactgga aaaaggctat caggaagcga tcgaagaaat cacccgccgc

481 atgggcgcgg gcatggcgaa attcatctgc caggaagtgg aaaccgtgga tgattatgat

541 gaatattgcc attatgtggc gggcctggtg ggcctgggcc tgagcaaact gttcctggcg

601 gcgggcagcg aagtgctgac cccggattgg gaagcgatca gcaacagcat gggcctgttc

661 ctgcagaaaa ccaacatcat ccgcgattat ctggaagata tcaacgaaat cccgaaaagc

721 cgcatgttct ggccgcgcga aatctggggc aaatatgcgg ataaactgga agatctgaaa

781 tatgaagaaa acaccaacaa aagcgtgcag tgcctgaacg aaatggtgac caacgcgctg

841 atgcatatcg aagattgcct gaaatatatg gtgagcctgc gcgatccgag catcttccgc

901 ttctgcgcga tcccgcagat catggcgatc ggcaccctgg cgctgtgcta taacaacgaa

961 caggtgttcc gcggcgtggt gaaactgcgc cgcggcctga ccgcgaaagt gatcgatcgc

1021 accaaaacca tggcggatgt gtatggcgcg ttctatgatt tcagctgcat gctgaaaacc

1081 aaagtggata aaaacgatcc gaacgcgagc aaaaccctga accgcctgga agcggtgcag

1141 aaactgtgcc gcgatgcggg cgtgctgcag aaccgcaaaa gctatgtgaa cgataaaggc

1201 cagccgaaca gcgtgttcat catcatggtg gtgatcctgc tggcgatcgt gttcgcgtat

1261 ctgcgcgcga actaactcga gatatgaagc ttgcggccgc actcgagcac caccaccacc

1321 accactgaga tccggctgct aacaaagccc gaaaggaagc tgagttggct gctgccaccg

1381 ctgagcaata actagcataa ccccttgggg cctctaaacg ggtcttgagg ggttttttg

//

**pRhon5Hi-2-SQS1-SQE1**

LOCUS SQS1-SQE1 3055 bp DNA linear SYN 24-OCT-2017

KEYWORDS pRhon5Hi-2-SQS1-SQE1

FEATURES Location/Qualifiers

regulatory 29..34

/regulatory_class=ribosome_binding_site

/gene="pRhotHi-2 RBS"

/product="RBS of vector pRhotHi-2"

CDS 43..1275

/gene="SQS1"

/product="A. thaliana squalene synthase SQS1"

/translation="MGSLGTMLRYPDDIYPLLKMKRAIEKAEKQIPPEPHWGFCYSML

HKVSRSFSLVIQQLNTELRNAVCVFYLVLRALDTVEDDTSIPTDEKVPILIAFHRHIY

DTDWHYSCGTKEYKILMDQFHHVSAAFLELEKGYQEAIEEITRRMGAGMAKFICQEVE

TVDDYDEYCHYVAGLVGLGLSKLFLAAGSEVLTPDWEAISNSMGLFLQKTNIIRDYLE

DINEIPKSRMFWPREIWGKYADKLEDLKYEENTNKSVQCLNEMVTNALMHIEDCLKYM

VSLRDPSIFRFCAIPQIMAIGTLALCYNNEQVFRGVVKLRRGLTAKVIDRTKTMADVY

GAFYDFSCMLKTKVDKNDPNASKTLNRLEAVQKLCRDAGVLQNRKSYVNDKGQPNSVF

IIMVVILLAIVFAYLRAN"

regulatory 1284..1289

/regulatory_class=ribosome_binding_site

/gene="pET RBS"

/product="RBS of pET vector series"

CDS 1298..2893

/gene="SQE1"

/product="A. thaliana squalene epoxidase SQE1"

/translation="MESQLWNWILPLLISSLLISFVAFYGFFVKPKRNGLRHDRKTVS

TVTSDVGSVNITGDTVADVIVVGAGVAGSALAYTLGKDKRRVHVIERDLSEPDRIVGE

LLQPGGYLKLLELGIEDCVEEIDAQRVYGYALFKNGKRIRLAYPLEKFHEDVSGRSFH

NGRFIQRMREKAASLPNVQLEQGTVLSLLEENGTIKGVRYKNKAGEEQTAFAALTIVC

DGCFSNLRRSLCNPQVEVPSCFVGLVLENCNLPYANHGHVVLADPSPILMYPISSTEV

RCLVDVPGQKVPSIANGEMKNYLKTVVAPQMPHEVYDSFIAAVDKGNIKSMPNRSMPA

SPYPTPGALLMGDAFNMRHPLTGGGMTVALADIVVLRNLLRPLRDLSDGASLCKYLES

FYTLRKPVAATINTLANALYQVFCSSENEARNEMREACFDYLGLGGMCTSGPVSLLSG

LNPRPLTLVCHFFAVAVYGVIRLLIPFPSPKRIWLGAKLISGASGIIFPIIKAEGVRQ

MFFPATVPAYYYKAPTVGETKCS"

regulatory 3008..3055

/regulatory_class=terminator

/gene="T7 terminator"

/product="bacteriophage T7 transcription terminator"

ORIGIN

1 tctagaaata attttgttta actttaagaa ggagatatac atatgggcag cctgggcacc

61 atgctgcgct atccggatga tatctatccg ctgctgaaaa tgaaacgcgc gatcgaaaaa

121 gcggaaaaac agatcccgcc ggaaccgcat tggggcttct gctatagcat gctgcataaa

181 gtgagccgca gcttcagcct ggtgatccag cagctgaaca ccgaactgcg caacgcggtg

241 tgcgtgttct atctggtgct gcgcgcgctg gataccgtgg aagatgatac cagcatcccg

301 accgatgaaa aagtgccgat cctgatcgcg ttccatcgcc atatctatga taccgattgg

361 cattatagct gcggcaccaa agaatataaa atcctgatgg atcagttcca tcatgtgagc

421 gcggcgttcc tggaactgga aaaaggctat caggaagcga tcgaagaaat cacccgccgc

481 atgggcgcgg gcatggcgaa attcatctgc caggaagtgg aaaccgtgga tgattatgat

541 gaatattgcc attatgtggc gggcctggtg ggcctgggcc tgagcaaact gttcctggcg

601 gcgggcagcg aagtgctgac cccggattgg gaagcgatca gcaacagcat gggcctgttc

661 ctgcagaaaa ccaacatcat ccgcgattat ctggaagata tcaacgaaat cccgaaaagc

721 cgcatgttct ggccgcgcga aatctggggc aaatatgcgg ataaactgga agatctgaaa

781 tatgaagaaa acaccaacaa aagcgtgcag tgcctgaacg aaatggtgac caacgcgctg

841 atgcatatcg aagattgcct gaaatatatg gtgagcctgc gcgatccgag catcttccgc

901 ttctgcgcga tcccgcagat catggcgatc ggcaccctgg cgctgtgcta taacaacgaa

961 caggtgttcc gcggcgtggt gaaactgcgc cgcggcctga ccgcgaaagt gatcgatcgc

1021 accaaaacca tggcggatgt gtatggcgcg ttctatgatt tcagctgcat gctgaaaacc

1081 aaagtggata aaaacgatcc gaacgcgagc aaaaccctga accgcctgga agcggtgcag

1141 aaactgtgcc gcgatgcggg cgtgctgcag aaccgcaaaa gctatgtgaa cgataaaggc

1201 cagccgaaca gcgtgttcat catcatggtg gtgatcctgc tggcgatcgt gttcgcgtat

1261 ctgcgcgcga actaactcga gataaggaga tatacacatg gaaagccagc tgtggaactg

1321 gatcctgccg ctgctgatca gcagcctgct gatcagcttc gtggcgttct atggcttctt

1381 cgtgaaaccg aaacgcaacg gcctgcgcca tgatcgcaaa accgtgagca ccgtgaccag

1441 cgatgtgggc agcgtgaaca tcaccggcga taccgtggcg gatgtgatcg tggtgggcgc

1501 gggcgtggcg ggcagcgcgc tggcgtatac cctgggcaaa gataaacgcc gcgtgcatgt

1561 gatcgaacgc gatctgagcg aaccggatcg catcgtgggc gaactgctgc agccgggcgg

1621 ctatctgaaa ctgctggaac tgggcatcga agattgcgtg gaagaaatcg atgcgcagcg

1681 cgtgtatggc tatgcgctgt tcaaaaacgg caaacgcatc cgcctggcgt atccgctgga

1741 aaaattccat gaagatgtga gcggccgcag cttccataac ggccgcttca tccagcgcat

1801 gcgcgaaaaa gcggcgagcc tgccgaacgt gcagctggaa cagggcaccg tgctgagcct

1861 gctggaagaa aacggcacca tcaaaggcgt gcgctataaa aacaaagcgg gcgaagaaca

1921 gaccgcgttc gcggcgctga ccatcgtgtg cgatggctgc ttcagcaacc tgcgccgcag

1981 cctgtgcaac ccgcaggtgg aagtgccgag ctgcttcgtg ggcctggtgc tggaaaactg

2041 caacctgccg tatgcgaacc atggccatgt ggtgctggcg gatccgagcc cgatcctgat

2101 gtatccgatc agcagcaccg aagtgcgctg cctggtggat gtgccgggcc agaaagtgcc

2161 gagcatcgcg aacggcgaaa tgaaaaacta tctgaaaacc gtggtggcgc cgcagatgcc

2221 gcatgaagtg tatgatagct tcatcgcggc ggtggataaa ggcaacatca aaagcatgcc

2281 gaaccgcagc atgccggcga gcccgtatcc gaccccgggc gcgctgctga tgggcgatgc

2341 gttcaacatg cgccatccgc tgaccggcgg cggcatgacc gtggcgctgg cggatatcgt

2401 ggtgctgcgc aacctgctgc gcccgctgcg cgatctgagc gatggcgcga gcctgtgcaa

2461 atatctggag agcttctata ccctgcgcaa accggtggcg gcgaccatca acaccctggc

2521 gaacgcgctg tatcaggtgt tctgcagcag cgaaaacgaa gcgcgcaacg aaatgcgcga

2581 agcgtgcttc gattatctgg gcctgggcgg catgtgcacc agcggcccgg tgagcctgct

2641 gagcggcctg aacccgcgcc cgctgaccct ggtgtgccat ttcttcgcgg tggcggtgta

2701 tggcgtgatc cgcctgctga tcccgttccc gagcccgaaa cgcatctggc tgggcgcgaa

2761 actgatcagc ggcgcgagcg gcatcatctt cccgatcatc aaagcggaag gcgtgcgcca

2821 gatgttcttc ccggcgaccg tgccggcgta ttattataaa gcgccgaccg tgggcgaaac

2881 caaatgcagc taaacgcgtt ataagcttgc ggccgcactc gagcaccacc accaccacca

2941 ctgagatccg gctgctaaca aagcccgaaa ggaagctgag ttggctgctg ccaccgctga

3001 gcaataacta gcataacccc ttggggcctc taaacgggtc ttgaggggtt ttttg

//

**pRhon5Hi-2-CAS1-SQS1-SQE1**

LOCUS CAS1-SQS1-SQE1 5357 bp DNA linear SYN 24-OCT-2017

KEYWORDS pRhon5Hi-2-CAS1-SQS1-SQE1

FEATURES Location/Qualifiers

regulatory 29..34

/regulatory_class=ribosome_binding_site

/gene="pRhotHi-2 RBS"

/product="RBS of vector pRhotHi-2"

CDS 43..2322

/gene="CAS1"

/product="A. thaliana cycloartenol synthase CAS1"

/translation="MWKLKIAEGGSPWLRTTNNHVGRQFWEFDPNLGTPEDLAAVEEA

RKSFSDNRFVQKHSADLLMRLQFSRENLISPVLPQVKIEDTDDVTEEMVETTLKRGLD

FYSTIQAHDGHWPGDYGGPMFLLPGLIITLSITGALNTVLSEQHKQEMRRYLYNHQNE

DGGWGLHIEGPSTMFGSVLNYVTLRLLGEGPNDGDGDMEKGRDWILNHGGATNITSWG

KMWLSVLGAFEWSGNNPLPPEIWLLPYFLPIHPGRMWCHCRMVYLPMSYLYGKRFVGP

ITSTVLSLRKELFTVPYHEVNWNEARNLCAKEDLYYPHPLVQDILWASLHKIVEPVLM

RWPGANLREKAIRTAIEHIHYEDENTRYICIGPVNKVLNMLCCWVEDPNSEAFKLHLP

RIHDFLWLAEDGMKMQGYNGSQLWDTGFAIQAILATNLVEEYGPVLEKAHSFVKNSQV

LEDCPGDLNYWYRHISKGAWPFSTADHGWPISDCTAEGLKAALLLSKVPKAIVGEPID

AKRLYEAVNVIISLQNADGGLATYELTRSYPWLELINPAETFGDIVIDYPYVECTSAA

IQALISFRKLYPGHRKKEVDECIEKAVKFIESIQAADGSWYGSWAVCFTYGTWFGVKG

LVAVGKTLKNSPHVAKACEFLLSKQQPSGGWGESYLSCQDKVYSNLDGNRSHVVNTAW

AMLALIGAGQAEVDRKPLHRAARYLINAQMENGDFPQQEIMGVFNRNCMITYAAYRNI

FPIWALGEYRCQVLLQQGE"

regulatory 2323..2341

/regulatory_class=ribosome_binding_site

/gene="nifK RBS"

/product="5'UTR of R. capsulatus nifK incl RBS"

CDS 2345..3577

/gene="SQS1"

/product="A. thaliana squalene synthase SQS1"

/translation="MGSLGTMLRYPDDIYPLLKMKRAIEKAEKQIPPEPHWGFCYSML

HKVSRSFSLVIQQLNTELRNAVCVFYLVLRALDTVEDDTSIPTDEKVPILIAFHRHIY

DTDWHYSCGTKEYKILMDQFHHVSAAFLELEKGYQEAIEEITRRMGAGMAKFICQEVE

TVDDYDEYCHYVAGLVGLGLSKLFLAAGSEVLTPDWEAISNSMGLFLQKTNIIRDYLE

DINEIPKSRMFWPREIWGKYADKLEDLKYEENTNKSVQCLNEMVTNALMHIEDCLKYM

VSLRDPSIFRFCAIPQIMAIGTLALCYNNEQVFRGVVKLRRGLTAKVIDRTKTMADVY

GAFYDFSCMLKTKVDKNDPNASKTLNRLEAVQKLCRDAGVLQNRKSYVNDKGQPNSVF

IIMVVILLAIVFAYLRAN"

regulatory 3586..3591

/regulatory_class=ribosome_binding_site

/gene="pET RBS"

/product="RBS of pET vector series"

CDS 3600..5195

/gene="SQE1"

/product="A. thaliana squalene epoxidase SQE1"

/translation="MESQLWNWILPLLISSLLISFVAFYGFFVKPKRNGLRHDRKTVS

TVTSDVGSVNITGDTVADVIVVGAGVAGSALAYTLGKDKRRVHVIERDLSEPDRIVGE

LLQPGGYLKLLELGIEDCVEEIDAQRVYGYALFKNGKRIRLAYPLEKFHEDVSGRSFH

NGRFIQRMREKAASLPNVQLEQGTVLSLLEENGTIKGVRYKNKAGEEQTAFAALTIVC

DGCFSNLRRSLCNPQVEVPSCFVGLVLENCNLPYANHGHVVLADPSPILMYPISSTEV

RCLVDVPGQKVPSIANGEMKNYLKTVVAPQMPHEVYDSFIAAVDKGNIKSMPNRSMPA

SPYPTPGALLMGDAFNMRHPLTGGGMTVALADIVVLRNLLRPLRDLSDGASLCKYLES

FYTLRKPVAATINTLANALYQVFCSSENEARNEMREACFDYLGLGGMCTSGPVSLLSG

LNPRPLTLVCHFFAVAVYGVIRLLIPFPSPKRIWLGAKLISGASGIIFPIIKAEGVRQ

MFFPATVPAYYYKAPTVGETKCS"

regulatory 5310..5357

/regulatory_class=terminator

/gene="T7 terminator"

/product="bacteriophage T7 transcription terminator"

ORIGIN

1 tctagaaata attttgttta actttaagaa ggagatatac atatgtggaa actgaaaatc

61 gcggaaggcg gcagcccgtg gctgcgcacc accaacaacc atgtgggccg ccagttctgg

121 gagttcgatc cgaacctggg caccccggaa gatctggcgg cggtggaaga agcgcgcaag

181 agcttcagcg ataaccgctt cgtgcagaaa catagcgcgg atctgctgat gcgcctgcag

241 ttcagccgcg aaaacctgat cagcccggtg ctgccgcagg tgaaaatcga agataccgat

301 gatgtgaccg aagaaatggt ggaaaccacc ctgaaacgcg gcctggattt ctatagcacc

361 atccaggcgc atgatggcca ttggccgggc gattatggcg gcccgatgtt cctgctgccg

421 ggcctgatca tcaccctgag catcaccggc gcgctgaaca ccgtgctgag cgaacagcat

481 aaacaggaaa tgcgccgcta tctgtataac catcagaacg aagatggcgg ctggggcctg

541 catatcgaag gcccgagcac catgttcggc agcgtgctga actatgtgac cctgcgcctg

601 ctgggcgaag gcccgaacga tggcgatggc gatatggaaa aaggccgcga ttggatcctg

661 aaccatggcg gcgcgaccaa catcaccagc tggggcaaaa tgtggctgag cgtgctgggc

721 gcgttcgaat ggagcggcaa caacccgctg ccgccggaaa tctggctgct gccgtatttc

781 ctgccgatcc atccgggccg catgtggtgc cattgccgca tggtgtatct gccgatgagc

841 tatctgtatg gcaaacgctt cgtgggcccg atcaccagca ccgtgctgag cctgcgcaaa

901 gaactgttca ccgtgccgta tcatgaagtg aactggaacg aagcgcgcaa cctgtgcgcg

961 aaagaagatc tgtattatcc gcatccgctg gtgcaggata tcctgtgggc gagcctgcat

1021 aaaatcgtgg aaccggtgct gatgcgctgg ccgggcgcga acctgcgcga aaaagcgatc

1081 cgcaccgcga tcgaacatat ccattatgaa gatgaaaaca cccgctatat ctgcatcggc

1141 ccggtgaaca aagtgctgaa catgctgtgc tgctgggtgg aagatccgaa cagcgaagcg

1201 ttcaaactgc atctgccgcg catccatgat ttcctgtggc tggcggaaga tggcatgaaa

1261 atgcagggct ataacggcag ccagctgtgg gataccggct tcgcgatcca ggcgatcctg

1321 gcgaccaacc tggtggaaga atatggcccg gtgctggaaa aagcgcatag cttcgtgaaa

1381 aacagccagg tgctggaaga ttgcccgggc gatctgaact attggtatcg ccatatcagc

1441 aaaggcgcgt ggccgttcag caccgcggat catggctggc cgatcagcga ttgcaccgcg

1501 gaaggcctga aagcggcgct gctgctgagc aaagtgccga aagcgatcgt gggcgaaccg

1561 atcgatgcga aacgcctgta tgaagcggtg aacgtgatca tcagcctgca gaacgcggat

1621 ggcggcctgg cgacctatga actgacccgc agctatccgt ggctggaact gatcaacccg

1681 gcggaaacct tcggcgatat cgtgatcgat tatccgtatg tggaatgcac cagcgcggcg

1741 atccaggcgc tgatcagctt ccgcaaactg tatccgggcc atcgcaaaaa agaagtggat

1801 gaatgcatcg aaaaagcggt gaaattcatc gaaagcatcc aggcggcgga tggcagctgg

1861 tatggcagct gggcggtgtg cttcacctat ggcacctggt tcggcgtgaa aggcctggtg

1921 gcggtgggca aaaccctgaa aaacagcccg catgtggcga aagcgtgcga gttcctgctg

1981 agcaaacagc agccgagcgg cggctggggc gaaagctatc tgagctgcca ggataaagtg

2041 tatagcaacc tggatggcaa ccgcagccat gtggtgaaca ccgcgtgggc gatgctggcg

2101 ctgatcggcg cgggccaggc ggaagtggat cgcaaaccgc tgcatcgcgc ggcgcgctat

2161 ctgatcaacg cgcagatgga aaacggcgat ttcccgcagc aggaaatcat gggcgtgttc

2221 aaccgcaact gcatgatcac ctatgcggcg tatcgcaaca tcttcccgat ctgggcgctg

2281 ggcgaatatc gctgccaggt gctgctgcag cagggcgaat aagcctttga caaggaattg

2341 acatatgggc agcctgggca ccatgctgcg ctatccggat gatatctatc cgctgctgaa

2401 aatgaaacgc gcgatcgaaa aagcggaaaa acagatcccg ccggaaccgc attggggctt

2461 ctgctatagc atgctgcata aagtgagccg cagcttcagc ctggtgatcc agcagctgaa

2521 caccgaactg cgcaacgcgg tgtgcgtgtt ctatctggtg ctgcgcgcgc tggataccgt

2581 ggaagatgat accagcatcc cgaccgatga aaaagtgccg atcctgatcg cgttccatcg

2641 ccatatctat gataccgatt ggcattatag ctgcggcacc aaagaatata aaatcctgat

2701 ggatcagttc catcatgtga gcgcggcgtt cctggaactg gaaaaaggct atcaggaagc

2761 gatcgaagaa atcacccgcc gcatgggcgc gggcatggcg aaattcatct gccaggaagt

2821 ggaaaccgtg gatgattatg atgaatattg ccattatgtg gcgggcctgg tgggcctggg

2881 cctgagcaaa ctgttcctgg cggcgggcag cgaagtgctg accccggatt gggaagcgat

2941 cagcaacagc atgggcctgt tcctgcagaa aaccaacatc atccgcgatt atctggaaga

3001 tatcaacgaa atcccgaaaa gccgcatgtt ctggccgcgc gaaatctggg gcaaatatgc

3061 ggataaactg gaagatctga aatatgaaga aaacaccaac aaaagcgtgc agtgcctgaa

3121 cgaaatggtg accaacgcgc tgatgcatat cgaagattgc ctgaaatata tggtgagcct

3181 gcgcgatccg agcatcttcc gcttctgcgc gatcccgcag atcatggcga tcggcaccct

3241 ggcgctgtgc tataacaacg aacaggtgtt ccgcggcgtg gtgaaactgc gccgcggcct

3301 gaccgcgaaa gtgatcgatc gcaccaaaac catggcggat gtgtatggcg cgttctatga

3361 tttcagctgc atgctgaaaa ccaaagtgga taaaaacgat ccgaacgcga gcaaaaccct

3421 gaaccgcctg gaagcggtgc agaaactgtg ccgcgatgcg ggcgtgctgc agaaccgcaa

3481 aagctatgtg aacgataaag gccagccgaa cagcgtgttc atcatcatgg tggtgatcct

3541 gctggcgatc gtgttcgcgt atctgcgcgc gaactaactc gagataagga gatatacaca

3601 tggaaagcca gctgtggaac tggatcctgc cgctgctgat cagcagcctg ctgatcagct

3661 tcgtggcgtt ctatggcttc ttcgtgaaac cgaaacgcaa cggcctgcgc catgatcgca

3721 aaaccgtgag caccgtgacc agcgatgtgg gcagcgtgaa catcaccggc gataccgtgg

3781 cggatgtgat cgtggtgggc gcgggcgtgg cgggcagcgc gctggcgtat accctgggca

3841 aagataaacg ccgcgtgcat gtgatcgaac gcgatctgag cgaaccggat cgcatcgtgg

3901 gcgaactgct gcagccgggc ggctatctga aactgctgga actgggcatc gaagattgcg

3961 tggaagaaat cgatgcgcag cgcgtgtatg gctatgcgct gttcaaaaac ggcaaacgca

4021 tccgcctggc gtatccgctg gaaaaattcc atgaagatgt gagcggccgc agcttccata

4081 acggccgctt catccagcgc atgcgcgaaa aagcggcgag cctgccgaac gtgcagctgg

4141 aacagggcac cgtgctgagc ctgctggaag aaaacggcac catcaaaggc gtgcgctata

4201 aaaacaaagc gggcgaagaa cagaccgcgt tcgcggcgct gaccatcgtg tgcgatggct

4261 gcttcagcaa cctgcgccgc agcctgtgca acccgcaggt ggaagtgccg agctgcttcg

4321 tgggcctggt gctggaaaac tgcaacctgc cgtatgcgaa ccatggccat gtggtgctgg

4381 cggatccgag cccgatcctg atgtatccga tcagcagcac cgaagtgcgc tgcctggtgg

4441 atgtgccggg ccagaaagtg ccgagcatcg cgaacggcga aatgaaaaac tatctgaaaa

4501 ccgtggtggc gccgcagatg ccgcatgaag tgtatgatag cttcatcgcg gcggtggata

4561 aaggcaacat caaaagcatg ccgaaccgca gcatgccggc gagcccgtat ccgaccccgg

4621 gcgcgctgct gatgggcgat gcgttcaaca tgcgccatcc gctgaccggc ggcggcatga

4681 ccgtggcgct ggcggatatc gtggtgctgc gcaacctgct gcgcccgctg cgcgatctga

4741 gcgatggcgc gagcctgtgc aaatatctgg agagcttcta taccctgcgc aaaccggtgg

4801 cggcgaccat caacaccctg gcgaacgcgc tgtatcaggt gttctgcagc agcgaaaacg

4861 aagcgcgcaa cgaaatgcgc gaagcgtgct tcgattatct gggcctgggc ggcatgtgca

4921 ccagcggccc ggtgagcctg ctgagcggcc tgaacccgcg cccgctgacc ctggtgtgcc

4981 atttcttcgc ggtggcggtg tatggcgtga tccgcctgct gatcccgttc ccgagcccga

5041 aacgcatctg gctgggcgcg aaactgatca gcggcgcgag cggcatcatc ttcccgatca

5101 tcaaagcgga aggcgtgcgc cagatgttct tcccggcgac cgtgccggcg tattattata

5161 aagcgccgac cgtgggcgaa accaaatgca gctaaacgcg ttataagctt gcggccgcac

5221 tcgagcacca ccaccaccac cactgagatc cggctgctaa caaagcccga aaggaagctg

5281 agttggctgc tgccaccgct gagcaataac tagcataacc ccttggggcc tctaaacggg

5341 tcttgagggg ttttttg

//

**pRhon5Hi-2-LUP1-SQS1-SQE1**

LOCUS LUP1-SQS1-SQE1 5351 bp DNA linear SYN 24-OCT-2017

KEYWORDS pRhon5Hi-2-LUP1-SQS1-SQE1

FEATURES Location/Qualifiers

regulatory 29..34

/regulatory_class=ribosome_binding_site

/gene="pRhotHi-2 RBS"

/product="RBS of vector pRhotHi-2"

CDS 43..2316

/gene="LUP1"

/product="A. thaliana lupeol synthase LUP1"

/translation="MWKLKIGKGNGEDPHLFSSNNFVGRQTWKFDHKAGSPEERAAVE

EARRGFLDNRFRVKGCSDLLWRMQFLREKKFEQGIPQLKATNIEEITYETTTNALRRG

VRYFTALQASDGHWPGEITGPLFFLPPLIFCLYITGHLEEVFDAEHRKEMLRHIYCHQ

NEDGGWGLHIESKSVMFCTVLNYICLRMLGENPEQDACKRARQWILDRGGVIFIPSWG

KFWLSILGVYDWSGTNPTPPELLMLPSFLPIHPGKILCYSRMVSIPMSYLYGKRFVGP

ITPLILLLREELYLEPYEEINWKKSRRLYAKEDMYYAHPLVQDLLSDTLQNFVEPLLT

RWPLNKLVREKALQLTMKHIHYEDENSHYITIGCVEKVLCMLACWVENPNGDYFKKHL

ARIPDYMWVAEDGMKMQSFGCQLWDTGFAIQALLASNLPDETDDALKRGHNYIKASQV

RENPSGDFRSMYRHISKGAWTFSDRDHGWQVSDCTAEALKCCLLLSMMSADIVGQKID

DEQLYDSVNLLLSLQSGNGGVNAWEPSRAYKWLELLNPTEFMANTMVEREFVECTSSV

IQALDLFRKLYPDHRKKEINRSIEKAVQFIQDNQTPDGSWYGNWGVCFIYATWFALGG

LAAAGETYNDCLAMRNGVHFLLTTQRDDGGWGESYLSCSEQRYIPSEGERSNLVQTSW

AMMALIHTGQAERDLIPLHRAAKLIINSQLENGDFPQQEIVGAFMNTCMLHYATYRNT

FPLWALAEYRKVVFIVN"

regulatory 2317..2335

/regulatory_class=ribosome_binding_site

/gene="nifK RBS"

/product="5'UTR of R. capsulatus nifK incl RBS"

CDS 2339..3571

/gene="SQS1"

/product="A. thaliana squalene synthase SQS1"

/translation="MGSLGTMLRYPDDIYPLLKMKRAIEKAEKQIPPEPHWGFCYSML

HKVSRSFSLVIQQLNTELRNAVCVFYLVLRALDTVEDDTSIPTDEKVPILIAFHRHIY

DTDWHYSCGTKEYKILMDQFHHVSAAFLELEKGYQEAIEEITRRMGAGMAKFICQEVE

TVDDYDEYCHYVAGLVGLGLSKLFLAAGSEVLTPDWEAISNSMGLFLQKTNIIRDYLE

DINEIPKSRMFWPREIWGKYADKLEDLKYEENTNKSVQCLNEMVTNALMHIEDCLKYM

VSLRDPSIFRFCAIPQIMAIGTLALCYNNEQVFRGVVKLRRGLTAKVIDRTKTMADVY

GAFYDFSCMLKTKVDKNDPNASKTLNRLEAVQKLCRDAGVLQNRKSYVNDKGQPNSVF

IIMVVILLAIVFAYLRAN"

regulatory 3580..3585

/regulatory_class=ribosome_binding_site

/gene="pET RBS"

/product="RBS of pET vector series"

CDS 3594..5189

/gene="SQE1"

/product="A. thaliana squalene epoxidase SQE1"

/translation="MESQLWNWILPLLISSLLISFVAFYGFFVKPKRNGLRHDRKTVS

TVTSDVGSVNITGDTVADVIVVGAGVAGSALAYTLGKDKRRVHVIERDLSEPDRIVGE

LLQPGGYLKLLELGIEDCVEEIDAQRVYGYALFKNGKRIRLAYPLEKFHEDVSGRSFH

NGRFIQRMREKAASLPNVQLEQGTVLSLLEENGTIKGVRYKNKAGEEQTAFAALTIVC

DGCFSNLRRSLCNPQVEVPSCFVGLVLENCNLPYANHGHVVLADPSPILMYPISSTEV

RCLVDVPGQKVPSIANGEMKNYLKTVVAPQMPHEVYDSFIAAVDKGNIKSMPNRSMPA

SPYPTPGALLMGDAFNMRHPLTGGGMTVALADIVVLRNLLRPLRDLSDGASLCKYLES

FYTLRKPVAATINTLANALYQVFCSSENEARNEMREACFDYLGLGGMCTSGPVSLLSG

LNPRPLTLVCHFFAVAVYGVIRLLIPFPSPKRIWLGAKLISGASGIIFPIIKAEGVRQ

MFFPATVPAYYYKAPTVGETKCS"

regulatory 5304..5351

/regulatory_class=terminator

/gene="T7 terminator"

/product="bacteriophage T7 transcription terminator"

ORIGIN

1 tctagaaata attttgttta actttaagaa ggagatatac atatgtggaa actgaagatc

61 gggaagggca acggcgagga tccccatctg ttttcgtcga acaacttcgt cggccgccaa

121 acgtggaagt tcgaccataa ggccggcagc ccggaagagc gcgccgccgt ggaggaggcc

181 cgccggggct tcctggacaa ccggttccgc gtcaagggct gctccgacct gctgtggcgc

241 atgcagttcc tgcgcgaaaa aaaattcgaa cagggcatcc cccagctgaa agccacgaac

301 atcgaggaaa tcacgtatga gaccacgacg aacgcgctgc ggcgcggggt gcgctatttc

361 acggcgctgc aggcgtccga cggccactgg cccggcgaaa tcaccggccc gctgtttttc

421 ctgccgccgc ttatcttctg cctgtatatc acgggccacc tggaagaagt gttcgacgcg

481 gaacaccgca aggagatgct gcggcatatc tattgccatc agaatgaaga cggcggctgg

541 ggcctgcaca tcgagtcgaa aagcgtgatg ttttgcacgg tcctgaacta catctgcctg

601 cggatgctgg gggaaaaccc cgagcaggac gcctgcaaac gcgcccgcca atggatcctg

661 gatcgcgggg gggtgatctt tatcccgtcg tggggcaagt tctggctgag catcctgggc

721 gtctacgatt ggtcggggac caacccgacc ccgcccgagc tgctgatgct gcccagcttt

781 ctgccgatcc acccgggcaa gatcctgtgc tacagccgca tggtgagcat cccgatgtcc

841 tatctgtatg ggaagcggtt cgtcggcccg atcacgccgc tgatccttct gctgcgcgag

901 gaactgtacc tggagcccta tgaagagatc aattggaaga agagccgccg cctgtacgcg

961 aaggaggaca tgtactatgc gcacccgctg gtgcaagatc tgctgtcgga caccctgcag

1021 aactttgtcg agccgctgct gacgcgctgg cccctgaata agctggtgcg ggagaaggcg

1081 ctgcagctta ccatgaaaca tatccactac gaggacgaaa attcgcatta catcacgatc

1141 ggctgcgtcg aaaaggtcct gtgcatgctg gcctgctggg tggaaaaccc gaacggggac

1201 tattttaaga agcacctggc gcggatcccg gattatatgt gggtggcgga agacgggatg

1261 aaaatgcaga gcttcggctg ccagctgtgg gatacgggct tcgcgatcca ggccctgctg

1321 gcgtcgaatc tgccggacga gaccgatgac gcccttaagc ggggccacaa ctacatcaag

1381 gcctcccagg tccgcgaaaa tccgagcggc gacttccgca gcatgtaccg gcatatctcg

1441 aaaggcgcct ggaccttttc ggaccgggat catggctggc aggtgtccga ctgcaccgcg

1501 gaagccctga agtgctgcct gctgcttagc atgatgtcgg cggacatcgt cgggcagaag

1561 atcgatgacg agcaactgta tgactcggtg aatctgctgc tttcgcttca gtcggggaac

1621 ggcggcgtca acgcctggga gccgtcgcgc gcgtataaat ggctggagct gctgaatccc

1681 accgagttca tggccaatac gatggtcgaa cgcgaattcg tggaatgcac gtcgtccgtg

1741 atccaggcgc tggatctgtt tcggaaactg tatcccgacc atcggaagaa ggagatcaat

1801 cgctcgatcg agaaagcggt ccagttcatc caggacaacc agacgccgga tggctcctgg

1861 tacggcaact ggggggtgtg cttcatctac gccacctggt tcgccctggg gggcctggcg

1921 gcggccggcg agacctataa cgattgcctg gcgatgcgga acggggtgca tttcctgctg

1981 accacccagc gcgacgatgg gggctggggc gagtcctacc tgagctgctc cgagcagcgc

2041 tatatcccgt cggaaggcga acggtcgaac ctggtccaga cgtcgtgggc gatgatggcc

2101 ctgatccata ccgggcaggc ggagcgcgac ctgatcccgc tgcatcgggc ggcgaaactg

2161 atcatcaaca gccagctgga aaacggcgac tttccgcagc aggaaatcgt gggcgccttc

2221 atgaacacct gcatgctgca ttacgcgacg taccgcaaca ccttccccct gtgggcgctg

2281 gcggagtatc gcaaggtcgt cttcatcgtc aattaagcct ttgacaagga attgacatat

2341 gggcagcctg ggcaccatgc tgcgctatcc ggatgatatc tatccgctgc tgaaaatgaa

2401 acgcgcgatc gaaaaagcgg aaaaacagat cccgccggaa ccgcattggg gcttctgcta

2461 tagcatgctg cataaagtga gccgcagctt cagcctggtg atccagcagc tgaacaccga

2521 actgcgcaac gcggtgtgcg tgttctatct ggtgctgcgc gcgctggata ccgtggaaga

2581 tgataccagc atcccgaccg atgaaaaagt gccgatcctg atcgcgttcc atcgccatat

2641 ctatgatacc gattggcatt atagctgcgg caccaaagaa tataaaatcc tgatggatca

2701 gttccatcat gtgagcgcgg cgttcctgga actggaaaaa ggctatcagg aagcgatcga

2761 agaaatcacc cgccgcatgg gcgcgggcat ggcgaaattc atctgccagg aagtggaaac

2821 cgtggatgat tatgatgaat attgccatta tgtggcgggc ctggtgggcc tgggcctgag

2881 caaactgttc ctggcggcgg gcagcgaagt gctgaccccg gattgggaag cgatcagcaa

2941 cagcatgggc ctgttcctgc agaaaaccaa catcatccgc gattatctgg aagatatcaa

3001 cgaaatcccg aaaagccgca tgttctggcc gcgcgaaatc tggggcaaat atgcggataa

3061 actggaagat ctgaaatatg aagaaaacac caacaaaagc gtgcagtgcc tgaacgaaat

3121 ggtgaccaac gcgctgatgc atatcgaaga ttgcctgaaa tatatggtga gcctgcgcga

3181 tccgagcatc ttccgcttct gcgcgatccc gcagatcatg gcgatcggca ccctggcgct

3241 gtgctataac aacgaacagg tgttccgcgg cgtggtgaaa ctgcgccgcg gcctgaccgc

3301 gaaagtgatc gatcgcacca aaaccatggc ggatgtgtat ggcgcgttct atgatttcag

3361 ctgcatgctg aaaaccaaag tggataaaaa cgatccgaac gcgagcaaaa ccctgaaccg

3421 cctggaagcg gtgcagaaac tgtgccgcga tgcgggcgtg ctgcagaacc gcaaaagcta

3481 tgtgaacgat aaaggccagc cgaacagcgt gttcatcatc atggtggtga tcctgctggc

3541 gatcgtgttc gcgtatctgc gcgcgaacta actcgagata aggagatata cacatggaaa

3601 gccagctgtg gaactggatc ctgccgctgc tgatcagcag cctgctgatc agcttcgtgg

3661 cgttctatgg cttcttcgtg aaaccgaaac gcaacggcct gcgccatgat cgcaaaaccg

3721 tgagcaccgt gaccagcgat gtgggcagcg tgaacatcac cggcgatacc gtggcggatg

3781 tgatcgtggt gggcgcgggc gtggcgggca gcgcgctggc gtataccctg ggcaaagata

3841 aacgccgcgt gcatgtgatc gaacgcgatc tgagcgaacc ggatcgcatc gtgggcgaac

3901 tgctgcagcc gggcggctat ctgaaactgc tggaactggg catcgaagat tgcgtggaag

3961 aaatcgatgc gcagcgcgtg tatggctatg cgctgttcaa aaacggcaaa cgcatccgcc

4021 tggcgtatcc gctggaaaaa ttccatgaag atgtgagcgg ccgcagcttc cataacggcc

4081 gcttcatcca gcgcatgcgc gaaaaagcgg cgagcctgcc gaacgtgcag ctggaacagg

4141 gcaccgtgct gagcctgctg gaagaaaacg gcaccatcaa aggcgtgcgc tataaaaaca

4201 aagcgggcga agaacagacc gcgttcgcgg cgctgaccat cgtgtgcgat ggctgcttca

4261 gcaacctgcg ccgcagcctg tgcaacccgc aggtggaagt gccgagctgc ttcgtgggcc

4321 tggtgctgga aaactgcaac ctgccgtatg cgaaccatgg ccatgtggtg ctggcggatc

4381 cgagcccgat cctgatgtat ccgatcagca gcaccgaagt gcgctgcctg gtggatgtgc

4441 cgggccagaa agtgccgagc atcgcgaacg gcgaaatgaa aaactatctg aaaaccgtgg

4501 tggcgccgca gatgccgcat gaagtgtatg atagcttcat cgcggcggtg gataaaggca

4561 acatcaaaag catgccgaac cgcagcatgc cggcgagccc gtatccgacc ccgggcgcgc

4621 tgctgatggg cgatgcgttc aacatgcgcc atccgctgac cggcggcggc atgaccgtgg

4681 cgctggcgga tatcgtggtg ctgcgcaacc tgctgcgccc gctgcgcgat ctgagcgatg

4741 gcgcgagcct gtgcaaatat ctggagagct tctataccct gcgcaaaccg gtggcggcga

4801 ccatcaacac cctggcgaac gcgctgtatc aggtgttctg cagcagcgaa aacgaagcgc

4861 gcaacgaaat gcgcgaagcg tgcttcgatt atctgggcct gggcggcatg tgcaccagcg

4921 gcccggtgag cctgctgagc ggcctgaacc cgcgcccgct gaccctggtg tgccatttct

4981 tcgcggtggc ggtgtatggc gtgatccgcc tgctgatccc gttcccgagc ccgaaacgca

5041 tctggctggg cgcgaaactg atcagcggcg cgagcggcat catcttcccg atcatcaaag

5101 cggaaggcgt gcgccagatg ttcttcccgg cgaccgtgcc ggcgtattat tataaagcgc

5161 cgaccgtggg cgaaaccaaa tgcagctaaa cgcgttataa gcttgcggcc gcactcgagc

5221 accaccacca ccaccactga gatccggctg ctaacaaagc ccgaaaggaa gctgagttgg

5281 ctgctgccac cgctgagcaa taactagcat aaccccttgg ggcctctaaa cgggtcttga

5341 ggggtttttt g

//

**pRhon5Hi-2-THAS1-SQS1-SQE1**

LOCUS THAS1-SQS1-SQE1 5354 bp DNA linear SYN 24-OCT-2017

KEYWORDS pRhon5Hi-2-THAS1-SQS1-SQE1

FEATURES Location/Qualifiers

regulatory 29..34

/regulatory_class=ribosome_binding_site

/gene="pRhotHi-2 RBS"

/product="RBS of vector pRhotHi-2"

CDS 43..2319

/gene="THAS1"

/product="A. thaliana thalianol synthase THAS1"

/translation="MWRLRTGPKAGEDTHLFTTNNYAGRQIWEFDANAGSPQEIAEVE

DARHKFSDNTSRFKTTADLLWRMQFLREKKFEQKIPRVIIEDARKIKYEDAKTALKRG

LLYFTALQADDGHWPAENSGPNFYTPPFLICLYITGHLEKIFTPEHVKELLRHIYNMQ

NEDGGWGLHVESHSVMFCTVINYVCLRIVGEEVGHDDQRNGCAKAHKWIMDHGGATYT

PLIGKALLSVLGVYDWSGCNPIPPEFWLLPSSFPVNGGTLWIYLRDTFMGLSYLYGKK

FVAPPTPLILQLREELYPEPYAKINWTQTRNRCGKEDLYYPRSFLQDLFWKSVHMFSE

SILDRWPLNKLIRQRALQSTMALIHYHDESTRYITGGCLPKAFHMLACWIEDPKSDYF

KKHLARVREYIWIGEDGLKIQSFGSQLWDTALSLHALLDGIDDHDVDDEIKTTLVKGY

DYLKKSQITENPRGDHFKMFRHKTKGGWTFSDQDQGWPVSDCTAESLECCLFFESMPS

ELIGKKMDVEKLYDAVDYLLYLQSDNGGIAAWQPVEGKAWLELLNIMIFRYVECTGSA

IAALTQFNKQFPGYKNVEVKRFITKAAKYIEDMQTVDGSWYGNWGVCFIYGTFFAVRG

LVAAGKTYSNCEAIRKAVRFLLDTQNPEGGWGESFLSCPSKKYTPLKGNSTNVVQTAQ

ALMVLIMGDQMERDPLPVHRAAQVLINSQLDNGDFPQQEIMGTFMRTVMLHFPTYRNT

FSLWALTHYTHALRRLLP"

regulatory 2320..2338

/regulatory_class=ribosome_binding_site

/gene="nifK RBS"

/product="5'UTR of R. capsulatus nifK incl RBS"

CDS 2342..3574

/gene="SQS1"

/product="A. thaliana squalene synthase SQS1"

/translation="MGSLGTMLRYPDDIYPLLKMKRAIEKAEKQIPPEPHWGFCYSML

HKVSRSFSLVIQQLNTELRNAVCVFYLVLRALDTVEDDTSIPTDEKVPILIAFHRHIY

DTDWHYSCGTKEYKILMDQFHHVSAAFLELEKGYQEAIEEITRRMGAGMAKFICQEVE

TVDDYDEYCHYVAGLVGLGLSKLFLAAGSEVLTPDWEAISNSMGLFLQKTNIIRDYLE

DINEIPKSRMFWPREIWGKYADKLEDLKYEENTNKSVQCLNEMVTNALMHIEDCLKYM

VSLRDPSIFRFCAIPQIMAIGTLALCYNNEQVFRGVVKLRRGLTAKVIDRTKTMADVY

GAFYDFSCMLKTKVDKNDPNASKTLNRLEAVQKLCRDAGVLQNRKSYVNDKGQPNSVF

IIMVVILLAIVFAYLRAN"

regulatory 3583..3588

/regulatory_class=ribosome_binding_site

/gene="pET RBS"

/product="RBS of pET vector series"

CDS 3597..5192

/gene="SQE1"

/product="A. thaliana squalene epoxidase SQE1"

/translation="MESQLWNWILPLLISSLLISFVAFYGFFVKPKRNGLRHDRKTVS

TVTSDVGSVNITGDTVADVIVVGAGVAGSALAYTLGKDKRRVHVIERDLSEPDRIVGE

LLQPGGYLKLLELGIEDCVEEIDAQRVYGYALFKNGKRIRLAYPLEKFHEDVSGRSFH

NGRFIQRMREKAASLPNVQLEQGTVLSLLEENGTIKGVRYKNKAGEEQTAFAALTIVC

DGCFSNLRRSLCNPQVEVPSCFVGLVLENCNLPYANHGHVVLADPSPILMYPISSTEV

RCLVDVPGQKVPSIANGEMKNYLKTVVAPQMPHEVYDSFIAAVDKGNIKSMPNRSMPA

SPYPTPGALLMGDAFNMRHPLTGGGMTVALADIVVLRNLLRPLRDLSDGASLCKYLES

FYTLRKPVAATINTLANALYQVFCSSENEARNEMREACFDYLGLGGMCTSGPVSLLSG

LNPRPLTLVCHFFAVAVYGVIRLLIPFPSPKRIWLGAKLISGASGIIFPIIKAEGVRQ

MFFPATVPAYYYKAPTVGETKCS"

regulatory 5307..5354

/regulatory_class=terminator

/gene="T7 terminator"

/product="bacteriophage T7 transcription terminator"

ORIGIN

1 tctagaaata attttgttta actttaagaa ggagatatac atatgtggcg cctgcgcacc

61 ggcccgaaag cgggcgaaga tacccatctg ttcaccacca acaactatgc gggccgccag

121 atctgggaat tcgatgcgaa cgcgggcagc ccgcaggaaa tcgcggaagt ggaagatgcg

181 cgccataaat tcagcgataa caccagccgc ttcaaaacca ccgcggatct gctgtggcgc

241 atgcagttcc tgcgcgaaaa aaaattcgaa cagaaaatcc cgcgcgtgat catcgaagat

301 gcgcgcaaaa tcaaatatga agatgcgaaa accgcgctga aacgcggcct gctgtatttc

361 accgcgctgc aggcggatga tggccattgg ccggcggaaa acagcggccc gaacttctat

421 accccgccgt tcctgatctg cctgtatatc accggccatc tggaaaaaat cttcaccccg

481 gaacatgtga aagaactgct gcgccatatc tataacatgc agaacgaaga tggcggctgg

541 ggcctgcatg tggaaagcca tagcgtgatg ttctgcaccg tgatcaacta tgtgtgcctg

601 cgcatcgtgg gcgaagaagt gggccatgat gatcagcgca acggctgcgc gaaagcgcat

661 aaatggatca tggatcatgg cggcgcgacc tataccccgc tgatcggcaa agcgctgctg

721 agcgtgctgg gcgtgtatga ttggagcggc tgcaacccga tcccgccgga attctggctg

781 ctgccgagca gcttcccggt gaacggcggc accctgtgga tctatctgcg cgataccttc

841 atgggcctga gctatctgta tggcaaaaaa ttcgtggcgc cgccgacccc gctgatcctg

901 cagctgcgcg aagaactgta tccggaaccg tatgcgaaaa tcaactggac ccagacccgc

961 aaccgctgcg gcaaagaaga tctgtattat ccgcgcagct tcctgcagga tctgttctgg

1021 aaaagcgtgc acatgttcag cgaaagcatc ctggatcgct ggccgctgaa caaactgatc

1081 cgccagcgcg cgctgcagag caccatggcg ctgatccatt atcatgatga aagcacccgc

1141 tatatcaccg gcggctgcct gccgaaagcg ttccacatgc tggcgtgctg gatcgaagat

1201 ccgaaaagcg attatttcaa aaaacatctg gcgcgcgtgc gcgaatatat ctggatcggc

1261 gaagatggcc tgaaaatcca gagcttcggc agccagctgt gggataccgc gctgagcctg

1321 catgcgctgc tggatggcat cgatgatcat gatgtggatg atgaaatcaa aaccaccctg

1381 gtgaaaggct atgattatct gaaaaaaagc cagatcaccg aaaacccgcg cggcgatcat

1441 ttcaaaatgt tccgccataa aaccaaaggc ggctggacct tcagcgatca ggatcagggc

1501 tggccggtga gcgattgcac cgcggaaagc ctggaatgct gcctgttctt cgaaagcatg

1561 ccgagcgaac tgatcggcaa aaaaatggat gtggaaaaac tgtatgatgc ggtggattat

1621 ctgctgtatc tgcagagcga taacggcggc atcgcggcgt ggcagccggt ggaaggcaaa

1681 gcgtggctgg aactgctgaa catcatgatc ttccgctatg tggaatgcac cggcagcgcg

1741 atcgcggcgc tgacccagtt caacaaacag ttcccgggct ataaaaacgt ggaagtgaaa

1801 cgcttcatca ccaaagcggc gaaatatatc gaagatatgc agaccgtgga tggcagctgg

1861 tatggcaact ggggcgtgtg cttcatctat ggcaccttct ttgcggtgcg cggcctggtg

1921 gcggcgggca aaacctatag caactgcgaa gcgatccgca aagcggtgcg cttcctgctg

1981 gatacccaga acccggaagg cggctggggc gagagcttcc tgagctgccc gagcaaaaaa

2041 tataccccgc tgaaaggcaa cagcaccaac gtggtgcaga ccgcgcaggc gctgatggtg

2101 ctgatcatgg gcgatcagat ggaacgcgat ccgctgccgg tgcatcgcgc ggcgcaggtg

2161 ctgatcaaca gccagctgga taacggcgat ttcccgcagc aggaaatcat gggcaccttc

2221 atgcgcaccg tgatgctgca tttcccgacc tatcgcaaca ccttcagcct gtgggcgctg

2281 acccattata cccatgcgct gcgccgcctg ctgccgtaag cctttgacaa ggaattgaca

2341 tatgggcagc ctgggcacca tgctgcgcta tccggatgat atctatccgc tgctgaaaat

2401 gaaacgcgcg atcgaaaaag cggaaaaaca gatcccgccg gaaccgcatt ggggcttctg

2461 ctatagcatg ctgcataaag tgagccgcag cttcagcctg gtgatccagc agctgaacac

2521 cgaactgcgc aacgcggtgt gcgtgttcta tctggtgctg cgcgcgctgg ataccgtgga

2581 agatgatacc agcatcccga ccgatgaaaa agtgccgatc ctgatcgcgt tccatcgcca

2641 tatctatgat accgattggc attatagctg cggcaccaaa gaatataaaa tcctgatgga

2701 tcagttccat catgtgagcg cggcgttcct ggaactggaa aaaggctatc aggaagcgat

2761 cgaagaaatc acccgccgca tgggcgcggg catggcgaaa ttcatctgcc aggaagtgga

2821 aaccgtggat gattatgatg aatattgcca ttatgtggcg ggcctggtgg gcctgggcct

2881 gagcaaactg ttcctggcgg cgggcagcga agtgctgacc ccggattggg aagcgatcag

2941 caacagcatg ggcctgttcc tgcagaaaac caacatcatc cgcgattatc tggaagatat

3001 caacgaaatc ccgaaaagcc gcatgttctg gccgcgcgaa atctggggca aatatgcgga

3061 taaactggaa gatctgaaat atgaagaaaa caccaacaaa agcgtgcagt gcctgaacga

3121 aatggtgacc aacgcgctga tgcatatcga agattgcctg aaatatatgg tgagcctgcg

3181 cgatccgagc atcttccgct tctgcgcgat cccgcagatc atggcgatcg gcaccctggc

3241 gctgtgctat aacaacgaac aggtgttccg cggcgtggtg aaactgcgcc gcggcctgac

3301 cgcgaaagtg atcgatcgca ccaaaaccat ggcggatgtg tatggcgcgt tctatgattt

3361 cagctgcatg ctgaaaacca aagtggataa aaacgatccg aacgcgagca aaaccctgaa

3421 ccgcctggaa gcggtgcaga aactgtgccg cgatgcgggc gtgctgcaga accgcaaaag

3481 ctatgtgaac gataaaggcc agccgaacag cgtgttcatc atcatggtgg tgatcctgct

3541 ggcgatcgtg ttcgcgtatc tgcgcgcgaa ctaactcgag ataaggagat atacacatgg

3601 aaagccagct gtggaactgg atcctgccgc tgctgatcag cagcctgctg atcagcttcg

3661 tggcgttcta tggcttcttc gtgaaaccga aacgcaacgg cctgcgccat gatcgcaaaa

3721 ccgtgagcac cgtgaccagc gatgtgggca gcgtgaacat caccggcgat accgtggcgg

3781 atgtgatcgt ggtgggcgcg ggcgtggcgg gcagcgcgct ggcgtatacc ctgggcaaag

3841 ataaacgccg cgtgcatgtg atcgaacgcg atctgagcga accggatcgc atcgtgggcg

3901 aactgctgca gccgggcggc tatctgaaac tgctggaact gggcatcgaa gattgcgtgg

3961 aagaaatcga tgcgcagcgc gtgtatggct atgcgctgtt caaaaacggc aaacgcatcc

4021 gcctggcgta tccgctggaa aaattccatg aagatgtgag cggccgcagc ttccataacg

4081 gccgcttcat ccagcgcatg cgcgaaaaag cggcgagcct gccgaacgtg cagctggaac

4141 agggcaccgt gctgagcctg ctggaagaaa acggcaccat caaaggcgtg cgctataaaa

4201 acaaagcggg cgaagaacag accgcgttcg cggcgctgac catcgtgtgc gatggctgct

4261 tcagcaacct gcgccgcagc ctgtgcaacc cgcaggtgga agtgccgagc tgcttcgtgg

4321 gcctggtgct ggaaaactgc aacctgccgt atgcgaacca tggccatgtg gtgctggcgg

4381 atccgagccc gatcctgatg tatccgatca gcagcaccga agtgcgctgc ctggtggatg

4441 tgccgggcca gaaagtgccg agcatcgcga acggcgaaat gaaaaactat ctgaaaaccg

4501 tggtggcgcc gcagatgccg catgaagtgt atgatagctt catcgcggcg gtggataaag

4561 gcaacatcaa aagcatgccg aaccgcagca tgccggcgag cccgtatccg accccgggcg

4621 cgctgctgat gggcgatgcg ttcaacatgc gccatccgct gaccggcggc ggcatgaccg

4681 tggcgctggc ggatatcgtg gtgctgcgca acctgctgcg cccgctgcgc gatctgagcg

4741 atggcgcgag cctgtgcaaa tatctggaga gcttctatac cctgcgcaaa ccggtggcgg

4801 cgaccatcaa caccctggcg aacgcgctgt atcaggtgtt ctgcagcagc gaaaacgaag

4861 cgcgcaacga aatgcgcgaa gcgtgcttcg attatctggg cctgggcggc atgtgcacca

4921 gcggcccggt gagcctgctg agcggcctga acccgcgccc gctgaccctg gtgtgccatt

4981 tcttcgcggt ggcggtgtat ggcgtgatcc gcctgctgat cccgttcccg agcccgaaac

5041 gcatctggct gggcgcgaaa ctgatcagcg gcgcgagcgg catcatcttc ccgatcatca

5101 aagcggaagg cgtgcgccag atgttcttcc cggcgaccgt gccggcgtat tattataaag

5161 cgccgaccgt gggcgaaacc aaatgcagct aaacgcgtta taagcttgcg gccgcactcg

5221 agcaccacca ccaccaccac tgagatccgg ctgctaacaa agcccgaaag gaagctgagt

5281 tggctgctgc caccgctgag caataactag cataacccct tggggcctct aaacgggtct

5341 tgaggggttt tttg

//

**pRhon5Hi-2-MRN1-SQS1-SQE1**

LOCUS MRN1-SQS1-SQE1 5363 bp DNA linear SYN 24-OCT-2017

KEYWORDS pRhon5Hi-2-MRN1-SQS1-SQE1

FEATURES Location/Qualifiers

regulatory 29..34

/regulatory_class=ribosome_binding_site

/gene="pRhotHi-2 RBS"

/product="RBS of vector pRhotHi-2"

CDS 43..2328

/gene="MRN1"

/product="A. thaliana maneral synthase MRN1"

/translation="MWRLRIGAEARQDPHLFTTNNFAGRQIWEFDANGGSPEELAEVE

EARLNFANNKSRFKASPDLFWRRQFLREKKFEQKIPRVRIEDAEKITYEDAKTALRRG

VLYYAACQANDGHWPSEVSGSMFLDAPFVICLYITGHLEKIFTLEHVKELLRYMYNTQ

NEDGGWGLDVESHSVMFCTVLNYICLRILGVEPDHDGQKSACARARKWILDHGGATYA

PMVAKAWLSVLGVYDWSGCKPLPPEIWMLPSFSPINGGTLWIYIRDLLMGMSYLYGKK

FVATPTALILQLREELYPQPYSKIIWSKARNRCAKEDLLYPKSFGQDLFWEGVHMLSE

NIINRWPLNKFVRQRALRTTMELVHYHDETTHYITGACVAKPFHMLACWVEDPDGDYF

KKHLARVPDFIWIAEDGLKFQLMGMQSWNAALSLQVMLAANMDDEIRSTLIKGYDFLK

QSQISENPQGDHLKMFRDITKGGWTFQDREQGLPISDGTAESIECCIHFHRMPSEFIG

EKMDVEKLYDAVNFLIYLQSDNGGMPVWEPAPGKKWLEWLSPVEHVENTVVEQEYLEC

TGSVIAGLVCFKKEFPDHRPKEIEKLIKKGLKYIEDLQMPDGSWYGNWGVCFTYGTLF

AVRGLAAAGKTFGNSEAIRRAVQFILNTQNAEGGWGESALSCPNKKYIPSKGNVTNVV

NTGQAMMVLLIGGQMERDPSPVHRAAKVLINSQLDIGDFPQQERRGIYMNMLLHYPTY

RNMFSLWALALYTNALRLLVS"

regulatory 2329..2347

/regulatory_class=ribosome_binding_site

/gene="nifK RBS"

/product="5'UTR of R. capsulatus nifK incl RBS"

CDS 2351..3583

/gene="SQS1"

/product="A. thaliana squalene synthase SQS1"

/translation="MGSLGTMLRYPDDIYPLLKMKRAIEKAEKQIPPEPHWGFCYSML

HKVSRSFSLVIQQLNTELRNAVCVFYLVLRALDTVEDDTSIPTDEKVPILIAFHRHIY

DTDWHYSCGTKEYKILMDQFHHVSAAFLELEKGYQEAIEEITRRMGAGMAKFICQEVE

TVDDYDEYCHYVAGLVGLGLSKLFLAAGSEVLTPDWEAISNSMGLFLQKTNIIRDYLE

DINEIPKSRMFWPREIWGKYADKLEDLKYEENTNKSVQCLNEMVTNALMHIEDCLKYM

VSLRDPSIFRFCAIPQIMAIGTLALCYNNEQVFRGVVKLRRGLTAKVIDRTKTMADVY

GAFYDFSCMLKTKVDKNDPNASKTLNRLEAVQKLCRDAGVLQNRKSYVNDKGQPNSVF

IIMVVILLAIVFAYLRAN"

regulatory 3592..3597

/regulatory_class=ribosome_binding_site

/gene="pET RBS"

/product="RBS of pET vector series"

CDS 3606..5201

/gene="SQE1"

/product="A. thaliana squalene epoxidase SQE1"

/translation="MESQLWNWILPLLISSLLISFVAFYGFFVKPKRNGLRHDRKTVS

TVTSDVGSVNITGDTVADVIVVGAGVAGSALAYTLGKDKRRVHVIERDLSEPDRIVGE

LLQPGGYLKLLELGIEDCVEEIDAQRVYGYALFKNGKRIRLAYPLEKFHEDVSGRSFH

NGRFIQRMREKAASLPNVQLEQGTVLSLLEENGTIKGVRYKNKAGEEQTAFAALTIVC

DGCFSNLRRSLCNPQVEVPSCFVGLVLENCNLPYANHGHVVLADPSPILMYPISSTEV

RCLVDVPGQKVPSIANGEMKNYLKTVVAPQMPHEVYDSFIAAVDKGNIKSMPNRSMPA

SPYPTPGALLMGDAFNMRHPLTGGGMTVALADIVVLRNLLRPLRDLSDGASLCKYLES

FYTLRKPVAATINTLANALYQVFCSSENEARNEMREACFDYLGLGGMCTSGPVSLLSG

LNPRPLTLVCHFFAVAVYGVIRLLIPFPSPKRIWLGAKLISGASGIIFPIIKAEGVRQ

MFFPATVPAYYYKAPTVGETKCS"

regulatory 5316..5363

/regulatory_class=terminator

/gene="T7 terminator"

/product="bacteriophage T7 transcription terminator"

ORIGIN

1 tctagaaata attttgttta actttaagaa ggagatatac atatgtggcg cctgcgcatc

61 ggcgcggaag cgcgccagga tccgcatctg ttcaccacca acaacttcgc gggccgccag

121 atctgggaat tcgatgcgaa cggcggcagc ccggaagaac tggcggaagt ggaagaagcg

181 cgcctgaact tcgcgaacaa caaaagccgc ttcaaagcga gcccggatct gttctggcgc

241 cgccagttcc tgcgcgaaaa aaaattcgaa cagaaaatcc cgcgcgtgcg catcgaagat

301 gcggaaaaaa tcacctatga agatgcgaaa accgcgctgc gccgcggcgt gctgtattat

361 gcggcgtgcc aggcgaacga tggccattgg ccgagcgaag tgagcggcag catgttcctg

421 gatgcgccgt tcgtgatctg cctgtatatc accggccatc tggaaaaaat cttcaccctg

481 gaacatgtga aagaactgct gcgctatatg tataacaccc agaacgaaga tggcggctgg

541 ggcctggatg tggaaagcca tagcgtgatg ttctgcaccg tgctgaacta tatctgcctg

601 cgcatcctgg gcgtggaacc ggatcatgat ggccagaaaa gcgcgtgcgc gcgcgcgcgc

661 aaatggatcc tggatcatgg cggcgcgacc tatgcgccga tggtggcgaa agcgtggctg

721 agcgtgctgg gcgtgtatga ttggagcggc tgcaaaccgc tgccgccgga aatctggatg

781 ctgccgagct tcagcccgat caacggcggc accctgtgga tctatatccg cgatctgctg

841 atgggcatga gctatctgta tggcaagaaa ttcgtggcga ccccgaccgc gctgatcctg

901 cagctgcgcg aagaactgta tccgcagccg tatagcaaaa tcatctggag caaagcgcgc

961 aaccgctgcg cgaaagaaga tctgctgtat ccgaagagct tcggccagga tctgttctgg

1021 gaaggcgtgc acatgctgag cgaaaacatc atcaaccgct ggccgctgaa caaattcgtg

1081 cgccagcgcg cgctgcgcac caccatggaa ctggtgcatt atcatgatga aaccacccat

1141 tatatcaccg gcgcgtgcgt ggcgaaaccg ttccacatgc tggcgtgctg ggtggaagat

1201 ccggatggcg attatttcaa gaaacatctg gcgcgcgtgc cggatttcat ctggatcgcg

1261 gaagatggcc tgaaattcca gctgatgggc atgcagagct ggaacgcggc gctgagcctg

1321 caggtgatgc tggcggcgaa catggatgat gaaatccgca gcaccctgat caaaggctat

1381 gatttcctga aacagagcca gatcagcgaa aacccgcagg gcgatcatct gaaaatgttc

1441 cgcgatatca ccaaaggcgg ctggaccttc caggatcgcg aacagggcct gccgatcagc

1501 gatggcaccg cggaaagcat cgaatgctgc atccatttcc atcgcatgcc gagcgaattc

1561 atcggcgaaa aaatggatgt ggaaaaactg tatgatgcgg tgaacttcct gatctatctg

1621 cagagcgata acggcggcat gccggtgtgg gaaccggcgc cgggcaagaa atggctggaa

1681 tggctgagcc cggtggaaca tgtggaaaac accgtggtgg aacaggaata tctggaatgc

1741 accggcagcg tgatcgcggg cctggtgtgc ttcaagaaag aattcccgga tcatcgcccg

1801 aaagaaatcg aaaaactgat caagaaaggc ctgaaatata tcgaagatct gcagatgccg

1861 gatggcagct ggtatggcaa ctggggcgtg tgcttcacct atggcaccct gtttgcggtg

1921 cgcggcctgg cggcggcggg caaaaccttc ggcaacagcg aagcgatccg ccgcgcggtg

1981 cagttcatcc tgaacaccca gaacgcggaa ggcggctggg gcgaaagcgc gctgagctgc

2041 ccgaacaaga aatatatccc gagcaaaggc aacgtgacca acgtggtgaa caccggccag

2101 gcgatgatgg tgctgctgat cggcggccag atggaacgcg atccgagccc ggtgcatcgc

2161 gcggcgaaag tgctgatcaa cagccagctg gatatcggcg atttcccgca gcaggaacgc

2221 cgcggcatct atatgaacat gctgctgcat tatccgacct atcgcaacat gttcagcctg

2281 tgggcgctgg cgctgtatac caacgcgctg cgcctgctgg tgagctaagc ctttgacaag

2341 gaattgacat atgggcagcc tgggcaccat gctgcgctat ccggatgata tctatccgct

2401 gctgaaaatg aaacgcgcga tcgaaaaagc ggaaaaacag atcccgccgg aaccgcattg

2461 gggcttctgc tatagcatgc tgcataaagt gagccgcagc ttcagcctgg tgatccagca

2521 gctgaacacc gaactgcgca acgcggtgtg cgtgttctat ctggtgctgc gcgcgctgga

2581 taccgtggaa gatgatacca gcatcccgac cgatgaaaaa gtgccgatcc tgatcgcgtt

2641 ccatcgccat atctatgata ccgattggca ttatagctgc ggcaccaaag aatataaaat

2701 cctgatggat cagttccatc atgtgagcgc ggcgttcctg gaactggaaa aaggctatca

2761 ggaagcgatc gaagaaatca cccgccgcat gggcgcgggc atggcgaaat tcatctgcca

2821 ggaagtggaa accgtggatg attatgatga atattgccat tatgtggcgg gcctggtggg

2881 cctgggcctg agcaaactgt tcctggcggc gggcagcgaa gtgctgaccc cggattggga

2941 agcgatcagc aacagcatgg gcctgttcct gcagaaaacc aacatcatcc gcgattatct

3001 ggaagatatc aacgaaatcc cgaaaagccg catgttctgg ccgcgcgaaa tctggggcaa

3061 atatgcggat aaactggaag atctgaaata tgaagaaaac accaacaaaa gcgtgcagtg

3121 cctgaacgaa atggtgacca acgcgctgat gcatatcgaa gattgcctga aatatatggt

3181 gagcctgcgc gatccgagca tcttccgctt ctgcgcgatc ccgcagatca tggcgatcgg

3241 caccctggcg ctgtgctata acaacgaaca ggtgttccgc ggcgtggtga aactgcgccg

3301 cggcctgacc gcgaaagtga tcgatcgcac caaaaccatg gcggatgtgt atggcgcgtt

3361 ctatgatttc agctgcatgc tgaaaaccaa agtggataaa aacgatccga acgcgagcaa

3421 aaccctgaac cgcctggaag cggtgcagaa actgtgccgc gatgcgggcg tgctgcagaa

3481 ccgcaaaagc tatgtgaacg ataaaggcca gccgaacagc gtgttcatca tcatggtggt

3541 gatcctgctg gcgatcgtgt tcgcgtatct gcgcgcgaac taactcgaga taaggagata

3601 tacacatgga aagccagctg tggaactgga tcctgccgct gctgatcagc agcctgctga

3661 tcagcttcgt ggcgttctat ggcttcttcg tgaaaccgaa acgcaacggc ctgcgccatg

3721 atcgcaaaac cgtgagcacc gtgaccagcg atgtgggcag cgtgaacatc accggcgata

3781 ccgtggcgga tgtgatcgtg gtgggcgcgg gcgtggcggg cagcgcgctg gcgtataccc

3841 tgggcaaaga taaacgccgc gtgcatgtga tcgaacgcga tctgagcgaa ccggatcgca

3901 tcgtgggcga actgctgcag ccgggcggct atctgaaact gctggaactg ggcatcgaag

3961 attgcgtgga agaaatcgat gcgcagcgcg tgtatggcta tgcgctgttc aaaaacggca

4021 aacgcatccg cctggcgtat ccgctggaaa aattccatga agatgtgagc ggccgcagct

4081 tccataacgg ccgcttcatc cagcgcatgc gcgaaaaagc ggcgagcctg ccgaacgtgc

4141 agctggaaca gggcaccgtg ctgagcctgc tggaagaaaa cggcaccatc aaaggcgtgc

4201 gctataaaaa caaagcgggc gaagaacaga ccgcgttcgc ggcgctgacc atcgtgtgcg

4261 atggctgctt cagcaacctg cgccgcagcc tgtgcaaccc gcaggtggaa gtgccgagct

4321 gcttcgtggg cctggtgctg gaaaactgca acctgccgta tgcgaaccat ggccatgtgg

4381 tgctggcgga tccgagcccg atcctgatgt atccgatcag cagcaccgaa gtgcgctgcc

4441 tggtggatgt gccgggccag aaagtgccga gcatcgcgaa cggcgaaatg aaaaactatc

4501 tgaaaaccgt ggtggcgccg cagatgccgc atgaagtgta tgatagcttc atcgcggcgg

4561 tggataaagg caacatcaaa agcatgccga accgcagcat gccggcgagc ccgtatccga

4621 ccccgggcgc gctgctgatg ggcgatgcgt tcaacatgcg ccatccgctg accggcggcg

4681 gcatgaccgt ggcgctggcg gatatcgtgg tgctgcgcaa cctgctgcgc ccgctgcgcg

4741 atctgagcga tggcgcgagc ctgtgcaaat atctggagag cttctatacc ctgcgcaaac

4801 cggtggcggc gaccatcaac accctggcga acgcgctgta tcaggtgttc tgcagcagcg

4861 aaaacgaagc gcgcaacgaa atgcgcgaag cgtgcttcga ttatctgggc ctgggcggca

4921 tgtgcaccag cggcccggtg agcctgctga gcggcctgaa cccgcgcccg ctgaccctgg

4981 tgtgccattt cttcgcggtg gcggtgtatg gcgtgatccg cctgctgatc ccgttcccga

5041 gcccgaaacg catctggctg ggcgcgaaac tgatcagcgg cgcgagcggc atcatcttcc

5101 cgatcatcaa agcggaaggc gtgcgccaga tgttcttccc ggcgaccgtg ccggcgtatt

5161 attataaagc gccgaccgtg ggcgaaacca aatgcagcta aacgcgttat aagcttgcgg

5221 ccgcactcga gcaccaccac caccaccact gagatccggc tgctaacaaa gcccgaaagg

5281 aagctgagtt ggctgctgcc accgctgagc aataactagc ataacccctt ggggcctcta

5341 aacgggtctt gaggggtttt ttg

//

**Inserts in pVZ-based vectors for expression in *Synechocystis***

**pVZ-PcoaT-SQE1**

LOCUS PcoaT-SQE1 2925 bp DNA linear UNA 18-OCT-2017

KEYWORDS pVZ-PcoaT-SQE1

FEATURES Location/Qualifiers

source 1..2925

/organism="unspecified"

/mol_type="genomic DNA"

misc_feature 1..6

/label=PstI/NsiI scar

misc_feature 7..28

/label=BioBrick prefix

/note="BioBrick prefix for parts that do not start with

"ATG"

CDS complement(29..1142)

/codon_start=1

/gene=coaR

/label=coaR

/locus_tag=MYO_127660

/product=MerR family transcriptional regulator

/protein_id=AGF52994

/note=cobalt-dependent transcriptional regulator from Synechocystis sp. PCC 6803; similar to sll0794

/translation="MKTNHLTIKELTDAVGGGVTPRMVRHYHTLGLLPPVQRSEGNYRL

YTQQDVQRLQRVIALKQQGFQLSHIRQLLDSHSEESLDPTLMVQLQQQYQAVIQQITRL

RQTASALEGLLGRDQSCQITQAEALAQLKQLDVDVQEGLGKLDQLWTNLDAETTTHPEA

FQESLKHLLPDLSAYSEITIHLLHQLVLACGDVSLVNAVRLSQGAIASARDALKAGCPV

VTDVPVVAAALDQTRLAHLGCTVKTLIDDPHITGLREAEQAFWHHDHWQQRLQQIPQGC

VLAIGYAPSVLLTACKLIEQQHIQPALVIGMPIGFSHAPGAKRRLMTSPIPHITIQGSL

GGGLLAAVTLNALVETLIAKPDCHCYLTCL"

misc_feature 1143..1223

/label=PcoaT+5'UTR

/note=promoter including 5'UTR of coaT gene from Synechocystis sp. PCC 6803 (Moscow wildtype)

CDS 1224..2819

/codon_start=1

/gene=SQE1

/label=SQE1

/product="A. thaliana squalene epoxidase SQE1"

/note="second amino acid changed from changed from Glu to Ala"

/translation="MASQLWNWILPLLISSLLISFVAFYGFFVKPKRNGLRHDRKTVST

VTSDVGSVNITGDTVADVIVVGAGVAGSALAYTLGKDKRRVHVIERDLSEPDRIVGELL

QPGGYLKLLELGIEDCVEEIDAQRVYGYALFKNGKRIRLAYPLEKFHEDVSGRSFHNGR

FIQRMREKAASLPNVQLEQGTVLSLLEENGTIKGVRYKNKAGEEQTAFAALTIVCDGCF

SNLRRSLCNPQVEVPSCFVGLVLENCNLPYANHGHVVLADPSPILMYPISSTEVRCLVD

VPGQKVPSIANGEMKNYLKTVVAPQMPHEVYDSFIAAVDKGNIKSMPNRSMPASPYPTP

GALLMGDAFNMRHPLTGGGMTVALADIVVLRNLLRPLRDLSDGASLCKYLESFYTLRKP

VAATINTLANALYQVFCSSENEARNEMREACFDYLGLGGMCTSGPVSLLSGLNPRPLTL

VCHFFAVAVYGVIRLLIPFPSPKRIWLGAKLISGASGIIFPIIKAEGVRQMFFPATVPA

YYYKAPTVGETKCS"

terminator 2849..2896

/label=T7 terminator

/note="transcription terminator for bacteriophage T7 RNA

polymerase"

misc_feature 2902..2920

/label=BioBrick Suffix*

ORIGIN

1 ctgcatgaat tcgcggccgc ttctagagac taaagacaag tgagatagca gtggcaatct

61 ggctttgcaa tcaatgtttc cactaaagcg tttagcgtta ctgcggctag aagtcctcca

121 ccgaggctcc cctgaatggt gatatgggga atgggactgg tcatcagtcg tcgttttgcc

181 cccggagcat gactaaaacc gatcggcatt ccgatcacaa gagccggctg aatatgttgt

241 tgctctatca gcttacaggc agtgagtaaa acagaagggg catagccgat cgccagcaca

301 catccttggg gaatctgttg taaccgctgt tgccaatggt catggtgcca aaaagcttgc

361 tcggcttccc taagccctgt gatgtgaggg tcgtcaatca gcgttttaac cgtacatcct

421 aaatgagcta accgagtttg atcaagagcc gcagccacaa ccggaacatc ggtgacgact

481 ggacaccctg ctttcagtgc atctcgtgcc gaggcgatcg ctccctgact caatcgaacg

541 gcgtttacca agctaacatc accacaggcc agcactaatt gatgtagtaa gtgaatggta

601 atttcagagt aagccgataa atccggtagc aggtgtttga gggattcctg aaaggcttct

661 ggatgagttg ttgtctccgc atctaggttc gtccacaact gatcgagttt tcctaacccc

721 tcctggacat ccacatcaag ctgtttcagt tgggccagag cttccgcttg ggtaatctgg

781 caactctggt cgcgtcccag taatccttct aaagcagatg cggtttggcg gagtcgagta

841 atctgctgaa tcacagcctg atattgctgt tgcaactgca ccattagggt gggatcaagg

901 ctctcttcag aatggctatc cagcagttgc cgaatatgag acaactgaaa gccctgctgt

961 ttgagggcaa tgactcgttg gagccgttgt acgtcctgct gagtataaag gcggtagttg

1021 ccctctgagc gttgaacggg gggaagcaat cccagggtgt ggtaatggcg caccatgcga

1081 ggcgtaacgc cacctcccac tgcatctgtg agttctttaa tcgttaagtg attagtcttc

1141 atccctttag tttactcaaa accttgacat tgacactaat gttaaggttt aggctgagaa

1201 ggtaaaaatc caagttaaaa agcatggcta gccagctgtg gaactggatc ctgccgctgc

1261 tgatcagcag cctgctgatc agcttcgtgg cgttctatgg cttcttcgtg aaaccgaaac

1321 gcaacggcct gcgccatgat cgcaaaaccg tgagcaccgt gaccagcgat gtgggcagcg

1381 tgaacatcac cggcgatacc gtggcggatg tgatcgtggt gggcgcgggc gtggcgggca

1441 gcgcgctggc gtataccctg ggcaaagata aacgccgcgt gcatgtgatc gaacgcgatc

1501 tgagcgaacc ggatcgcatc gtgggcgaac tgctgcagcc gggcggctat ctgaaactgc

1561 tggaactggg catcgaagat tgcgtggaag aaatcgatgc gcagcgcgtg tatggctatg

1621 cgctgttcaa aaacggcaaa cgcatccgcc tggcgtatcc gctggaaaaa ttccatgaag

1681 atgtgagcgg ccgcagcttc cataacggcc gcttcatcca gcgcatgcgc gaaaaagcgg

1741 cgagcctgcc gaacgtgcag ctggaacagg gcaccgtgct gagcctgctg gaagaaaacg

1801 gcaccatcaa aggcgtgcgc tataaaaaca aagcgggcga agaacagacc gcgttcgcgg

1861 cgctgaccat cgtgtgcgat ggctgcttca gcaacctgcg ccgcagcctg tgcaacccgc

1921 aggtggaagt gccgagctgc ttcgtgggcc tggtgctgga aaactgcaac ctgccgtatg

1981 cgaaccatgg ccatgtggtg ctggcggatc cgagcccgat cctgatgtat ccgatcagca

2041 gcaccgaagt gcgctgcctg gtggatgtgc cgggccagaa agtgccgagc atcgcgaacg

2101 gcgaaatgaa aaactatctg aaaaccgtgg tggcgccgca gatgccgcat gaagtgtatg

2161 atagcttcat cgcggcggtg gataaaggca acatcaaaag catgccgaac cgcagcatgc

2221 cggcgagccc gtatccgacc ccgggcgcgc tgctgatggg cgatgcgttc aacatgcgcc

2281 atccgctgac cggcggcggc atgaccgtgg cgctggcgga tatcgtggtg ctgcgcaacc

2341 tgctgcgccc gctgcgcgat ctgagcgatg gcgcgagcct gtgcaaatat ctggagagct

2401 tctataccct gcgcaaaccg gtggcggcga ccatcaacac cctggcgaac gcgctgtatc

2461 aggtgttctg cagcagcgaa aacgaagcgc gcaacgaaat gcgcgaagcg tgcttcgatt

2521 atctgggcct gggcggcatg tgcaccagcg gcccggtgag cctgctgagc ggcctgaacc

2581 cgcgcccgct gaccctggtg tgccatttct tcgcggtggc ggtgtatggc gtgatccgcc

2641 tgctgatccc gttcccgagc ccgaaacgca tctggctggg cgcgaaactg atcagcggcg

2701 cgagcggcat catcttcccg atcatcaaag cggaaggcgt gcgccagatg ttcttcccgg

2761 cgaccgtgcc ggcgtattat tataaagcgc cgaccgtggg cgaaaccaaa tgcagctaac

2821 ctaggctgct gccaccgctg agcaataact agcataaccc cttggggcct ctaaacgggt

2881 cttgaggggt tttttgggcg actagtagcg gccgctgcag tcgac

//

**pVZ-PcoaT-SQE1-CAS1**

LOCUS PcoaT-SQE1-CAS1 5332 bp ds-DNA linear UNA 18-OCT-2017

KEYWORDS pVZ-PcoaT-SQE1-CAS1

FEATURES Location/Qualifiers

source 1..5332

/organism="unspecified"

/mol_type="genomic DNA"

source 1..5327

/organism="unspecified"

/mol_type="genomic DNA"

misc_feature 1..6

/label=PstI/NsiI scar

misc_feature 7..28

/label=BioBrick prefix

/note="BioBrick prefix for parts that do not start with

""ATG"

CDS complement(29..1142)

/codon_start=1

/gene="coaR"

/locus_tag="MYO_127660"

/product="MerR family transcriptional regulator"

/label=coaR

/note="cobalt-dependent transcriptional regulator from

Synechocystis sp. PCC 6803; similar to sll0794"

/protein_id="AGF52994"

/translation="MKTNHLTIKELTDAVGGGVTPRMVRHYHTLGLLPPVQRSEGNYRL

YTQQDVQRLQRVIALKQQGFQLSHIRQLLDSHSEESLDPTLMVQLQQQYQAVIQQITRL

RQTASALEGLLGRDQSCQITQAEALAQLKQLDVDVQEGLGKLDQLWTNLDAETTTHPEA

FQESLKHLLPDLSAYSEITIHLLHQLVLACGDVSLVNAVRLSQGAIASARDALKAGCPV

VTDVPVVAAALDQTRLAHLGCTVKTLIDDPHITGLREAEQAFWHHDHWQQRLQQIPQGC

VLAIGYAPSVLLTACKLIEQQHIQPALVIGMPIGFSHAPGAKRRLMTSPIPHITIQGSL

GGGLLAAVTLNALVETLIAKPDCHCYLTCL"

misc_feature 1143..1223

/label=PcoaT+5'UTR

/note="promoter including 5'UTR of coaT gene from

Synechocystis sp. PCC 6803 (Moscow wildtype)"

CDS 1224..2819

/codon_start=1

/gene="SQE1"

/product="A. thaliana squalene epoxidase SQE1"

/label=SQE1

/note="second amino acid changed from changed from Glu to

Ala"

/translation="MASQLWNWILPLLISSLLISFVAFYGFFVKPKRNGLRHDRKTVST

VTSDVGSVNITGDTVADVIVVGAGVAGSALAYTLGKDKRRVHVIERDLSEPDRIVGELL

QPGGYLKLLELGIEDCVEEIDAQRVYGYALFKNGKRIRLAYPLEKFHEDVSGRSFHNGR

FIQRMREKAASLPNVQLEQGTVLSLLEENGTIKGVRYKNKAGEEQTAFAALTIVCDGCF

SNLRRSLCNPQVEVPSCFVGLVLENCNLPYANHGHVVLADPSPILMYPISSTEVRCLVD

VPGQKVPSIANGEMKNYLKTVVAPQMPHEVYDSFIAAVDKGNIKSMPNRSMPASPYPTP

GALLMGDAFNMRHPLTGGGMTVALADIVVLRNLLRPLRDLSDGASLCKYLESFYTLRKP

VAATINTLANALYQVFCSSENEARNEMREACFDYLGLGGMCTSGPVSLLSGLNPRPLTL

VCHFFAVAVYGVIRLLIPFPSPKRIWLGAKLISGASGIIFPIIKAEGVRQMFFPATVPA

YYYKAPTVGETKCS"

RBS 2827..2838

/label=BBa_0034

CDS 2845..5121

/codon_start=1

/gene="CAS1"

/product="A. thaliana cycloartenol synthase CAS1"

/label=CAS1

/translation="MWKLKIAEGGSPWLRTTNNHVGRQFWEFDPNLGTPEDLAAVEEAR

KSFSDNRFVQKHSADLLMRLQFSRENLISPVLPQVKIEDTDDVTEEMVETTLKRGLDFY

STIQAHDGHWPGDYGGPMFLLPGLIITLSITGALNTVLSEQHKQEMRRYLYNHQNEDGG

WGLHIEGPSTMFGSVLNYVTLRLLGEGPNDGDGDMEKGRDWILNHGGATNITSWGKMWL

SVLGAFEWSGNNPLPPEIWLLPYFLPIHPGRMWCHCRMVYLPMSYLYGKRFVGPITSTV

LSLRKELFTVPYHEVNWNEARNLCAKEDLYYPHPLVQDILWASLHKIVEPVLMRWPGAN

LREKAIRTAIEHIHYEDENTRYICIGPVNKVLNMLCCWVEDPNSEAFKLHLPRIHDFLW

LAEDGMKMQGYNGSQLWDTGFAIQAILATNLVEEYGPVLEKAHSFVKNSQVLEDCPGDL

NYWYRHISKGAWPFSTADHGWPISDCTAEGLKAALLLSKVPKAIVGEPIDAKRLYEAVN

VIISLQNADGGLATYELTRSYPWLELINPAETFGDIVIDYPYVECTSAAIQALISFRKL

YPGHRKKEVDECIEKAVKFIESIQAADGSWYGSWAVCFTYGTWFGVKGLVAVGKTLKNS

PHVAKACEFLLSKQQPSGGWGESYLSCQDKVYSNLDGNRSHVVNTAWAMLALIGAGQAE

VDRKPLHRAARYLINAQMENGDFPQQEIMGVFNRNCMITYAAYRNIFPIWALGEYRCQV

LLQQGE"

CDS 5122..5124

/codon_start=1

/label=STOP

/note="second stop codon"

/translation=""

CDS 5146..5163

/codon_start=1

/product="6xHis affinity tag"

/label=6xHis

/note="not translated in this construct"

/translation="HHHHHH"

terminator 5230..5277

/label=T7 terminator

/note="transcription terminator for bacteriophage T7 RNA

polymerase"

misc_feature 5308..5327

/label=BioBrick Suffix*

/note="lacking 5'terminal T"

ORIGIN

1 ctgcatgaat tcgcggccgc ttctagagac taaagacaag tgagatagca gtggcaatct

61 ggctttgcaa tcaatgtttc cactaaagcg tttagcgtta ctgcggctag aagtcctcca

121 ccgaggctcc cctgaatggt gatatgggga atgggactgg tcatcagtcg tcgttttgcc

181 cccggagcat gactaaaacc gatcggcatt ccgatcacaa gagccggctg aatatgttgt

241 tgctctatca gcttacaggc agtgagtaaa acagaagggg catagccgat cgccagcaca

301 catccttggg gaatctgttg taaccgctgt tgccaatggt catggtgcca aaaagcttgc

361 tcggcttccc taagccctgt gatgtgaggg tcgtcaatca gcgttttaac cgtacatcct

421 aaatgagcta accgagtttg atcaagagcc gcagccacaa ccggaacatc ggtgacgact

481 ggacaccctg ctttcagtgc atctcgtgcc gaggcgatcg ctccctgact caatcgaacg

541 gcgtttacca agctaacatc accacaggcc agcactaatt gatgtagtaa gtgaatggta

601 atttcagagt aagccgataa atccggtagc aggtgtttga gggattcctg aaaggcttct

661 ggatgagttg ttgtctccgc atctaggttc gtccacaact gatcgagttt tcctaacccc

721 tcctggacat ccacatcaag ctgtttcagt tgggccagag cttccgcttg ggtaatctgg

781 caactctggt cgcgtcccag taatccttct aaagcagatg cggtttggcg gagtcgagta

841 atctgctgaa tcacagcctg atattgctgt tgcaactgca ccattagggt gggatcaagg

901 ctctcttcag aatggctatc cagcagttgc cgaatatgag acaactgaaa gccctgctgt

961 ttgagggcaa tgactcgttg gagccgttgt acgtcctgct gagtataaag gcggtagttg

1021 ccctctgagc gttgaacggg gggaagcaat cccagggtgt ggtaatggcg caccatgcga

1081 ggcgtaacgc cacctcccac tgcatctgtg agttctttaa tcgttaagtg attagtcttc

1141 atccctttag tttactcaaa accttgacat tgacactaat gttaaggttt aggctgagaa

1201 ggtaaaaatc caagttaaaa agcatggcta gccagctgtg gaactggatc ctgccgctgc

1261 tgatcagcag cctgctgatc agcttcgtgg cgttctatgg cttcttcgtg aaaccgaaac

1321 gcaacggcct gcgccatgat cgcaaaaccg tgagcaccgt gaccagcgat gtgggcagcg

1381 tgaacatcac cggcgatacc gtggcggatg tgatcgtggt gggcgcgggc gtggcgggca

1441 gcgcgctggc gtataccctg ggcaaagata aacgccgcgt gcatgtgatc gaacgcgatc

1501 tgagcgaacc ggatcgcatc gtgggcgaac tgctgcagcc gggcggctat ctgaaactgc

1561 tggaactggg catcgaagat tgcgtggaag aaatcgatgc gcagcgcgtg tatggctatg

1621 cgctgttcaa aaacggcaaa cgcatccgcc tggcgtatcc gctggaaaaa ttccatgaag

1681 atgtgagcgg ccgcagcttc cataacggcc gcttcatcca gcgcatgcgc gaaaaagcgg

1741 cgagcctgcc gaacgtgcag ctggaacagg gcaccgtgct gagcctgctg gaagaaaacg

1801 gcaccatcaa aggcgtgcgc tataaaaaca aagcgggcga agaacagacc gcgttcgcgg

1861 cgctgaccat cgtgtgcgat ggctgcttca gcaacctgcg ccgcagcctg tgcaacccgc

1921 aggtggaagt gccgagctgc ttcgtgggcc tggtgctgga aaactgcaac ctgccgtatg

1981 cgaaccatgg ccatgtggtg ctggcggatc cgagcccgat cctgatgtat ccgatcagca

2041 gcaccgaagt gcgctgcctg gtggatgtgc cgggccagaa agtgccgagc atcgcgaacg

2101 gcgaaatgaa aaactatctg aaaaccgtgg tggcgccgca gatgccgcat gaagtgtatg

2161 atagcttcat cgcggcggtg gataaaggca acatcaaaag catgccgaac cgcagcatgc

2221 cggcgagccc gtatccgacc ccgggcgcgc tgctgatggg cgatgcgttc aacatgcgcc

2281 atccgctgac cggcggcggc atgaccgtgg cgctggcgga tatcgtggtg ctgcgcaacc

2341 tgctgcgccc gctgcgcgat ctgagcgatg gcgcgagcct gtgcaaatat ctggagagct

2401 tctataccct gcgcaaaccg gtggcggcga ccatcaacac cctggcgaac gcgctgtatc

2461 aggtgttctg cagcagcgaa aacgaagcgc gcaacgaaat gcgcgaagcg tgcttcgatt

2521 atctgggcct gggcggcatg tgcaccagcg gcccggtgag cctgctgagc ggcctgaacc

2581 cgcgcccgct gaccctggtg tgccatttct tcgcggtggc ggtgtatggc gtgatccgcc

2641 tgctgatccc gttcccgagc ccgaaacgca tctggctggg cgcgaaactg atcagcggcg

2701 cgagcggcat catcttcccg atcatcaaag cggaaggcgt gcgccagatg ttcttcccgg

2761 cgaccgtgcc ggcgtattat tataaagcgc cgaccgtggg cgaaaccaaa tgcagctaaa

2821 cgcgtaaaag aggagaaata ctagatgtgg aaactgaaaa tcgcggaagg cggcagcccg

2881 tggctgcgca ccaccaacaa ccatgtgggc cgccagttct gggagttcga tccgaacctg

2941 ggcaccccgg aagatctggc ggcggtggaa gaagcgcgca agagcttcag cgataaccgc

3001 ttcgtgcaga aacatagcgc ggatctgctg atgcgcctgc agttcagccg cgaaaacctg

3061 atcagcccgg tgctgccgca ggtgaaaatc gaagataccg atgatgtgac cgaagaaatg

3121 gtggaaacca ccctgaaacg cggcctggat ttctatagca ccatccaggc gcatgatggc

3181 cattggccgg gcgattatgg cggcccgatg ttcctgctgc cgggcctgat catcaccctg

3241 agcatcaccg gcgcgctgaa caccgtgctg agcgaacagc ataaacagga aatgcgccgc

3301 tatctgtata accatcagaa cgaagatggc ggctggggcc tgcatatcga aggcccgagc

3361 accatgttcg gcagcgtgct gaactatgtg accctgcgcc tgctgggcga aggcccgaac

3421 gatggcgatg gcgatatgga aaaaggccgc gattggatcc tgaaccatgg cggcgcgacc

3481 aacatcacca gctggggcaa aatgtggctg agcgtgctgg gcgcgttcga atggagcggc

3541 aacaacccgc tgccgccgga aatctggctg ctgccgtatt tcctgccgat ccatccgggc

3601 cgcatgtggt gccattgccg catggtgtat ctgccgatga gctatctgta tggcaaacgc

3661 ttcgtgggcc cgatcaccag caccgtgctg agcctgcgca aagaactgtt caccgtgccg

3721 tatcatgaag tgaactggaa cgaagcgcgc aacctgtgcg cgaaagaaga tctgtattat

3781 ccgcatccgc tggtgcagga tatcctgtgg gcgagcctgc ataaaatcgt ggaaccggtg

3841 ctgatgcgct ggccgggcgc gaacctgcgc gaaaaagcga tccgcaccgc gatcgaacat

3901 atccattatg aagatgaaaa cacccgctat atctgcatcg gcccggtgaa caaagtgctg

3961 aacatgctgt gctgctgggt ggaagatccg aacagcgaag cgttcaaact gcatctgccg

4021 cgcatccatg atttcctgtg gctggcggaa gatggcatga aaatgcaggg ctataacggc

4081 agccagctgt gggataccgg cttcgcgatc caggcgatcc tggcgaccaa cctggtggaa

4141 gaatatggcc cggtgctgga aaaagcgcat agcttcgtga aaaacagcca ggtgctggaa

4201 gattgcccgg gcgatctgaa ctattggtat cgccatatca gcaaaggcgc gtggccgttc

4261 agcaccgcgg atcatggctg gccgatcagc gattgcaccg cggaaggcct gaaagcggcg

4321 ctgctgctga gcaaagtgcc gaaagcgatc gtgggcgaac cgatcgatgc gaaacgcctg

4381 tatgaagcgg tgaacgtgat catcagcctg cagaacgcgg atggcggcct ggcgacctat

4441 gaactgaccc gcagctatcc gtggctggaa ctgatcaacc cggcggaaac cttcggcgat

4501 atcgtgatcg attatccgta tgtggaatgc accagcgcgg cgatccaggc gctgatcagc

4561 ttccgcaaac tgtatccggg ccatcgcaaa aaagaagtgg atgaatgcat cgaaaaagcg

4621 gtgaaattca tcgaaagcat ccaggcggcg gatggcagct ggtatggcag ctgggcggtg

4681 tgcttcacct atggcacctg gttcggcgtg aaaggcctgg tggcggtggg caaaaccctg

4741 aaaaacagcc cgcatgtggc gaaagcgtgc gagttcctgc tgagcaaaca gcagccgagc

4801 ggcggctggg gcgaaagcta tctgagctgc caggataaag tgtatagcaa cctggatggc

4861 aaccgcagcc atgtggtgaa caccgcgtgg gcgatgctgg cgctgatcgg cgcgggccag

4921 gcggaagtgg atcgcaaacc gctgcatcgc gcggcgcgct atctgatcaa cgcgcagatg

4981 gaaaacggcg atttcccgca gcaggaaatc atgggcgtgt tcaaccgcaa ctgcatgatc

5041 acctatgcgg cgtatcgcaa catcttcccg atctgggcgc tgggcgaata tcgctgccag

5101 gtgctgctgc agcagggcga ataaaagctt gcggccgcac tcgagcacca ccaccaccac

5161 cactgagatc cggctgctaa caaagcccga aaggaagctg agttggctgc tgccaccgct

5221 gagcaataac tagcataacc ccttggggcc tctaaacggg tcttgagggg ttttttgctg

5281 aaaggaggaa ctatatccgg attggcgact agtagcggcc gctgcagtcg ac

//

**pVZ-PcoaT-SQE1-LUP1**

LOCUS PcoaT-SQE1-LUP1 5326 bp ds-DNA linear UNA 18-OCT-2017

KEYWORDS pVZ-PcoaT-SQE1-LUP1

FEATURES Location/Qualifiers

source 1..5326

/organism="unspecified"

/mol_type="genomic DNA"

misc_feature 1..6

/label=PstI/NsiI scar

misc_feature 7..28

/label=BioBrick prefix

/note="BioBrick prefix for parts that do not start with

"ATG"

CDS complement(29..1142)

/codon_start=1

/gene="coaR"

/locus_tag="MYO_127660"

/product="MerR family transcriptional regulator"

/label=coaR

/note="cobalt-dependent transcriptional regulator from

Synechocystis sp. PCC 6803; similar to sll0794"

/protein_id="AGF52994"

/translation="MKTNHLTIKELTDAVGGGVTPRMVRHYHTLGLLPPVQRSEGNYRL

YTQQDVQRLQRVIALKQQGFQLSHIRQLLDSHSEESLDPTLMVQLQQQYQAVIQQITRL

RQTASALEGLLGRDQSCQITQAEALAQLKQLDVDVQEGLGKLDQLWTNLDAETTTHPEA

FQESLKHLLPDLSAYSEITIHLLHQLVLACGDVSLVNAVRLSQGAIASARDALKAGCPV

VTDVPVVAAALDQTRLAHLGCTVKTLIDDPHITGLREAEQAFWHHDHWQQRLQQIPQGC

VLAIGYAPSVLLTACKLIEQQHIQPALVIGMPIGFSHAPGAKRRLMTSPIPHITIQGSL

GGGLLAAVTLNALVETLIAKPDCHCYLTCL"

misc_feature 1143..1223

/label=PcoaT+5'UTR

/note="promoter including 5'UTR of coaT gene from

Synechocystis sp. PCC 6803 (Moscow wildtype)"

CDS 1224..2819

/codon_start=1

/gene="SQE1"

/product="A. thaliana squalene epoxidase SQE1"

/label=SQE1

/note="second amino acid changed from changed from Glu to

Ala"

/translation="MASQLWNWILPLLISSLLISFVAFYGFFVKPKRNGLRHDRKTVST

VTSDVGSVNITGDTVADVIVVGAGVAGSALAYTLGKDKRRVHVIERDLSEPDRIVGELL

QPGGYLKLLELGIEDCVEEIDAQRVYGYALFKNGKRIRLAYPLEKFHEDVSGRSFHNGR

FIQRMREKAASLPNVQLEQGTVLSLLEENGTIKGVRYKNKAGEEQTAFAALTIVCDGCF

SNLRRSLCNPQVEVPSCFVGLVLENCNLPYANHGHVVLADPSPILMYPISSTEVRCLVD

VPGQKVPSIANGEMKNYLKTVVAPQMPHEVYDSFIAAVDKGNIKSMPNRSMPASPYPTP

GALLMGDAFNMRHPLTGGGMTVALADIVVLRNLLRPLRDLSDGASLCKYLESFYTLRKP

VAATINTLANALYQVFCSSENEARNEMREACFDYLGLGGMCTSGPVSLLSGLNPRPLTL

VCHFFAVAVYGVIRLLIPFPSPKRIWLGAKLISGASGIIFPIIKAEGVRQMFFPATVPA

YYYKAPTVGETKCS"

RBS 2827..2838

/label=BBa_0034

CDS 2845..5115

/codon_start=1

/gene="LUP1 "

/product="A. thaliana lupeol synthase LUP1"

/label=LUP1

/translation="MWKLKIGKGNGEDPHLFSSNNFVGRQTWKFDHKAGSPEERAAVEE

ARRGFLDNRFRVKGCSDLLWRMQFLREKKFEQGIPQLKATNIEEITYETTTNALRRGVR

YFTALQASDGHWPGEITGPLFFLPPLIFCLYITGHLEEVFDAEHRKEMLRHIYCHQNED

GGWGLHIESKSVMFCTVLNYICLRMLGENPEQDACKRARQWILDRGGVIFIPSWGKFWL

SILGVYDWSGTNPTPPELLMLPSFLPIHPGKILCYSRMVSIPMSYLYGKRFVGPITPLI

LLLREELYLEPYEEINWKKSRRLYAKEDMYYAHPLVQDLLSDTLQNFVEPLLTRWPLNK

LVREKALQLTMKHIHYEDENSHYITIGCVEKVLCMLACWVENPNGDYFKKHLARIPDYM

WVAEDGMKMQSFGCQLWDTGFAIQALLASNLPDETDDALKRGHNYIKASQVRENPSGDF

RSMYRHISKGAWTFSDRDHGWQVSDCTAEALKCCLLLSMMSADIVGQKIDDEQLYDSVN

LLLSLQSGNGGVNAWEPSRAYKWLELLNPTEFMANTMVEREFVECTSSVIQALDLFRKL

YPDHRKKEINRSIEKAVQFIQDNQTPDGSWYGNWGVCFIYATWFALGGLAAAGETYNDC

LAMRNGVHFLLTTQRDDGGWGESYLSCSEQRYIPSEGERSNLVQTSWAMMALIHTGQAE

RDLIPLHRAAKLIINSQLENGDFPQQEIVGAFMNTCMLHYATYRNTFPLWALAEYRKVV

FIVN"

CDS 5116..5118

/codon_start=1

/label=STOP

/note=second stop codon

/translation=""

CDS 5140..5157

/codon_start=1

/product="6xHis affinity tag"

/label=6xHis

/note=not translated in this construct

/translation="HHHHHH"

terminator 5224..5271

/label=T7 terminator

/note="transcription terminator for bacteriophage T7 RNA

polymerase"

misc_feature 5302..5321

/label=BioBrick Suffix

/note=lacking 5'terminal T

ORIGIN

1 ctgcatgaat tcgcggccgc ttctagagac taaagacaag tgagatagca gtggcaatct

61 ggctttgcaa tcaatgtttc cactaaagcg tttagcgtta ctgcggctag aagtcctcca

121 ccgaggctcc cctgaatggt gatatgggga atgggactgg tcatcagtcg tcgttttgcc

181 cccggagcat gactaaaacc gatcggcatt ccgatcacaa gagccggctg aatatgttgt

241 tgctctatca gcttacaggc agtgagtaaa acagaagggg catagccgat cgccagcaca

301 catccttggg gaatctgttg taaccgctgt tgccaatggt catggtgcca aaaagcttgc

361 tcggcttccc taagccctgt gatgtgaggg tcgtcaatca gcgttttaac cgtacatcct

421 aaatgagcta accgagtttg atcaagagcc gcagccacaa ccggaacatc ggtgacgact

481 ggacaccctg ctttcagtgc atctcgtgcc gaggcgatcg ctccctgact caatcgaacg

541 gcgtttacca agctaacatc accacaggcc agcactaatt gatgtagtaa gtgaatggta

601 atttcagagt aagccgataa atccggtagc aggtgtttga gggattcctg aaaggcttct

661 ggatgagttg ttgtctccgc atctaggttc gtccacaact gatcgagttt tcctaacccc

721 tcctggacat ccacatcaag ctgtttcagt tgggccagag cttccgcttg ggtaatctgg

781 caactctggt cgcgtcccag taatccttct aaagcagatg cggtttggcg gagtcgagta

841 atctgctgaa tcacagcctg atattgctgt tgcaactgca ccattagggt gggatcaagg

901 ctctcttcag aatggctatc cagcagttgc cgaatatgag acaactgaaa gccctgctgt

961 ttgagggcaa tgactcgttg gagccgttgt acgtcctgct gagtataaag gcggtagttg

1021 ccctctgagc gttgaacggg gggaagcaat cccagggtgt ggtaatggcg caccatgcga

1081 ggcgtaacgc cacctcccac tgcatctgtg agttctttaa tcgttaagtg attagtcttc

1141 atccctttag tttactcaaa accttgacat tgacactaat gttaaggttt aggctgagaa

1201 ggtaaaaatc caagttaaaa agcatggcta gccagctgtg gaactggatc ctgccgctgc

1261 tgatcagcag cctgctgatc agcttcgtgg cgttctatgg cttcttcgtg aaaccgaaac

1321 gcaacggcct gcgccatgat cgcaaaaccg tgagcaccgt gaccagcgat gtgggcagcg

1381 tgaacatcac cggcgatacc gtggcggatg tgatcgtggt gggcgcgggc gtggcgggca

1441 gcgcgctggc gtataccctg ggcaaagata aacgccgcgt gcatgtgatc gaacgcgatc

1501 tgagcgaacc ggatcgcatc gtgggcgaac tgctgcagcc gggcggctat ctgaaactgc

1561 tggaactggg catcgaagat tgcgtggaag aaatcgatgc gcagcgcgtg tatggctatg

1621 cgctgttcaa aaacggcaaa cgcatccgcc tggcgtatcc gctggaaaaa ttccatgaag

1681 atgtgagcgg ccgcagcttc cataacggcc gcttcatcca gcgcatgcgc gaaaaagcgg

1741 cgagcctgcc gaacgtgcag ctggaacagg gcaccgtgct gagcctgctg gaagaaaacg

1801 gcaccatcaa aggcgtgcgc tataaaaaca aagcgggcga agaacagacc gcgttcgcgg

1861 cgctgaccat cgtgtgcgat ggctgcttca gcaacctgcg ccgcagcctg tgcaacccgc

1921 aggtggaagt gccgagctgc ttcgtgggcc tggtgctgga aaactgcaac ctgccgtatg

1981 cgaaccatgg ccatgtggtg ctggcggatc cgagcccgat cctgatgtat ccgatcagca

2041 gcaccgaagt gcgctgcctg gtggatgtgc cgggccagaa agtgccgagc atcgcgaacg

2101 gcgaaatgaa aaactatctg aaaaccgtgg tggcgccgca gatgccgcat gaagtgtatg

2161 atagcttcat cgcggcggtg gataaaggca acatcaaaag catgccgaac cgcagcatgc

2221 cggcgagccc gtatccgacc ccgggcgcgc tgctgatggg cgatgcgttc aacatgcgcc

2281 atccgctgac cggcggcggc atgaccgtgg cgctggcgga tatcgtggtg ctgcgcaacc

2341 tgctgcgccc gctgcgcgat ctgagcgatg gcgcgagcct gtgcaaatat ctggagagct

2401 tctataccct gcgcaaaccg gtggcggcga ccatcaacac cctggcgaac gcgctgtatc

2461 aggtgttctg cagcagcgaa aacgaagcgc gcaacgaaat gcgcgaagcg tgcttcgatt

2521 atctgggcct gggcggcatg tgcaccagcg gcccggtgag cctgctgagc ggcctgaacc

2581 cgcgcccgct gaccctggtg tgccatttct tcgcggtggc ggtgtatggc gtgatccgcc

2641 tgctgatccc gttcccgagc ccgaaacgca tctggctggg cgcgaaactg atcagcggcg

2701 cgagcggcat catcttcccg atcatcaaag cggaaggcgt gcgccagatg ttcttcccgg

2761 cgaccgtgcc ggcgtattat tataaagcgc cgaccgtggg cgaaaccaaa tgcagctaaa

2821 cgcgtaaaag aggagaaata ctagatgtgg aaactgaaga tcgggaaggg caacggcgag

2881 gatccccatc tgttttcgtc gaacaacttc gtcggccgcc aaacgtggaa gttcgaccat

2941 aaggccggca gcccggaaga gcgcgccgcc gtggaggagg cccgccgggg cttcctggac

3001 aaccggttcc gcgtcaaggg ctgctccgac ctgctgtggc gcatgcagtt cctgcgcgaa

3061 aaaaaattcg aacagggcat cccccagctg aaagccacga acatcgagga aatcacgtat

3121 gagaccacga cgaacgcgct gcggcgcggg gtgcgctatt tcacggcgct gcaggcgtcc

3181 gacggccact ggcccggcga aatcaccggc ccgctgtttt tcctgccgcc gcttatcttc

3241 tgcctgtata tcacgggcca cctggaagaa gtgttcgacg cggaacaccg caaggagatg

3301 ctgcggcata tctattgcca tcagaatgaa gacggcggct ggggcctgca catcgagtcg

3361 aaaagcgtga tgttttgcac ggtcctgaac tacatctgcc tgcggatgct gggggaaaac

3421 cccgagcagg acgcctgcaa acgcgcccgc caatggatcc tggatcgcgg gggggtgatc

3481 tttatcccgt cgtggggcaa gttctggctg agcatcctgg gcgtctacga ttggtcgggg

3541 accaacccga ccccgcccga gctgctgatg ctgcccagct ttctgccgat ccacccgggc

3601 aagatcctgt gctacagccg catggtgagc atcccgatgt cctatctgta tgggaagcgg

3661 ttcgtcggcc cgatcacgcc gctgatcctt ctgctgcgcg aggaactgta cctggagccc

3721 tatgaagaga tcaattggaa gaagagccgc cgcctgtacg cgaaggagga catgtactat

3781 gcgcacccgc tggtgcaaga tctgctgtcg gacaccctgc agaactttgt cgagccgctg

3841 ctgacgcgct ggcccctgaa taagctggtg cgggagaagg cgctgcagct taccatgaaa

3901 catatccact acgaggacga aaattcgcat tacatcacga tcggctgcgt cgaaaaggtc

3961 ctgtgcatgc tggcctgctg ggtggaaaac ccgaacgggg actattttaa gaagcacctg

4021 gcgcggatcc cggattatat gtgggtggcg gaagacggga tgaaaatgca gagcttcggc

4081 tgccagctgt gggatacggg cttcgcgatc caggccctgc tggcgtcgaa tctgccggac

4141 gagaccgatg acgcccttaa gcggggccac aactacatca aggcctccca ggtccgcgaa

4201 aatccgagcg gcgacttccg cagcatgtac cggcatatct cgaaaggcgc ctggaccttt

4261 tcggaccggg atcatggctg gcaggtgtcc gactgcaccg cggaagccct gaagtgctgc

4321 ctgctgctta gcatgatgtc ggcggacatc gtcgggcaga agatcgatga cgagcaactg

4381 tatgactcgg tgaatctgct gctttcgctt cagtcgggga acggcggcgt caacgcctgg

4441 gagccgtcgc gcgcgtataa atggctggag ctgctgaatc ccaccgagtt catggccaat

4501 acgatggtcg aacgcgaatt cgtggaatgc acgtcgtccg tgatccaggc gctggatctg

4561 tttcggaaac tgtatcccga ccatcggaag aaggagatca atcgctcgat cgagaaagcg

4621 gtccagttca tccaggacaa ccagacgccg gatggctcct ggtacggcaa ctggggggtg

4681 tgcttcatct acgccacctg gttcgccctg gggggcctgg cggcggccgg cgagacctat

4741 aacgattgcc tggcgatgcg gaacggggtg catttcctgc tgaccaccca gcgcgacgat

4801 gggggctggg gcgagtccta cctgagctgc tccgagcagc gctatatccc gtcggaaggc

4861 gaacggtcga acctggtcca gacgtcgtgg gcgatgatgg ccctgatcca taccgggcag

4921 gcggagcgcg acctgatccc gctgcatcgg gcggcgaaac tgatcatcaa cagccagctg

4981 gaaaacggcg actttccgca gcaggaaatc gtgggcgcct tcatgaacac ctgcatgctg

5041 cattacgcga cgtaccgcaa caccttcccc ctgtgggcgc tggcggagta tcgcaaggtc

5101 gtcttcatcg tcaattaaaa gcttgcggcc gcactcgagc accaccacca ccaccactga

5161 gatccggctg ctaacaaagc ccgaaaggaa gctgagttgg ctgctgccac cgctgagcaa

5221 taactagcat aaccccttgg ggcctctaaa cgggtcttga ggggtttttt gctgaaagga

5281 ggaactatat ccggattggc gactagtagc ggccgctgca gtcgac

//

**pVZ-PcoaT-SQE1-THAS1**

LOCUS PcoaT-SQE1-THAS1 5329 bp DNA linear UNA 18-OCT-2017

KEYWORDS pVZ-PcoaT-SQE1-THAS1

FEATURES Location/Qualifiers

source 1..5329

/organism="unspecified"

/mol_type="genomic DNA"

misc_feature 1..6

/label=PstI/NsiI scar

misc_feature 7..28

/label=BioBrick prefix

/note="BioBrick prefix for parts that do not start with

"ATG"

CDS complement(29..1142)

/codon_start=1

/gene="coaR"

/locus_tag="MYO_127660"

/product="MerR family transcriptional regulator"

/label=coaR

/note="cobalt-dependent transcriptional regulator from

Synechocystis sp. PCC 6803; similar to sll0794"

/protein_id="AGF52994"

/translation="MKTNHLTIKELTDAVGGGVTPRMVRHYHTLGLLPPVQRSEGNYRL

YTQQDVQRLQRVIALKQQGFQLSHIRQLLDSHSEESLDPTLMVQLQQQYQAVIQQITRL

RQTASALEGLLGRDQSCQITQAEALAQLKQLDVDVQEGLGKLDQLWTNLDAETTTHPEA

FQESLKHLLPDLSAYSEITIHLLHQLVLACGDVSLVNAVRLSQGAIASARDALKAGCPV

VTDVPVVAAALDQTRLAHLGCTVKTLIDDPHITGLREAEQAFWHHDHWQQRLQQIPQGC

VLAIGYAPSVLLTACKLIEQQHIQPALVIGMPIGFSHAPGAKRRLMTSPIPHITIQGSL

GGGLLAAVTLNALVETLIAKPDCHCYLTCL"

misc_feature 1143..1223

/label=PcoaT+5'UTR

/note="promoter including 5'UTR of coaT gene from

Synechocystis sp. PCC 6803 (Moscow wildtype)"

CDS 1224..2819

/codon_start=1

/gene="SQE1"

/product="A. thaliana squalene epoxidase SQE1"

/label=SQE1

/note="second amino acid changed from changed from Glu to

Ala"

/translation="MASQLWNWILPLLISSLLISFVAFYGFFVKPKRNGLRHDRKTVST

VTSDVGSVNITGDTVADVIVVGAGVAGSALAYTLGKDKRRVHVIERDLSEPDRIVGELL

QPGGYLKLLELGIEDCVEEIDAQRVYGYALFKNGKRIRLAYPLEKFHEDVSGRSFHNGR

FIQRMREKAASLPNVQLEQGTVLSLLEENGTIKGVRYKNKAGEEQTAFAALTIVCDGCF

SNLRRSLCNPQVEVPSCFVGLVLENCNLPYANHGHVVLADPSPILMYPISSTEVRCLVD

VPGQKVPSIANGEMKNYLKTVVAPQMPHEVYDSFIAAVDKGNIKSMPNRSMPASPYPTP

GALLMGDAFNMRHPLTGGGMTVALADIVVLRNLLRPLRDLSDGASLCKYLESFYTLRKP

VAATINTLANALYQVFCSSENEARNEMREACFDYLGLGGMCTSGPVSLLSGLNPRPLTL

VCHFFAVAVYGVIRLLIPFPSPKRIWLGAKLISGASGIIFPIIKAEGVRQMFFPATVPA

YYYKAPTVGETKCS"

RBS 2827..2838

/label=BBa_0034

CDS 2845..5121

/codon_start=1

/gene="THAS1"

/product="A. thaliana thalianol synthase THAS1"

/label=THAS1

/translation="MWRLRTGPKAGEDTHLFTTNNYAGRQIWEFDANAGSPQEIAEVED

ARHKFSDNTSRFKTTADLLWRMQFLREKKFEQKIPRVIIEDARKIKYEDAKTALKRGLL

YFTALQADDGHWPAENSGPNFYTPPFLICLYITGHLEKIFTPEHVKELLRHIYNMQNED

GGWGLHVESHSVMFCTVINYVCLRIVGEEVGHDDQRNGCAKAHKWIMDHGGATYTPLIG

KALLSVLGVYDWSGCNPIPPEFWLLPSSFPVNGGTLWIYLRDTFMGLSYLYGKKFVAPP

TPLILQLREELYPEPYAKINWTQTRNRCGKEDLYYPRSFLQDLFWKSVHMFSESILDRW

PLNKLIRQRALQSTMALIHYHDESTRYITGGCLPKAFHMLACWIEDPKSDYFKKHLARV

REYIWIGEDGLKIQSFGSQLWDTALSLHALLDGIDDHDVDDEIKTTLVKGYDYLKKSQI

TENPRGDHFKMFRHKTKGGWTFSDQDQGWPVSDCTAESLECCLFFESMPSELIGKKMDV

EKLYDAVDYLLYLQSDNGGIAAWQPVEGKAWLELLNIMIFRYVECTGSAIAALTQFNKQ

FPGYKNVEVKRFITKAAKYIEDMQTVDGSWYGNWGVCFIYGTFFAVRGLVAAGKTYSNC

EAIRKAVRFLLDTQNPEGGWGESFLSCPSKKYTPLKGNSTNVVQTAQALMVLIMGDQME

RDPLPVHRAAQVLINSQLDNGDFPQQEIMGTFMRTVMLHFPTYRNTFSLWALTHYTHAL

RRLLP"

CDS 5143..5160

/codon_start=1

/product="6xHis affinity tag"

/label=6xHis

/note=not translated in this construct

/translation="HHHHHH"

terminator 5227..5274

/label=T7 terminator

/note="transcription terminator for bacteriophage T7 RNA

polymerase"

misc_feature 5305..5324

/label=BioBrick Suffix

/note=lacking 5'terminal T

ORIGIN

1 ctgcatgaat tcgcggccgc ttctagagac taaagacaag tgagatagca gtggcaatct

61 ggctttgcaa tcaatgtttc cactaaagcg tttagcgtta ctgcggctag aagtcctcca

121 ccgaggctcc cctgaatggt gatatgggga atgggactgg tcatcagtcg tcgttttgcc

181 cccggagcat gactaaaacc gatcggcatt ccgatcacaa gagccggctg aatatgttgt

241 tgctctatca gcttacaggc agtgagtaaa acagaagggg catagccgat cgccagcaca

301 catccttggg gaatctgttg taaccgctgt tgccaatggt catggtgcca aaaagcttgc

361 tcggcttccc taagccctgt gatgtgaggg tcgtcaatca gcgttttaac cgtacatcct

421 aaatgagcta accgagtttg atcaagagcc gcagccacaa ccggaacatc ggtgacgact

481 ggacaccctg ctttcagtgc atctcgtgcc gaggcgatcg ctccctgact caatcgaacg

541 gcgtttacca agctaacatc accacaggcc agcactaatt gatgtagtaa gtgaatggta

601 atttcagagt aagccgataa atccggtagc aggtgtttga gggattcctg aaaggcttct

661 ggatgagttg ttgtctccgc atctaggttc gtccacaact gatcgagttt tcctaacccc

721 tcctggacat ccacatcaag ctgtttcagt tgggccagag cttccgcttg ggtaatctgg

781 caactctggt cgcgtcccag taatccttct aaagcagatg cggtttggcg gagtcgagta

841 atctgctgaa tcacagcctg atattgctgt tgcaactgca ccattagggt gggatcaagg

901 ctctcttcag aatggctatc cagcagttgc cgaatatgag acaactgaaa gccctgctgt

961 ttgagggcaa tgactcgttg gagccgttgt acgtcctgct gagtataaag gcggtagttg

1021 ccctctgagc gttgaacggg gggaagcaat cccagggtgt ggtaatggcg caccatgcga

1081 ggcgtaacgc cacctcccac tgcatctgtg agttctttaa tcgttaagtg attagtcttc

1141 atccctttag tttactcaaa accttgacat tgacactaat gttaaggttt aggctgagaa

1201 ggtaaaaatc caagttaaaa agcatggcta gccagctgtg gaactggatc ctgccgctgc

1261 tgatcagcag cctgctgatc agcttcgtgg cgttctatgg cttcttcgtg aaaccgaaac

1321 gcaacggcct gcgccatgat cgcaaaaccg tgagcaccgt gaccagcgat gtgggcagcg

1381 tgaacatcac cggcgatacc gtggcggatg tgatcgtggt gggcgcgggc gtggcgggca

1441 gcgcgctggc gtataccctg ggcaaagata aacgccgcgt gcatgtgatc gaacgcgatc

1501 tgagcgaacc ggatcgcatc gtgggcgaac tgctgcagcc gggcggctat ctgaaactgc

1561 tggaactggg catcgaagat tgcgtggaag aaatcgatgc gcagcgcgtg tatggctatg

1621 cgctgttcaa aaacggcaaa cgcatccgcc tggcgtatcc gctggaaaaa ttccatgaag

1681 atgtgagcgg ccgcagcttc cataacggcc gcttcatcca gcgcatgcgc gaaaaagcgg

1741 cgagcctgcc gaacgtgcag ctggaacagg gcaccgtgct gagcctgctg gaagaaaacg

1801 gcaccatcaa aggcgtgcgc tataaaaaca aagcgggcga agaacagacc gcgttcgcgg

1861 cgctgaccat cgtgtgcgat ggctgcttca gcaacctgcg ccgcagcctg tgcaacccgc

1921 aggtggaagt gccgagctgc ttcgtgggcc tggtgctgga aaactgcaac ctgccgtatg

1981 cgaaccatgg ccatgtggtg ctggcggatc cgagcccgat cctgatgtat ccgatcagca

2041 gcaccgaagt gcgctgcctg gtggatgtgc cgggccagaa agtgccgagc atcgcgaacg

2101 gcgaaatgaa aaactatctg aaaaccgtgg tggcgccgca gatgccgcat gaagtgtatg

2161 atagcttcat cgcggcggtg gataaaggca acatcaaaag catgccgaac cgcagcatgc

2221 cggcgagccc gtatccgacc ccgggcgcgc tgctgatggg cgatgcgttc aacatgcgcc

2281 atccgctgac cggcggcggc atgaccgtgg cgctggcgga tatcgtggtg ctgcgcaacc

2341 tgctgcgccc gctgcgcgat ctgagcgatg gcgcgagcct gtgcaaatat ctggagagct

2401 tctataccct gcgcaaaccg gtggcggcga ccatcaacac cctggcgaac gcgctgtatc

2461 aggtgttctg cagcagcgaa aacgaagcgc gcaacgaaat gcgcgaagcg tgcttcgatt

2521 atctgggcct gggcggcatg tgcaccagcg gcccggtgag cctgctgagc ggcctgaacc

2581 cgcgcccgct gaccctggtg tgccatttct tcgcggtggc ggtgtatggc gtgatccgcc

2641 tgctgatccc gttcccgagc ccgaaacgca tctggctggg cgcgaaactg atcagcggcg

2701 cgagcggcat catcttcccg atcatcaaag cggaaggcgt gcgccagatg ttcttcccgg

2761 cgaccgtgcc ggcgtattat tataaagcgc cgaccgtggg cgaaaccaaa tgcagctaaa

2821 cgcgtaaaag aggagaaata ctagatgtgg cgcctgcgca ccggcccgaa agcgggcgaa

2881 gatacccatc tgttcaccac caacaactat gcgggccgcc agatctggga attcgatgcg

2941 aacgcgggca gcccgcagga aatcgcggaa gtggaagatg cgcgccataa attcagcgat

3001 aacaccagcc gcttcaaaac caccgcggat ctgctgtggc gcatgcagtt cctgcgcgaa

3061 aaaaaattcg aacagaaaat cccgcgcgtg atcatcgaag atgcgcgcaa aatcaaatat

3121 gaagatgcga aaaccgcgct gaaacgcggc ctgctgtatt tcaccgcgct gcaggcggat

3181 gatggccatt ggccggcgga aaacagcggc ccgaacttct ataccccgcc gttcctgatc

3241 tgcctgtata tcaccggcca tctggaaaaa atcttcaccc cggaacatgt gaaagaactg

3301 ctgcgccata tctataacat gcagaacgaa gatggcggct ggggcctgca tgtggaaagc

3361 catagcgtga tgttctgcac cgtgatcaac tatgtgtgcc tgcgcatcgt gggcgaagaa

3421 gtgggccatg atgatcagcg caacggctgc gcgaaagcgc ataaatggat catggatcat

3481 ggcggcgcga cctatacccc gctgatcggc aaagcgctgc tgagcgtgct gggcgtgtat

3541 gattggagcg gctgcaaccc gatcccgccg gaattctggc tgctgccgag cagcttcccg

3601 gtgaacggcg gcaccctgtg gatctatctg cgcgatacct tcatgggcct gagctatctg

3661 tatggcaaaa aattcgtggc gccgccgacc ccgctgatcc tgcagctgcg cgaagaactg

3721 tatccggaac cgtatgcgaa aatcaactgg acccagaccc gcaaccgctg cggcaaagaa

3781 gatctgtatt atccgcgcag cttcctgcag gatctgttct ggaaaagcgt gcacatgttc

3841 agcgaaagca tcctggatcg ctggccgctg aacaaactga tccgccagcg cgcgctgcag

3901 agcaccatgg cgctgatcca ttatcatgat gaaagcaccc gctatatcac cggcggctgc

3961 ctgccgaaag cgttccacat gctggcgtgc tggatcgaag atccgaaaag cgattatttc

4021 aaaaaacatc tggcgcgcgt gcgcgaatat atctggatcg gcgaagatgg cctgaaaatc

4081 cagagcttcg gcagccagct gtgggatacc gcgctgagcc tgcatgcgct gctggatggc

4141 atcgatgatc atgatgtgga tgatgaaatc aaaaccaccc tggtgaaagg ctatgattat

4201 ctgaaaaaaa gccagatcac cgaaaacccg cgcggcgatc atttcaaaat gttccgccat

4261 aaaaccaaag gcggctggac cttcagcgat caggatcagg gctggccggt gagcgattgc

4321 accgcggaaa gcctggaatg ctgcctgttc ttcgaaagca tgccgagcga actgatcggc

4381 aaaaaaatgg atgtggaaaa actgtatgat gcggtggatt atctgctgta tctgcagagc

4441 gataacggcg gcatcgcggc gtggcagccg gtggaaggca aagcgtggct ggaactgctg

4501 aacatcatga tcttccgcta tgtggaatgc accggcagcg cgatcgcggc gctgacccag

4561 ttcaacaaac agttcccggg ctataaaaac gtggaagtga aacgcttcat caccaaagcg

4621 gcgaaatata tcgaagatat gcagaccgtg gatggcagct ggtatggcaa ctggggcgtg

4681 tgcttcatct atggcacctt ctttgcggtg cgcggcctgg tggcggcggg caaaacctat

4741 agcaactgcg aagcgatccg caaagcggtg cgcttcctgc tggataccca gaacccggaa

4801 ggcggctggg gcgagagctt cctgagctgc ccgagcaaaa aatatacccc gctgaaaggc

4861 aacagcacca acgtggtgca gaccgcgcag gcgctgatgg tgctgatcat gggcgatcag

4921 atggaacgcg atccgctgcc ggtgcatcgc gcggcgcagg tgctgatcaa cagccagctg

4981 gataacggcg atttcccgca gcaggaaatc atgggcacct tcatgcgcac cgtgatgctg

5041 catttcccga cctatcgcaa caccttcagc ctgtgggcgc tgacccatta tacccatgcg

5101 ctgcgccgcc tgctgccgta aaagcttgcg gccgcactcg agcaccacca ccaccaccac

5161 tgagatccgg ctgctaacaa agcccgaaag gaagctgagt tggctgctgc caccgctgag

5221 caataactag cataacccct tggggcctct aaacgggtct tgaggggttt tttgctgaaa

5281 ggaggaacta tatccggatt ggcgactagt agcggccgct gcagtcgac

//

**pVZ-PcoaT-SQE1-MRN1**

LOCUS PcoaT-SQE1-MRN1 5338 bp DNA linear UNA 18-OCT-2017

KEYWORDS pVZ-PcoaT-SQE1-MRN1

FEATURES Location/Qualifiers

source 1..5338

/organism="unspecified"

/mol_type="genomic DNA"

misc_feature 1..6

/label=PstI/NsiI scar

misc_feature 7..28

/label=BioBrick prefix

/note="BioBrick prefix for parts that do not start with

"ATG"

CDS complement(29..1142)

/codon_start=1

/gene="coaR"

/locus_tag="MYO_127660"

/product="MerR family transcriptional regulator"

/label=coaR

/note="cobalt-dependent transcriptional regulator from

Synechocystis sp. PCC 6803; similar to sll0794"

/protein_id="AGF52994"

/translation="MKTNHLTIKELTDAVGGGVTPRMVRHYHTLGLLPPVQRSEGNYRL

YTQQDVQRLQRVIALKQQGFQLSHIRQLLDSHSEESLDPTLMVQLQQQYQAVIQQITRL

RQTASALEGLLGRDQSCQITQAEALAQLKQLDVDVQEGLGKLDQLWTNLDAETTTHPEA

FQESLKHLLPDLSAYSEITIHLLHQLVLACGDVSLVNAVRLSQGAIASARDALKAGCPV

VTDVPVVAAALDQTRLAHLGCTVKTLIDDPHITGLREAEQAFWHHDHWQQRLQQIPQGC

VLAIGYAPSVLLTACKLIEQQHIQPALVIGMPIGFSHAPGAKRRLMTSPIPHITIQGSL

GGGLLAAVTLNALVETLIAKPDCHCYLTCL"

misc_feature 1143..1223

/label=PcoaT+5'UTR

/note="promoter including 5'UTR of coaT gene from

Synechocystis sp. PCC 6803 (Moscow wildtype)"

CDS 1224..2819

/codon_start=1

/gene="SQE1"

/product="A. thaliana squalene epoxidase SQE1"

/label=SQE1

/note="second amino acid changed from changed from Glu to

Ala"

/translation="MASQLWNWILPLLISSLLISFVAFYGFFVKPKRNGLRHDRKTVST

VTSDVGSVNITGDTVADVIVVGAGVAGSALAYTLGKDKRRVHVIERDLSEPDRIVGELL

QPGGYLKLLELGIEDCVEEIDAQRVYGYALFKNGKRIRLAYPLEKFHEDVSGRSFHNGR

FIQRMREKAASLPNVQLEQGTVLSLLEENGTIKGVRYKNKAGEEQTAFAALTIVCDGCF

SNLRRSLCNPQVEVPSCFVGLVLENCNLPYANHGHVVLADPSPILMYPISSTEVRCLVD

VPGQKVPSIANGEMKNYLKTVVAPQMPHEVYDSFIAAVDKGNIKSMPNRSMPASPYPTP

GALLMGDAFNMRHPLTGGGMTVALADIVVLRNLLRPLRDLSDGASLCKYLESFYTLRKP

VAATINTLANALYQVFCSSENEARNEMREACFDYLGLGGMCTSGPVSLLSGLNPRPLTL

VCHFFAVAVYGVIRLLIPFPSPKRIWLGAKLISGASGIIFPIIKAEGVRQMFFPATVPA

YYYKAPTVGETKCS"

RBS 2827..2838

/label=BBa_0034

CDS 2845..5130

/codon_start=1

/gene="MRN1"

/product="A. thaliana marneral synthase MRN1"

/label=MRN1

/translation="MWRLRIGAEARQDPHLFTTNNFAGRQIWEFDANGGSPEELAEVEE

ARLNFANNKSRFKASPDLFWRRQFLREKKFEQKIPRVRIEDAEKITYEDAKTALRRGVL

YYAACQANDGHWPSEVSGSMFLDAPFVICLYITGHLEKIFTLEHVKELLRYMYNTQNED

GGWGLDVESHSVMFCTVLNYICLRILGVEPDHDGQKSACARARKWILDHGGATYAPMVA

KAWLSVLGVYDWSGCKPLPPEIWMLPSFSPINGGTLWIYIRDLLMGMSYLYGKKFVATP

TALILQLREELYPQPYSKIIWSKARNRCAKEDLLYPKSFGQDLFWEGVHMLSENIINRW

PLNKFVRQRALRTTMELVHYHDETTHYITGACVAKPFHMLACWVEDPDGDYFKKHLARV

PDFIWIAEDGLKFQLMGMQSWNAALSLQVMLAANMDDEIRSTLIKGYDFLKQSQISENP

QGDHLKMFRDITKGGWTFQDREQGLPISDGTAESIECCIHFHRMPSEFIGEKMDVEKLY

DAVNFLIYLQSDNGGMPVWEPAPGKKWLEWLSPVEHVENTVVEQEYLECTGSVIAGLVC

FKKEFPDHRPKEIEKLIKKGLKYIEDLQMPDGSWYGNWGVCFTYGTLFAVRGLAAAGKT

FGNSEAIRRAVQFILNTQNAEGGWGESALSCPNKKYIPSKGNVTNVVNTGQAMMVLLIG

GQMERDPSPVHRAAKVLINSQLDIGDFPQQERRGIYMNMLLHYPTYRNMFSLWALALYT

NALRLLVS"

CDS 5152..5169

/codon_start=1

/product="6xHis affinity tag"

/label=6xHis

/note=not translated in this construct

/translation="HHHHHH"

terminator 5236..5283

/label=T7 terminator

/note="transcription terminator for bacteriophage T7 RNA

polymerase"

misc_feature 5314..5333

/label=BioBrick Suffix

/note=lacking 5'terminal T

ORIGIN

1 ctgcatgaat tcgcggccgc ttctagagac taaagacaag tgagatagca gtggcaatct

61 ggctttgcaa tcaatgtttc cactaaagcg tttagcgtta ctgcggctag aagtcctcca

121 ccgaggctcc cctgaatggt gatatgggga atgggactgg tcatcagtcg tcgttttgcc

181 cccggagcat gactaaaacc gatcggcatt ccgatcacaa gagccggctg aatatgttgt

241 tgctctatca gcttacaggc agtgagtaaa acagaagggg catagccgat cgccagcaca

301 catccttggg gaatctgttg taaccgctgt tgccaatggt catggtgcca aaaagcttgc

361 tcggcttccc taagccctgt gatgtgaggg tcgtcaatca gcgttttaac cgtacatcct

421 aaatgagcta accgagtttg atcaagagcc gcagccacaa ccggaacatc ggtgacgact

481 ggacaccctg ctttcagtgc atctcgtgcc gaggcgatcg ctccctgact caatcgaacg

541 gcgtttacca agctaacatc accacaggcc agcactaatt gatgtagtaa gtgaatggta

601 atttcagagt aagccgataa atccggtagc aggtgtttga gggattcctg aaaggcttct

661 ggatgagttg ttgtctccgc atctaggttc gtccacaact gatcgagttt tcctaacccc

721 tcctggacat ccacatcaag ctgtttcagt tgggccagag cttccgcttg ggtaatctgg

781 caactctggt cgcgtcccag taatccttct aaagcagatg cggtttggcg gagtcgagta

841 atctgctgaa tcacagcctg atattgctgt tgcaactgca ccattagggt gggatcaagg

901 ctctcttcag aatggctatc cagcagttgc cgaatatgag acaactgaaa gccctgctgt

961 ttgagggcaa tgactcgttg gagccgttgt acgtcctgct gagtataaag gcggtagttg

1021 ccctctgagc gttgaacggg gggaagcaat cccagggtgt ggtaatggcg caccatgcga

1081 ggcgtaacgc cacctcccac tgcatctgtg agttctttaa tcgttaagtg attagtcttc

1141 atccctttag tttactcaaa accttgacat tgacactaat gttaaggttt aggctgagaa

1201 ggtaaaaatc caagttaaaa agcatggcta gccagctgtg gaactggatc ctgccgctgc

1261 tgatcagcag cctgctgatc agcttcgtgg cgttctatgg cttcttcgtg aaaccgaaac

1321 gcaacggcct gcgccatgat cgcaaaaccg tgagcaccgt gaccagcgat gtgggcagcg

1381 tgaacatcac cggcgatacc gtggcggatg tgatcgtggt gggcgcgggc gtggcgggca

1441 gcgcgctggc gtataccctg ggcaaagata aacgccgcgt gcatgtgatc gaacgcgatc

1501 tgagcgaacc ggatcgcatc gtgggcgaac tgctgcagcc gggcggctat ctgaaactgc

1561 tggaactggg catcgaagat tgcgtggaag aaatcgatgc gcagcgcgtg tatggctatg

1621 cgctgttcaa aaacggcaaa cgcatccgcc tggcgtatcc gctggaaaaa ttccatgaag

1681 atgtgagcgg ccgcagcttc cataacggcc gcttcatcca gcgcatgcgc gaaaaagcgg

1741 cgagcctgcc gaacgtgcag ctggaacagg gcaccgtgct gagcctgctg gaagaaaacg

1801 gcaccatcaa aggcgtgcgc tataaaaaca aagcgggcga agaacagacc gcgttcgcgg

1861 cgctgaccat cgtgtgcgat ggctgcttca gcaacctgcg ccgcagcctg tgcaacccgc

1921 aggtggaagt gccgagctgc ttcgtgggcc tggtgctgga aaactgcaac ctgccgtatg

1981 cgaaccatgg ccatgtggtg ctggcggatc cgagcccgat cctgatgtat ccgatcagca

2041 gcaccgaagt gcgctgcctg gtggatgtgc cgggccagaa agtgccgagc atcgcgaacg

2101 gcgaaatgaa aaactatctg aaaaccgtgg tggcgccgca gatgccgcat gaagtgtatg

2161 atagcttcat cgcggcggtg gataaaggca acatcaaaag catgccgaac cgcagcatgc

2221 cggcgagccc gtatccgacc ccgggcgcgc tgctgatggg cgatgcgttc aacatgcgcc

2281 atccgctgac cggcggcggc atgaccgtgg cgctggcgga tatcgtggtg ctgcgcaacc

2341 tgctgcgccc gctgcgcgat ctgagcgatg gcgcgagcct gtgcaaatat ctggagagct

2401 tctataccct gcgcaaaccg gtggcggcga ccatcaacac cctggcgaac gcgctgtatc

2461 aggtgttctg cagcagcgaa aacgaagcgc gcaacgaaat gcgcgaagcg tgcttcgatt

2521 atctgggcct gggcggcatg tgcaccagcg gcccggtgag cctgctgagc ggcctgaacc

2581 cgcgcccgct gaccctggtg tgccatttct tcgcggtggc ggtgtatggc gtgatccgcc

2641 tgctgatccc gttcccgagc ccgaaacgca tctggctggg cgcgaaactg atcagcggcg

2701 cgagcggcat catcttcccg atcatcaaag cggaaggcgt gcgccagatg ttcttcccgg

2761 cgaccgtgcc ggcgtattat tataaagcgc cgaccgtggg cgaaaccaaa tgcagctaaa

2821 cgcgtaaaag aggagaaata ctagatgtgg cgcctgcgca tcggcgcgga agcgcgccag

2881 gatccgcatc tgttcaccac caacaacttc gcgggccgcc agatctggga attcgatgcg

2941 aacggcggca gcccggaaga actggcggaa gtggaagaag cgcgcctgaa cttcgcgaac

3001 aacaaaagcc gcttcaaagc gagcccggat ctgttctggc gccgccagtt cctgcgcgaa

3061 aaaaaattcg aacagaaaat cccgcgcgtg cgcatcgaag atgcggaaaa aatcacctat

3121 gaagatgcga aaaccgcgct gcgccgcggc gtgctgtatt atgcggcgtg ccaggcgaac

3181 gatggccatt ggccgagcga agtgagcggc agcatgttcc tggatgcgcc gttcgtgatc

3241 tgcctgtata tcaccggcca tctggaaaaa atcttcaccc tggaacatgt gaaagaactg

3301 ctgcgctata tgtataacac ccagaacgaa gatggcggct ggggcctgga tgtggaaagc

3361 catagcgtga tgttctgcac cgtgctgaac tatatctgcc tgcgcatcct gggcgtggaa

3421 ccggatcatg atggccagaa aagcgcgtgc gcgcgcgcgc gcaaatggat cctggatcat

3481 ggcggcgcga cctatgcgcc gatggtggcg aaagcgtggc tgagcgtgct gggcgtgtat

3541 gattggagcg gctgcaaacc gctgccgccg gaaatctgga tgctgccgag cttcagcccg

3601 atcaacggcg gcaccctgtg gatctatatc cgcgatctgc tgatgggcat gagctatctg

3661 tatggcaaga aattcgtggc gaccccgacc gcgctgatcc tgcagctgcg cgaagaactg

3721 tatccgcagc cgtatagcaa aatcatctgg agcaaagcgc gcaaccgctg cgcgaaagaa

3781 gatctgctgt atccgaagag cttcggccag gatctgttct gggaaggcgt gcacatgctg

3841 agcgaaaaca tcatcaaccg ctggccgctg aacaaattcg tgcgccagcg cgcgctgcgc

3901 accaccatgg aactggtgca ttatcatgat gaaaccaccc attatatcac cggcgcgtgc

3961 gtggcgaaac cgttccacat gctggcgtgc tgggtggaag atccggatgg cgattatttc

4021 aagaaacatc tggcgcgcgt gccggatttc atctggatcg cggaagatgg cctgaaattc

4081 cagctgatgg gcatgcagag ctggaacgcg gcgctgagcc tgcaggtgat gctggcggcg

4141 aacatggatg atgaaatccg cagcaccctg atcaaaggct atgatttcct gaaacagagc

4201 cagatcagcg aaaacccgca gggcgatcat ctgaaaatgt tccgcgatat caccaaaggc

4261 ggctggacct tccaggatcg cgaacagggc ctgccgatca gcgatggcac cgcggaaagc

4321 atcgaatgct gcatccattt ccatcgcatg ccgagcgaat tcatcggcga aaaaatggat

4381 gtggaaaaac tgtatgatgc ggtgaacttc ctgatctatc tgcagagcga taacggcggc

4441 atgccggtgt gggaaccggc gccgggcaag aaatggctgg aatggctgag cccggtggaa

4501 catgtggaaa acaccgtggt ggaacaggaa tatctggaat gcaccggcag cgtgatcgcg

4561 ggcctggtgt gcttcaagaa agaattcccg gatcatcgcc cgaaagaaat cgaaaaactg

4621 atcaagaaag gcctgaaata tatcgaagat ctgcagatgc cggatggcag ctggtatggc

4681 aactggggcg tgtgcttcac ctatggcacc ctgtttgcgg tgcgcggcct ggcggcggcg

4741 ggcaaaacct tcggcaacag cgaagcgatc cgccgcgcgg tgcagttcat cctgaacacc

4801 cagaacgcgg aaggcggctg gggcgaaagc gcgctgagct gcccgaacaa gaaatatatc

4861 ccgagcaaag gcaacgtgac caacgtggtg aacaccggcc aggcgatgat ggtgctgctg

4921 atcggcggcc agatggaacg cgatccgagc ccggtgcatc gcgcggcgaa agtgctgatc

4981 aacagccagc tggatatcgg cgatttcccg cagcaggaac gccgcggcat ctatatgaac

5041 atgctgctgc attatccgac ctatcgcaac atgttcagcc tgtgggcgct ggcgctgtat

5101 accaacgcgc tgcgcctgct ggtgagctaa aagcttgcgg ccgcactcga gcaccaccac

5161 caccaccact gagatccggc tgctaacaaa gcccgaaagg aagctgagtt ggctgctgcc

5221 accgctgagc aataactagc ataacccctt ggggcctcta aacgggtctt gaggggtttt

5281 ttgctgaaag gaggaactat atccggattg gcgactagta gcggccgctg cagtcgac

//

**pVZ-spec**

LOCUS pVZ-spec 10502 bp DNA circular SYN 25-OCT-2017

KEYWORDS pVZ-spec vector backbone

REFERENCE 1

AUTHORS Zinchenko VV, Piven IV, Melnik VA, Shestakov SV.

TITLE Vectors for the Complementation Cyanobacterial Mutants

JOURNAL Russian Journal of Genetics, Vol. 35. No. 3. 1999, pp. 228-232

REFERENCE 2

AUTHORS Mitschke J, Georg J, Scholz I, Sharma CM, Dienst D, Bantscheff J,

Voß B, Steglich C, Wilde A, Vogel J, Hess WR

TITLE An experimentally anchored map of transcriptional start sites in the

model cyanobacterium Synechocystis sp. PCC6803

JOURNAL Proc Natl Acad Sci USA. 2011;108:2124-2129

REFERENCE 3

AUTHORS Karradt A, Lockau W

TITLE Unpublished

FEATURES Location/Qualifiers

source 1..10502

/organism="synthetic DNA construct"

/mol_type="other DNA"

CDS 959..1618

/codon_start=1

/gene="cat"

/product="chloramphenicol acetyltransferase"

/label=CmR

/note="confers resistance to chloramphenicol"

/translation="MEKKITGYTTVDISQWHRKEHFEAFQSVAQCTYNQTVQLDITAFL

KTVKKNKHKFYPAFIHILARLMNAHPEFRMAMKDGELVIWDSVHPCYTVFHEQTETFSS

LWSEYHDDFRQFLHIYSQDVACYGENLAYFPKGFIENMFFVSANPWVSFTSFDLNVANM

DNFFAPVFTMGKYYTQGDKVLMPLAIQVHHAVCDGFHVGRMLNELQQYCDEWQGGA"

CDS complement(1770..2561)

/codon_start=1

/gene="aadA"

/product="aminoglycoside adenylyltransferase (Murphy,

1985)"

/label=SmR

/note="confers resistance to spectinomycin and

streptomycin"

/translation="MREAVIAEVSTQLSEVVGVIERHLEPTLLAVHLYGSAVDGGLKPH

SDIDLLVTVTVRLDETTRRALINDLLETSASPGESEILRAVEVTIVVHDDIIPWRYPAK

RELQFGEWQRNDILAGIFEPATIDIDLAILLTKAREHSVALVGPAAEELFDPVPEQDLF

EALNETLTLWNSPPDWAGDERNVVLTLSRIWYSAVTGKIAPKDVAADWAMERLPAQYQP

VILEARQAYLGQEEDRLASRADQLEEFVHYVKGEITKVVGK"

CDS complement(3409..4224)

/codon_start=1

/product="aminoglycoside phosphotransferase"

/label=KanR

/note="confers resistance to kanamycin in bacteria or G418

(Geneticin(R)) in eukaryotes"

/translation="MSHIQRETSCSRPRLNSNMDADLYGYKWARDNVGQSGATIYRLYG

KPDAPELFLKHGKGSVANDVTDEMVRLNWLTEFMPLPTIKHFIRTPDDAWLLTTAIPGK

TAFQVLEEYPDSGENIVDALAVFLRRLHSIPVCNCPFNSDRVFRLAQAQSRMNNGLVDA

SDFDDERNGWPVEQVWKEMHKLLPFSPDSVVTHGDFSLDNLIFDEGKLIGCIDVGRVGI

ADRYQDLAILWNCLGEFSPSLQKRLFQKYGIDNPDMNKLQFHLMLDEFF"

rep_origin 5082..5476

/label=RSF1010 oriV

/note="replication origin of the broad-host-range plasmid

RSF1010 from Salmonella enterica serovar Typhimurium;

requires the RSF1010 RepA/B/C proteins for replication"

misc_feature 5498..5787

/label=mobC

misc_feature 5986..8115

/label=mobA

misc_feature 6734..7147

/label=mobB

CDS 7144..8115

/codon_start=1

/gene="repB"

/product="replication protein B of the broad-host-range

plasmid RSF1010 from Salmonella enterica serovar

Typhimurium"

/label=RSF1010 RepB

/translation="MKNDRTLQAIGRQLKAMGCERFDIGVRDATTGQMMNREWSAAEVL

QNTPWLKRMNAQGNDVYIRPAEQERHGLVLVDDLSEFDLDDMKAEGREPALVVETSPKN

YQAWVKVADAAGGELRGQIARTLASEYDADPASADSRHYGRLAGFTNRKDKHTTRAGYQ

PWVLLRESKGKTATAGPALVQQAGQQIEQAQRQQEKARRLASLELPERQLSRHRRTALD

EYRSEMAGLVKRFGDDLSKCDFIAAQKLASRGRSAEEIGKAMAEASPALAERKPGHEAD

YIERTVSKVMGLPSVQLARAELARAPAPRQRGMDRGGPDFSM"

CDS 8626..9465

/codon_start=1

/gene="repA"

/product="replication protein A of the broad-host-range

plasmid RSF1010 from Salmonella enterica serovar

Typhimurium"

/label=RSF1010 RepA

/translation="MATHKPINILEAFAAAPPPLDYVLPNMVAGTVGALVSPGGAGKSM

LALQLAAQIAGGPDLLEVGELPTGPVIYLPAEDPPTAIHHRLHALGAHLSAEERQAVAD

GLLIQPLIGSLPNIMAPEWFDGLKRAAEGRRLMVLDTLRRFHIEEENASGPMAQVIGRM

EAIAADTGCSIVFLHHASKGAAMMGAGDQQQASRGSSVLVDNIRWQSYLSSMTSAEAEE

WGVDDDQRRFFVRFGVSKANYGAPFADRWFRRHDGGVLKPAVLERQRKSKGVPRGEA"

CDS 9452..10303

/codon_start=1

/gene="repC"

/product="replication protein C of the broad-host-range

plasmid RSF1010 from Salmonella enterica serovar

Typhimurium"

/label=RSF1010 RepC

/translation="MVKPKNKHSLSHVRHDPAHCLAPGLFRALKRGERKRSKLDVTYDY

GDGKRIEFSGPEPLGADDLRILQGLVAMAGPNGLVLGPEPKTEGGRQLRLFLEPKWEAV

TAECHVVKGSYRALAKEIGAEVDSGGALKHIQDCIERLWKVSIIAQNGRKRQGFRLLSE

YASDEADGRLYVALNPLIAQAVMGGGQHVRISMDEVRALDSETARLLHQRLCGWIDPGK

TGKASIDTLCGYVWPSEASGSTMRKRRQRVREALPELVALGWTVTEFAAGKYDITRPKA

AG"

ORIGIN

1 cctgcaggca tcgtggtgtc acgctcgtcg tttggtatgg cttcattcag ctccggttcc

61 caacgatcaa ggcgagttac atgatccccc atgttgtgca aaaaagcggt tagctccttc

121 ggtcctccga tcgttgtcag aagtaagttg gccgcagtgt tatcactcat ggttatggca

181 gcactgcata attctcttac tgtcatgcca tccgtaagat gcttttctgt gactggtgag

241 tactcaacca agtcattctg agaatagtgt atgcggcgac cgagttgctc ttgcccggcg

301 tcaacacggg ataataccgc gccacatagc agaactttaa aagtgctcat cattggaaaa

361 cgttcttcgg ggcgaaaact ctcaaggatc ttaccgctgt tgagatccag ttcgatgtaa

421 cccactcgtg cacccaactg atcttcagca tcttttactt tcaccagcgt ttctgggtga

481 gcaaaaacag gaaggcaaaa tgccgcaaaa aagggaataa gggcgacacg gaaatgttga

541 atactcatac tcttcctttt tcaatattat tgaagcattt atcagggtta ttgtctcatg

601 agcggataca tatttgaatg tatttagaaa aataaacaaa taggggttcc gcgcacattt

661 ccccgaaaag tgccacctga cgtctaagaa accattatta tcatgacatt aacctataaa

721 aataggcgta tcacgaggcc ctttcgtctt cgaataaata cctgtgacgg aagatcactt

781 cgcagaataa ataaatcctg gtgtccctgt tgataccggg aagccctggg ccaacttttg

841 gcgaaaatga gacgttgatc ggcacgtaag aggttccaac tttcaccata atgaaataag

901 atcactaccg ggcgtatttt ttgagttatc gagattttca ggagctaagg aagctaaaat

961 ggagaaaaaa atcactggat ataccaccgt tgatatatcc caatggcatc gtaaagaaca

1021 ttttgaggca tttcagtcag ttgctcaatg tacctataac cagaccgttc agctggatat

1081 tacggccttt ttaaagaccg taaagaaaaa taagcacaag ttttatccgg cctttattca

1141 cattcttgcc cgcctgatga atgctcatcc ggaattccgt atggcaatga aagacggtga

1201 gctggtgata tgggatagtg ttcacccttg ttacaccgtt ttccatgagc aaactgaaac

1261 gttttcatcg ctctggagtg aataccacga cgatttccgg cagtttctac acatatattc

1321 gcaagatgtg gcgtgttacg gtgaaaacct ggcctatttc cctaaagggt ttattgagaa

1381 tatgtttttc gtctcagcca atccctgggt gagtttcacc agttttgatt taaacgtggc

1441 caatatggac aacttcttcg cccccgtttt caccatgggc aaatattata cgcaaggcga

1501 caaggtgctg atgccgctgg cgattcaggt tcatcatgcc gtctgtgatg gcttccatgt

1561 cggcagaatg cttaatgaat tacaacagta ctgcgatgag tggcagggcg gggcgtaatt

1621 tttttaaggc agttattggt gcccttaaac gcctggtgct acgcctgaat aagtgataat

1681 aagcggatga atggcagaaa ttcgtcgact ctagagcttg agttaagccg cgccgcgaag

1741 cggcgtcggc ttgaacgaat tgttagacat tatttgccga ctaccttggt gatctcgcct

1801 ttcacgtagt ggacaaattc ttccaactga tctgcgcgcg aggccaagcg atcttcttct

1861 tgtccaagat aagcctgtct agcttcaagt atgacgggct gatactgggc cggcaggcgc

1921 tccattgccc agtcggcagc gacatccttc ggcgcgattt tgccggttac tgcgctgtac

1981 caaatgcggg acaacgtaag cactacattt cgctcatcgc cagcccagtc gggcggcgag

2041 ttccatagcg ttaaggtttc atttagcgcc tcaaatagat cctgttcagg aaccggatca

2101 aagagttcct ccgccgctgg acctaccaag gcaacgctat gttctcttgc ttttgtcagc

2161 aagatagcca gatcaatgtc gatcgtggct ggctcgaaga tacctgcaag aatgtcattg

2221 cgctgccatt ctccaaattg cagttcgcgc ttagctggat aacgccacgg aatgatgtcg

2281 tcgtgcacaa caatggtgac ttctacagcg cggagaatct cgctctctcc aggggaagcc

2341 gaagtttcca aaaggtcgtt gatcaaagct cgccgcgttg tttcatcaag ccttacggtc

2401 accgtaacca gcaaatcaat atcactgtgt ggcttcaggc cgccatccac tgcggagccg

2461 tacaaatgta cggccagcaa cgtcggttcg agatggcgct cgatgacgcc aactacctct

2521 gatagttgag tcgatacttc ggcgatcacc gcttccctca tgatgtttaa ctttgtttta

2581 gggcgactgc cctgctgcgt aacatcgttg ctgctccata acatcaaaca tcgacccacg

2641 gcgtaacgcg cttgctgctt ggatgcccga ggcatagact gtaccccaaa aaaacagtca

2701 taacaagcca tgaaaaccgc cactgcgccg ttaccaccgc tgcgttcggt caaggttctg

2761 gaccagttgc gtgagcgcat acgctacttg cattacagct tacgaaccga acaggcttat

2821 gtccactggg ttcgtgcctt catccgtttc cacggtgtgc gtcacccggc aaccttgggc

2881 agcagcgaag tcgaggcatt tctgtcctgg ctggcgaacg agcgcaaggt ttcggtctcc

2941 acgcatcgtc aggcattggc ggccttgctg ttcttctaga tctagaggat ctgtaatccg

3001 ggcagcgcaa cggaacattc atcagtgtaa aaatggaatc aataaagccc tgcgcagcgc

3061 gcagggtcag cctgaatacg cgtttaatga ccagcacagt cgtgatggca aggtcagaat

3121 agcgctgagg tctgcctcgt gaagaaggtg ttgctgactc ataccaggcc tgaatcgccc

3181 catcatccag ccagaaagtg agggagccac ggttgatgag agctttgttg taggtggacc

3241 agttggtgat tttgaacttt tgctttgcca cggaacggtc tgcgttgtcg ggaagatgcg

3301 tgatctgatc cttcaactca gcaaaagttc gatttattca acaaagccgc cgtcccgtca

3361 agtcagcgta atgctctgcc agtgttacaa ccaattaacc aattctgatt agaaaaactc

3421 atcgagcatc aaatgaaact gcaatttatt catatcagga ttatcaatac catatttttg

3481 aaaaagccgt ttctgtaatg aaggagaaaa ctcaccgagg cagttccata ggatggcaag

3541 atcctggtat cggtctgcga ttccgactcg tccaacatca atacaaccta ttaatttccc

3601 ctcgtcaaaa ataaggttat caagtgagaa atcaccatga gtgacgactg aatccggtga

3661 gaatggcaaa agcttatgca tttctttcca gacttgttca acaggccagc cattacgctc

3721 gtcatcaaaa tcactcgcat caaccaaacc gttattcatt cgtgattgcg cctgagcgag

3781 acgaaatacg cgatcgctgt taaaaggaca attacaaaca ggaatcgaat gcaaccggcg

3841 caggaacact gccagcgcat caacaatatt ttcacctgaa tcaggatatt cttctaatac

3901 ctggaatgct gttttcccgg ggatcgcagt ggtgagtaac catgcatcat caggagtacg

3961 gataaaatgc ttgatggtcg gaagaggcat aaattccgtc agccagttta gtctgaccat

4021 ctcatctgta acatcattgg caacgctacc tttgccatgt ttcagaaaca actctggcgc

4081 atcgggcttc ccatacaatc gatagattgt cgcacctgat tgcccgacat tatcgcgagc

4141 ccatttatac ccatataaat cagcatccat gttggaattt aatcgcggcc tcgagcaaga

4201 cgtttcccgt tgaatatggc tcataacacc ccttgtatta ctgtttatgt aagcagacag

4261 ttttattgtt catgatgata tatttttatc ttgtgcaatg taacatcaga gattttgaga

4321 cacaacgtgg ctttgttgaa taaatcgaac ttttgctgag ttgaaggatc agatcacgca

4381 tcttcccgac aacgcagacc gttccgtggc aaagcaaaag ttcaaaatca ccaactggtc

4441 cacctacaac aaagctctca tcaaccgtgg ctccctcact ttctggctgg atgatggggc

4501 gattcaggcc tggtatgagt cagcaacacc ttcttcacga ggcagacctc agcgctattc

4561 tgaccttgcc atcacgactg tgctggtcat taaacgcgta ttcaggctga ccctgcgcgc

4621 tgcgcagggc tttattgatt ccatttttac actgatgaat gttccgttgc gctgcccgga

4681 ttacagctga aagcgaccag gtgctcggcg tggcaagact cgcagcgaac ccgtagaaag

4741 ccatgctcca gccgcccgca ttggagaaat tcttcaaatt cccgttgcac atagcccggc

4801 aattcctttc cctgctctgc cataagcgca gcgaatgccg ggtaatactc gtcaacgatc

4861 tgatagagaa gggtttgctc gggtcggtgg ctctggtaac gaccagtatc ccgatcccgg

4921 ctggccgtcc tggccgccac atgaggcatg ttccgcgtcc ttgcaatact gtgtttacat

4981 acagtctatc gcttagcgga aagttctttt accctcagcc gaaatgcctg ccgttgctag

5041 acattgccag ccagtgcccg tcactcccgt actaactgtc acgaacccct gcaataactg

5101 tcacgccccc ctgcaataac tgtcacgaac ccctgcaata actgtcacgc ccccaaacct

5161 gcaaacccag caggggcggg ggctggcggg gtgttggaaa aatccatcca tgattatcta

5221 agaataatcc actaggcgcg gttatcagcg cccttgtggg gcgctgctgc ccttgcccaa

5281 tatgcccggc cagaggccgg atagctggtc tattcgctgc gctaggctac acaccgcccc

5341 accgctgcgc ggcaggggga aaggcgggca aagcccgcta aaccccacac caaaccccgc

5401 agaaatacgc tggagcgctt ttagccgctt tagcggcctt tccccctacc cgaagggtgg

5461 gggcgcgtgt gcagccccgc agggcctgtc tcggtcgatc attcagcccg gctcatcctt

5521 ctggcgtggc ggcagaccga acaaggcgcg gtcgtggtcg cgttcaaggt acgcatccat

5581 tgccgccatg agccgatcct ccggccactc gctgctgttc accttggcca aaatcatggc

5641 ccccaccagc accttgcgcc ttgtttcgtt cttgcgctct tgctgctgtt cccttgcccg

5701 cacccgctga atttcggcat tgattcgcgc tcgttgttct tcgagcttgg ccagccgatc

5761 cgccgccttg ttgctcccct taaccatctt gacaccccat tgttaatgtg ctgtctcgta

5821 ggctatcatg gaggcacagc ggcggcaatc ccgaccctac tttgtagggg agggcgcact

5881 taccggtttc tcttcgagaa actggcctaa cggccaccct tcgggcggtg cgctctccga

5941 gggccattgc atggagccga aaagcaaaag caacagcgag gcagcatggc gatttatcac

6001 cttacggcga aaaccggcag caggtcgggc ggccaatcgg ccagggccaa ggccgactac

6061 atccagcgcg aaggcaagta tgcccgcgac atggatgaag tcttgcacgc cgaatccggg

6121 cacatgccgg agttcgtcga gcggcccgcc gactactggg atgctgccga cctgtatgaa

6181 cgcgccaatg ggcggctgtt caaggaggtc gaatttgccc tgccggtcga gctgaccctc

6241 gaccagcaga aggcgctggc gtccgagttc gcccagcacc tgaccggtgc cgagcgcctg

6301 ccgtatacgc tggccatcca tgccggtggc ggcgagaacc cgcactgcca cctgatgatc

6361 tccgagcgga tcaatgacgg catcgagcgg cccgccgctc agtggttcaa gcggtacaac

6421 ggcaagaccc cggagaaggg cggggcacag aagaccgaag cgctcaagcc caaggcatgg

6481 cttgagcaga cccgcgaggc atgggccgac catgccaacc gggcattaga gcgggctggc

6541 cacgacgccc gcattgacca cagaacactt gaggcgcagg gcatcgagcg cctgcccggt

6601 gttcacctgg ggccgaacgt ggtggagatg gaaggccggg gcatccgcac cgaccgggca

6661 gacgtggccc tgaacatcga caccgccaac gcccagatca tcgacttaca ggaataccgg

6721 gaggcaatag accatgaacg caatcgacag agtgaagaaa tccagaggca tcaacgagtt

6781 agcggagcag atcgaaccgc tggcccagag catggcgaca ctggccgacg aagcccggca

6841 ggtcatgagc cagacccagc aggccagcga ggcgcaggcg gcggagtggc tgaaagccca

6901 gcgccagaca ggggcggcat gggtggagct ggccaaagag ttgcgggagg tagccgccga

6961 ggtgagcagc gccgcgcaga gcgcccggag cgcgtcgcgg gggtggcact ggaagctatg

7021 gctaaccgtg atgctggctt ccatgatgcc tacggtggtg ctgctgatcg catcgttgct

7081 cttgctcgac ctgacgccac tgacaaccga ggacggctcg atctggctgc gcttggtggc

7141 ccgatgaaga acgacaggac tttgcaggcc ataggccgac agctcaaggc catgggctgt

7201 gagcgcttcg atatcggcgt cagggacgcc accaccggcc agatgatgaa ccgggaatgg

7261 tcagccgccg aagtgctcca gaacacgcca tggctcaagc ggatgaatgc ccagggcaat

7321 gacgtgtata tcaggcccgc cgagcaggag cggcatggtc tggtgctggt ggacgacctc

7381 agcgagtttg acctggatga catgaaagcc gagggccggg agcctgccct ggtagtggaa

7441 accagcccga agaactatca ggcatgggtc aaggtggccg acgccgcagg cggtgaactt

7501 cgggggcaga ttgcccggac gctggccagc gagtacgacg ccgacccggc cagcgccgac

7561 agccgccact atggccgctt ggcgggcttc accaaccgca aggacaagca caccacccgc

7621 gccggttatc agccgtgggt gctgctgcgt gaatccaagg gcaagaccgc caccgctggc

7681 ccggcgctgg tgcagcaggc tggccagcag atcgagcagg cccagcggca gcaggagaag

7741 gcccgcaggc tggccagcct cgaactgccc gagcggcagc ttagccgcca ccggcgcacg

7801 gcgctggacg agtaccgcag cgagatggcc gggctggtca agcgcttcgg tgatgacctc

7861 agcaagtgcg actttatcgc cgcgcagaag ctggccagcc ggggccgcag tgccgaggaa

7921 atcggcaagg ccatggccga ggccagccca gcgctggcag agcgcaagcc cggccacgaa

7981 gcggattaca tcgagcgcac cgtcagcaag gtcatgggtc tgcccagcgt ccagcttgcg

8041 cgggccgagc tggcacgggc accggcaccc cgccagcgag gcatggacag gggcgggcca

8101 gatttcagca tgtagtgctt gcgttggtac tcacgcctgt tatactatga gtactcacgc

8161 acagaagggg gttttatgga atacgaaaaa agcgcttcag ggtcggtcta cctgatcaaa

8221 agtgacaagg gctattggtt gcccggtggc tttggttata cgtcaaacaa ggccgaggct

8281 ggccgctttt cagtcgctga tatggccagc cttaaccttg acggctgcac cttgtccttg

8341 ttccgcgaag acaagccttt cggccccggc aagtttctcg gtgactgata tgaaagacca

8401 aaaggacaag cagaccggcg acctgctggc cagccctgac gctgtacgcc aagcgcgata

8461 tgccgagcgc atgaaggcca aagggatgcg tcagcgcaag ttctggctga ccgacgacga

8521 atacgaggcg ctgcgcgagt gcctggaaga actcagagcg gcgcagggcg ggggtagtga

8581 ccccgccagc gcctaaccac caactgcctg caaaggaggc aatcaatggc tacccataag

8641 cctatcaata ttctggaggc gttcgcagca gcgccgccac cgctggacta cgttttgccc

8701 aacatggtgg ccggtacggt cggggcgctg gtgtcgcccg gtggtgccgg taaatccatg

8761 ctggccctgc aactggccgc acagattgca ggcgggccgg atctgctgga ggtgggcgaa

8821 ctgcccaccg gcccggtgat ctacctgccc gccgaagacc cgcccaccgc cattcatcac

8881 cgcctgcacg cccttggggc gcacctcagc gccgaggaac ggcaagccgt ggctgacggc

8941 ctgctgatcc agccgctgat cggcagcctg cccaacatca tggccccgga gtggttcgac

9001 ggcctcaagc gcgccgccga gggccgccgc ctgatggtgc tggacacgct gcgccggttc

9061 cacatcgagg aagaaaacgc cagcggcccc atggcccagg tcatcggtcg catggaggcc

9121 atcgccgccg ataccgggtg ctctatcgtg ttcctgcacc atgccagcaa gggcgcggcc

9181 atgatgggcg caggcgacca gcagcaggcc agccggggca gctcggtact ggtcgataac

9241 atccgctggc agtcctacct gtcgagcatg accagcgccg aggccgagga atggggtgtg

9301 gacgacgacc agcgccggtt cttcgtccgc ttcggtgtga gcaaggccaa ctatggcgca

9361 ccgttcgctg atcggtggtt caggcggcat gacggcgggg tgctcaagcc cgccgtgctg

9421 gagaggcagc gcaagagcaa gggggtgccc cgtggtgaag cctaagaaca agcacagcct

9481 cagccacgtc cggcacgacc cggcgcactg tctggccccc ggcctgttcc gtgccctcaa

9541 gcggggcgag cgcaagcgca gcaagctgga cgtgacgtat gactacggcg acggcaagcg

9601 gatcgagttc agcggcccgg agccgctggg cgctgatgat ctgcgcatcc tgcaagggct

9661 ggtggccatg gctgggccta atggcctagt gcttggcccg gaacccaaga ccgaaggcgg

9721 acggcagctc cggctgttcc tggaacccaa gtgggaggcc gtcaccgctg aatgccatgt

9781 ggtcaaaggt agctatcggg cgctggcaaa ggaaatcggg gcagaggtcg atagtggtgg

9841 ggcgctcaag cacatacagg actgcatcga gcgcctttgg aaggtatcca tcatcgccca

9901 gaatggccgc aagcggcagg ggtttcggct gctgtcggag tacgccagcg acgaggcgga

9961 cgggcgcctg tacgtggccc tgaacccctt gatcgcgcag gccgtcatgg gtggcggcca

10021 gcatgtgcgc atcagcatgg acgaggtgcg ggcgctggac agcgaaaccg cccgcctgct

10081 gcaccagcgg ctgtgtggct ggatcgaccc cggcaaaacc ggcaaggctt ccatagatac

10141 cttgtgcggc tatgtctggc cgtcagaggc cagtggttcg accatgcgca agcgccgcca

10201 gcgggtgcgc gaggcgttgc cggagctggt cgcgctgggc tggacggtaa ccgagttcgc

10261 ggcgggcaag tacgacatca cccggcccaa ggcggcaggc tgaccccccc cactctattg

10321 taaacaagac atttttatct tttatattca atggcttatt ttcctgctaa ttggtaatac

10381 catgaaaaat accatgctca gaaaaggctt aacaatattt tgaaaaattg cctactgagc

10441 gctgccgcac agctccatag gccgctttcc tggctttgct tccagatgta tgctcttctg

10501 ct

//

**Inserts in template vectors for cloning**

**pRhon5Hi-2-H6-THAS1-SQS1-SQE1**

LOCUS H6-THAS-SQS-SQE 5372 bp DNA linear SYN 25-OCT-2017

KEYWORDS pRhon5Hi-2-H6-THAS1-SQS1-SQE1

FEATURES Location/Qualifiers

regulatory 29..34

/regulatory_class=ribosome_binding_site

/gene="pRhotHi-2 RBS"

/product="RBS of vector pRhotHi-2"

CDS 43..66

/gene="His6"

/product="N-terminally fused hexahistidin-Tag"

/translation="MHHHHHHW"

CDS 64..2337

/gene="'THAS1"

/product="A. thaliana thalianol synthase THAS1"

/translation="WRLRTGPKAGEDTHLFTTNNYAGRQIWEFDANAGSPQEIAEVED

ARHKFSDNTSRFKTTADLLWRMQFLREKKFEQKIPRVIIEDARKIKYEDAKTALKRGL

LYFTALQADDGHWPAENSGPNFYTPPFLICLYITGHLEKIFTPEHVKELLRHIYNMQN

EDGGWGLHVESHSVMFCTVINYVCLRIVGEEVGHDDQRNGCAKAHKWIMDHGGATYTP

LIGKALLSVLGVYDWSGCNPIPPEFWLLPSSFPVNGGTLWIYLRDTFMGLSYLYGKKF

VAPPTPLILQLREELYPEPYAKINWTQTRNRCGKEDLYYPRSFLQDLFWKSVHMFSES

ILDRWPLNKLIRQRALQSTMALIHYHDESTRYITGGCLPKAFHMLACWIEDPKSDYFK

KHLARVREYIWIGEDGLKIQSFGSQLWDTALSLHALLDGIDDHDVDDEIKTTLVKGYD

YLKKSQITENPRGDHFKMFRHKTKGGWTFSDQDQGWPVSDCTAESLECCLFFESMPSE

LIGKKMDVEKLYDAVDYLLYLQSDNGGIAAWQPVEGKAWLELLNIMIFRYVECTGSAI

AALTQFNKQFPGYKNVEVKRFITKAAKYIEDMQTVDGSWYGNWGVCFIYGTFFAVRGL

VAAGKTYSNCEAIRKAVRFLLDTQNPEGGWGESFLSCPSKKYTPLKGNSTNVVQTAQA

LMVLIMGDQMERDPLPVHRAAQVLINSQLDNGDFPQQEIMGTFMRTVMLHFPTYRNTF

SLWALTHYTHALRRLLP"

regulatory 2338..2356

/regulatory_class=ribosome_binding_site

/gene="5' nifK"

/product="5' UTR of R. capsulatus nifK incl. RBS"

CDS 2360..3592

/gene="SQS1"

/product="A. thaliana squalene synthase SQS1"

/translation="MGSLGTMLRYPDDIYPLLKMKRAIEKAEKQIPPEPHWGFCYSML

HKVSRSFSLVIQQLNTELRNAVCVFYLVLRALDTVEDDTSIPTDEKVPILIAFHRHIY

DTDWHYSCGTKEYKILMDQFHHVSAAFLELEKGYQEAIEEITRRMGAGMAKFICQEVE

TVDDYDEYCHYVAGLVGLGLSKLFLAAGSEVLTPDWEAISNSMGLFLQKTNIIRDYLE

DINEIPKSRMFWPREIWGKYADKLEDLKYEENTNKSVQCLNEMVTNALMHIEDCLKYM

VSLRDPSIFRFCAIPQIMAIGTLALCYNNEQVFRGVVKLRRGLTAKVIDRTKTMADVY

GAFYDFSCMLKTKVDKNDPNASKTLNRLEAVQKLCRDAGVLQNRKSYVNDKGQPNSVF

IIMVVILLAIVFAYLRAN"

regulatory 3601..3606

/regulatory_class=ribosome_binding_site

/gene="RBS"

/product="RBS of pET vector series"

CDS 3615..5210

/gene="SQE1"

/product="A. thaliana squalene epoxidase SQE1"

/translation="MESQLWNWILPLLISSLLISFVAFYGFFVKPKRNGLRHDRKTVS

TVTSDVGSVNITGDTVADVIVVGAGVAGSALAYTLGKDKRRVHVIERDLSEPDRIVGE

LLQPGGYLKLLELGIEDCVEEIDAQRVYGYALFKNGKRIRLAYPLEKFHEDVSGRSFH

NGRFIQRMREKAASLPNVQLEQGTVLSLLEENGTIKGVRYKNKAGEEQTAFAALTIVC

DGCFSNLRRSLCNPQVEVPSCFVGLVLENCNLPYANHGHVVLADPSPILMYPISSTEV

RCLVDVPGQKVPSIANGEMKNYLKTVVAPQMPHEVYDSFIAAVDKGNIKSMPNRSMPA

SPYPTPGALLMGDAFNMRHPLTGGGMTVALADIVVLRNLLRPLRDLSDGASLCKYLES

FYTLRKPVAATINTLANALYQVFCSSENEARNEMREACFDYLGLGGMCTSGPVSLLSG

LNPRPLTLVCHFFAVAVYGVIRLLIPFPSPKRIWLGAKLISGASGIIFPIIKAEGVRQ

MFFPATVPAYYYKAPTVGETKCS"

regulatory 5325..5372

/regulatory_class=terminator

/gene="T7 terminator"

/product="bacteriophage T7 transcription terminator"

ORIGIN

1 tctagaaata attttgttta actttaagaa ggagatatac atatgcacca tcaccatcat

61 cactggcgcc tgcgcaccgg cccgaaagcg ggcgaagata cccatctgtt caccaccaac

121 aactatgcgg gccgccagat ctgggaattc gatgcgaacg cgggcagccc gcaggaaatc

181 gcggaagtgg aagatgcgcg ccataaattc agcgataaca ccagccgctt caaaaccacc

241 gcggatctgc tgtggcgcat gcagttcctg cgcgaaaaaa aattcgaaca gaaaatcccg

301 cgcgtgatca tcgaagatgc gcgcaaaatc aaatatgaag atgcgaaaac cgcgctgaaa

361 cgcggcctgc tgtatttcac cgcgctgcag gcggatgatg gccattggcc ggcggaaaac

421 agcggcccga acttctatac cccgccgttc ctgatctgcc tgtatatcac cggccatctg

481 gaaaaaatct tcaccccgga acatgtgaaa gaactgctgc gccatatcta taacatgcag

541 aacgaagatg gcggctgggg cctgcatgtg gaaagccata gcgtgatgtt ctgcaccgtg

601 atcaactatg tgtgcctgcg catcgtgggc gaagaagtgg gccatgatga tcagcgcaac

661 ggctgcgcga aagcgcataa atggatcatg gatcatggcg gcgcgaccta taccccgctg

721 atcggcaaag cgctgctgag cgtgctgggc gtgtatgatt ggagcggctg caacccgatc

781 ccgccggaat tctggctgct gccgagcagc ttcccggtga acggcggcac cctgtggatc

841 tatctgcgcg ataccttcat gggcctgagc tatctgtatg gcaaaaaatt cgtggcgccg

901 ccgaccccgc tgatcctgca gctgcgcgaa gaactgtatc cggaaccgta tgcgaaaatc

961 aactggaccc agacccgcaa ccgctgcggc aaagaagatc tgtattatcc gcgcagcttc

1021 ctgcaggatc tgttctggaa aagcgtgcac atgttcagcg aaagcatcct ggatcgctgg

1081 ccgctgaaca aactgatccg ccagcgcgcg ctgcagagca ccatggcgct gatccattat

1141 catgatgaaa gcacccgcta tatcaccggc ggctgcctgc cgaaagcgtt ccacatgctg

1201 gcgtgctgga tcgaagatcc gaaaagcgat tatttcaaaa aacatctggc gcgcgtgcgc

1261 gaatatatct ggatcggcga agatggcctg aaaatccaga gcttcggcag ccagctgtgg

1321 gataccgcgc tgagcctgca tgcgctgctg gatggcatcg atgatcatga tgtggatgat

1381 gaaatcaaaa ccaccctggt gaaaggctat gattatctga aaaaaagcca gatcaccgaa

1441 aacccgcgcg gcgatcattt caaaatgttc cgccataaaa ccaaaggcgg ctggaccttc

1501 agcgatcagg atcagggctg gccggtgagc gattgcaccg cggaaagcct ggaatgctgc

1561 ctgttcttcg aaagcatgcc gagcgaactg atcggcaaaa aaatggatgt ggaaaaactg

1621 tatgatgcgg tggattatct gctgtatctg cagagcgata acggcggcat cgcggcgtgg

1681 cagccggtgg aaggcaaagc gtggctggaa ctgctgaaca tcatgatctt ccgctatgtg

1741 gaatgcaccg gcagcgcgat cgcggcgctg acccagttca acaaacagtt cccgggctat

1801 aaaaacgtgg aagtgaaacg cttcatcacc aaagcggcga aatatatcga agatatgcag

1861 accgtggatg gcagctggta tggcaactgg ggcgtgtgct tcatctatgg caccttcttt

1921 gcggtgcgcg gcctggtggc ggcgggcaaa acctatagca actgcgaagc gatccgcaaa

1981 gcggtgcgct tcctgctgga tacccagaac ccggaaggcg gctggggcga gagcttcctg

2041 agctgcccga gcaaaaaata taccccgctg aaaggcaaca gcaccaacgt ggtgcagacc

2101 gcgcaggcgc tgatggtgct gatcatgggc gatcagatgg aacgcgatcc gctgccggtg

2161 catcgcgcgg cgcaggtgct gatcaacagc cagctggata acggcgattt cccgcagcag

2221 gaaatcatgg gcaccttcat gcgcaccgtg atgctgcatt tcccgaccta tcgcaacacc

2281 ttcagcctgt gggcgctgac ccattatacc catgcgctgc gccgcctgct gccgtaagcc

2341 tttgacaagg aattgacata tgggcagcct gggcaccatg ctgcgctatc cggatgatat

2401 ctatccgctg ctgaaaatga aacgcgcgat cgaaaaagcg gaaaaacaga tcccgccgga

2461 accgcattgg ggcttctgct atagcatgct gcataaagtg agccgcagct tcagcctggt

2521 gatccagcag ctgaacaccg aactgcgcaa cgcggtgtgc gtgttctatc tggtgctgcg

2581 cgcgctggat accgtggaag atgataccag catcccgacc gatgaaaaag tgccgatcct

2641 gatcgcgttc catcgccata tctatgatac cgattggcat tatagctgcg gcaccaaaga

2701 atataaaatc ctgatggatc agttccatca tgtgagcgcg gcgttcctgg aactggaaaa

2761 aggctatcag gaagcgatcg aagaaatcac ccgccgcatg ggcgcgggca tggcgaaatt

2821 catctgccag gaagtggaaa ccgtggatga ttatgatgaa tattgccatt atgtggcggg

2881 cctggtgggc ctgggcctga gcaaactgtt cctggcggcg ggcagcgaag tgctgacccc

2941 ggattgggaa gcgatcagca acagcatggg cctgttcctg cagaaaacca acatcatccg

3001 cgattatctg gaagatatca acgaaatccc gaaaagccgc atgttctggc cgcgcgaaat

3061 ctggggcaaa tatgcggata aactggaaga tctgaaatat gaagaaaaca ccaacaaaag

3121 cgtgcagtgc ctgaacgaaa tggtgaccaa cgcgctgatg catatcgaag attgcctgaa

3181 atatatggtg agcctgcgcg atccgagcat cttccgcttc tgcgcgatcc cgcagatcat

3241 ggcgatcggc accctggcgc tgtgctataa caacgaacag gtgttccgcg gcgtggtgaa

3301 actgcgccgc ggcctgaccg cgaaagtgat cgatcgcacc aaaaccatgg cggatgtgta

3361 tggcgcgttc tatgatttca gctgcatgct gaaaaccaaa gtggataaaa acgatccgaa

3421 cgcgagcaaa accctgaacc gcctggaagc ggtgcagaaa ctgtgccgcg atgcgggcgt

3481 gctgcagaac cgcaaaagct atgtgaacga taaaggccag ccgaacagcg tgttcatcat

3541 catggtggtg atcctgctgg cgatcgtgtt cgcgtatctg cgcgcgaact aactcgagat

3601 aaggagatat acacatggaa agccagctgt ggaactggat cctgccgctg ctgatcagca

3661 gcctgctgat cagcttcgtg gcgttctatg gcttcttcgt gaaaccgaaa cgcaacggcc

3721 tgcgccatga tcgcaaaacc gtgagcaccg tgaccagcga tgtgggcagc gtgaacatca

3781 ccggcgatac cgtggcggat gtgatcgtgg tgggcgcggg cgtggcgggc agcgcgctgg

3841 cgtataccct gggcaaagat aaacgccgcg tgcatgtgat cgaacgcgat ctgagcgaac

3901 cggatcgcat cgtgggcgaa ctgctgcagc cgggcggcta tctgaaactg ctggaactgg

3961 gcatcgaaga ttgcgtggaa gaaatcgatg cgcagcgcgt gtatggctat gcgctgttca

4021 aaaacggcaa acgcatccgc ctggcgtatc cgctggaaaa attccatgaa gatgtgagcg

4081 gccgcagctt ccataacggc cgcttcatcc agcgcatgcg cgaaaaagcg gcgagcctgc

4141 cgaacgtgca gctggaacag ggcaccgtgc tgagcctgct ggaagaaaac ggcaccatca

4201 aaggcgtgcg ctataaaaac aaagcgggcg aagaacagac cgcgttcgcg gcgctgacca

4261 tcgtgtgcga tggctgcttc agcaacctgc gccgcagcct gtgcaacccg caggtggaag

4321 tgccgagctg cttcgtgggc ctggtgctgg aaaactgcaa cctgccgtat gcgaaccatg

4381 gccatgtggt gctggcggat ccgagcccga tcctgatgta tccgatcagc agcaccgaag

4441 tgcgctgcct ggtggatgtg ccgggccaga aagtgccgag catcgcgaac ggcgaaatga

4501 aaaactatct gaaaaccgtg gtggcgccgc agatgccgca tgaagtgtat gatagcttca

4561 tcgcggcggt ggataaaggc aacatcaaaa gcatgccgaa ccgcagcatg ccggcgagcc

4621 cgtatccgac cccgggcgcg ctgctgatgg gcgatgcgtt caacatgcgc catccgctga

4681 ccggcggcgg catgaccgtg gcgctggcgg atatcgtggt gctgcgcaac ctgctgcgcc

4741 cgctgcgcga tctgagcgat ggcgcgagcc tgtgcaaata tctggagagc ttctataccc

4801 tgcgcaaacc ggtggcggcg accatcaaca ccctggcgaa cgcgctgtat caggtgttct

4861 gcagcagcga aaacgaagcg cgcaacgaaa tgcgcgaagc gtgcttcgat tatctgggcc

4921 tgggcggcat gtgcaccagc ggcccggtga gcctgctgag cggcctgaac ccgcgcccgc

4981 tgaccctggt gtgccatttc ttcgcggtgg cggtgtatgg cgtgatccgc ctgctgatcc

5041 cgttcccgag cccgaaacgc atctggctgg gcgcgaaact gatcagcggc gcgagcggca

5101 tcatcttccc gatcatcaaa gcggaaggcg tgcgccagat gttcttcccg gcgaccgtgc

5161 cggcgtatta ttataaagcg ccgaccgtgg gcgaaaccaa atgcagctaa acgcgttata

5221 agcttgcggc cgcactcgag caccaccacc accaccactg agatccggct gctaacaaag

5281 cccgaaagga agctgagttg gctgctgcca ccgctgagca ataactagca taaccccttg

5341 gggcctctaa acgggtcttg aggggttttt tg

//

**pRhofHi-2- SQS1-SQE1-MRN1**

LOCUS SQS1-SQE1-MRN1 5318 bp DNA linear SYN 25-OCT-2017

KEYWORDS pRhofHi-2-SQS1-SQE1-MRN1

FEATURES Location/Qualifiers

CDS 4..1236

/gene="SQS1"

/product="A. thaliana squalene synthase SQS1"

/translation="MGSLGTMLRYPDDIYPLLKMKRAIEKAEKQIPPEPHWGFCYSML

HKVSRSFSLVIQQLNTELRNAVCVFYLVLRALDTVEDDTSIPTDEKVPILIAFHRHIY

DTDWHYSCGTKEYKILMDQFHHVSAAFLELEKGYQEAIEEITRRMGAGMAKFICQEVE

TVDDYDEYCHYVAGLVGLGLSKLFLAAGSEVLTPDWEAISNSMGLFLQKTNIIRDYLE

DINEIPKSRMFWPREIWGKYADKLEDLKYEENTNKSVQCLNEMVTNALMHIEDCLKYM

VSLRDPSIFRFCAIPQIMAIGTLALCYNNEQVFRGVVKLRRGLTAKVIDRTKTMADVY

GAFYDFSCMLKTKVDKNDPNASKTLNRLEAVQKLCRDAGVLQNRKSYVNDKGQPNSVF

IIMVVILLAIVFAYLRAN"

regulatory 1245..1250

/regulatory_class=ribosome_binding_site

/gene="pET RBS"

/product="RBS of pET vector series"

CDS 1259..2854

/gene="SQE1"

/product="A. thaliana squalene epoxidase SQE1"

/translation="MESQLWNWILPLLISSLLISFVAFYGFFVKPKRNGLRHDRKTVS

TVTSDVGSVNITGDTVADVIVVGAGVAGSALAYTLGKDKRRVHVIERDLSEPDRIVGE

LLQPGGYLKLLELGIEDCVEEIDAQRVYGYALFKNGKRIRLAYPLEKFHEDVSGRSFH

NGRFIQRMREKAASLPNVQLEQGTVLSLLEENGTIKGVRYKNKAGEEQTAFAALTIVC

DGCFSNLRRSLCNPQVEVPSCFVGLVLENCNLPYANHGHVVLADPSPILMYPISSTEV

RCLVDVPGQKVPSIANGEMKNYLKTVVAPQMPHEVYDSFIAAVDKGNIKSMPNRSMPA

SPYPTPGALLMGDAFNMRHPLTGGGMTVALADIVVLRNLLRPLRDLSDGASLCKYLES

FYTLRKPVAATINTLANALYQVFCSSENEARNEMREACFDYLGLGGMCTSGPVSLLSG

LNPRPLTLVCHFFAVAVYGVIRLLIPFPSPKRIWLGAKLISGASGIIFPIIKAEGVRQ

MFFPATVPAYYYKAPTVGETKCS"

regulatory 2862..2873

/regulatory_class=ribosome_binding_site

/gene="BBa_0034 RBS"

/product="Biobrick RBS BBa_0034"

CDS 2880..5165

/gene="MRN1"

/product="A. thaliana marneral synthase MRN1"

/translation="MWRLRIGAEARQDPHLFTTNNFAGRQIWEFDANGGSPEELAEVE

EARLNFANNKSRFKASPDLFWRRQFLREKKFEQKIPRVRIEDAEKITYEDAKTALRRG

VLYYAACQANDGHWPSEVSGSMFLDAPFVICLYITGHLEKIFTLEHVKELLRYMYNTQ

NEDGGWGLDVESHSVMFCTVLNYICLRILGVEPDHDGQKSACARARKWILDHGGATYA

PMVAKAWLSVLGVYDWSGCKPLPPEIWMLPSFSPINGGTLWIYIRDLLMGMSYLYGKK

FVATPTALILQLREELYPQPYSKIIWSKARNRCAKEDLLYPKSFGQDLFWEGVHMLSE

NIINRWPLNKFVRQRALRTTMELVHYHDETTHYITGACVAKPFHMLACWVEDPDGDYF

KKHLARVPDFIWIAEDGLKFQLMGMQSWNAALSLQVMLAANMDDEIRSTLIKGYDFLK

QSQISENPQGDHLKMFRDITKGGWTFQDREQGLPISDGTAESIECCIHFHRMPSEFIG

EKMDVEKLYDAVNFLIYLQSDNGGMPVWEPAPGKKWLEWLSPVEHVENTVVEQEYLEC

TGSVIAGLVCFKKEFPDHRPKEIEKLIKKGLKYIEDLQMPDGSWYGNWGVCFTYGTLF

AVRGLAAAGKTFGNSEAIRRAVQFILNTQNAEGGWGESALSCPNKKYIPSKGNVTNVV

NTGQAMMVLLIGGQMERDPSPVHRAAKVLINSQLDIGDFPQQERRGIYMNMLLHYPTY

RNMFSLWALALYTNALRLLVS"

regulatory 5271..5318

/regulatory_class=terminator

/gene="T7 terminator"

/product="bacteriophage T7 transcription terminator"

ORIGIN

1 catatgggca gcctgggcac catgctgcgc tatccggatg atatctatcc gctgctgaaa

61 atgaaacgcg cgatcgaaaa agcggaaaaa cagatcccgc cggaaccgca ttggggcttc

121 tgctatagca tgctgcataa agtgagccgc agcttcagcc tggtgatcca gcagctgaac

181 accgaactgc gcaacgcggt gtgcgtgttc tatctggtgc tgcgcgcgct ggataccgtg

241 gaagatgata ccagcatccc gaccgatgaa aaagtgccga tcctgatcgc gttccatcgc

301 catatctatg ataccgattg gcattatagc tgcggcacca aagaatataa aatcctgatg

361 gatcagttcc atcatgtgag cgcggcgttc ctggaactgg aaaaaggcta tcaggaagcg

421 atcgaagaaa tcacccgccg catgggcgcg ggcatggcga aattcatctg ccaggaagtg

481 gaaaccgtgg atgattatga tgaatattgc cattatgtgg cgggcctggt gggcctgggc

541 ctgagcaaac tgttcctggc ggcgggcagc gaagtgctga ccccggattg ggaagcgatc

601 agcaacagca tgggcctgtt cctgcagaaa accaacatca tccgcgatta tctggaagat

661 atcaacgaaa tcccgaaaag ccgcatgttc tggccgcgcg aaatctgggg caaatatgcg

721 gataaactgg aagatctgaa atatgaagaa aacaccaaca aaagcgtgca gtgcctgaac

781 gaaatggtga ccaacgcgct gatgcatatc gaagattgcc tgaaatatat ggtgagcctg

841 cgcgatccga gcatcttccg cttctgcgcg atcccgcaga tcatggcgat cggcaccctg

901 gcgctgtgct ataacaacga acaggtgttc cgcggcgtgg tgaaactgcg ccgcggcctg

961 accgcgaaag tgatcgatcg caccaaaacc atggcggatg tgtatggcgc gttctatgat

1021 ttcagctgca tgctgaaaac caaagtggat aaaaacgatc cgaacgcgag caaaaccctg

1081 aaccgcctgg aagcggtgca gaaactgtgc cgcgatgcgg gcgtgctgca gaaccgcaaa

1141 agctatgtga acgataaagg ccagccgaac agcgtgttca tcatcatggt ggtgatcctg

1201 ctggcgatcg tgttcgcgta tctgcgcgcg aactaactcg agataaggag atatacacat

1261 ggaaagccag ctgtggaact ggatcctgcc gctgctgatc agcagcctgc tgatcagctt

1321 cgtggcgttc tatggcttct tcgtgaaacc gaaacgcaac ggcctgcgcc atgatcgcaa

1381 aaccgtgagc accgtgacca gcgatgtggg cagcgtgaac atcaccggcg ataccgtggc

1441 ggatgtgatc gtggtgggcg cgggcgtggc gggcagcgcg ctggcgtata ccctgggcaa

1501 agataaacgc cgcgtgcatg tgatcgaacg cgatctgagc gaaccggatc gcatcgtggg

1561 cgaactgctg cagccgggcg gctatctgaa actgctggaa ctgggcatcg aagattgcgt

1621 ggaagaaatc gatgcgcagc gcgtgtatgg ctatgcgctg ttcaaaaacg gcaaacgcat

1681 ccgcctggcg tatccgctgg aaaaattcca tgaagatgtg agcggccgca gcttccataa

1741 cggccgcttc atccagcgca tgcgcgaaaa agcggcgagc ctgccgaacg tgcagctgga

1801 acagggcacc gtgctgagcc tgctggaaga aaacggcacc atcaaaggcg tgcgctataa

1861 aaacaaagcg ggcgaagaac agaccgcgtt cgcggcgctg accatcgtgt gcgatggctg

1921 cttcagcaac ctgcgccgca gcctgtgcaa cccgcaggtg gaagtgccga gctgcttcgt

1981 gggcctggtg ctggaaaact gcaacctgcc gtatgcgaac catggccatg tggtgctggc

2041 ggatccgagc ccgatcctga tgtatccgat cagcagcacc gaagtgcgct gcctggtgga

2101 tgtgccgggc cagaaagtgc cgagcatcgc gaacggcgaa atgaaaaact atctgaaaac

2161 cgtggtggcg ccgcagatgc cgcatgaagt gtatgatagc ttcatcgcgg cggtggataa

2221 aggcaacatc aaaagcatgc cgaaccgcag catgccggcg agcccgtatc cgaccccggg

2281 cgcgctgctg atgggcgatg cgttcaacat gcgccatccg ctgaccggcg gcggcatgac

2341 cgtggcgctg gcggatatcg tggtgctgcg caacctgctg cgcccgctgc gcgatctgag

2401 cgatggcgcg agcctgtgca aatatctgga gagcttctat accctgcgca aaccggtggc

2461 ggcgaccatc aacaccctgg cgaacgcgct gtatcaggtg ttctgcagca gcgaaaacga

2521 agcgcgcaac gaaatgcgcg aagcgtgctt cgattatctg ggcctgggcg gcatgtgcac

2581 cagcggcccg gtgagcctgc tgagcggcct gaacccgcgc ccgctgaccc tggtgtgcca

2641 tttcttcgcg gtggcggtgt atggcgtgat ccgcctgctg atcccgttcc cgagcccgaa

2701 acgcatctgg ctgggcgcga aactgatcag cggcgcgagc ggcatcatct tcccgatcat

2761 caaagcggaa ggcgtgcgcc agatgttctt cccggcgacc gtgccggcgt attattataa

2821 agcgccgacc gtgggcgaaa ccaaatgcag ctaaacgcgt aaaagaggag aaatactaga

2881 tgtggcgcct gcgcatcggc gcggaagcgc gccaggatcc gcatctgttc accaccaaca

2941 acttcgcggg ccgccagatc tgggaattcg atgcgaacgg cggcagcccg gaagaactgg

3001 cggaagtgga agaagcgcgc ctgaacttcg cgaacaacaa aagccgcttc aaagcgagcc

3061 cggatctgtt ctggcgccgc cagttcctgc gcgaaaaaaa attcgaacag aaaatcccgc

3121 gcgtgcgcat cgaagatgcg gaaaaaatca cctatgaaga tgcgaaaacc gcgctgcgcc

3181 gcggcgtgct gtattatgcg gcgtgccagg cgaacgatgg ccattggccg agcgaagtga

3241 gcggcagcat gttcctggat gcgccgttcg tgatctgcct gtatatcacc ggccatctgg

3301 aaaaaatctt caccctggaa catgtgaaag aactgctgcg ctatatgtat aacacccaga

3361 acgaagatgg cggctggggc ctggatgtgg aaagccatag cgtgatgttc tgcaccgtgc

3421 tgaactatat ctgcctgcgc atcctgggcg tggaaccgga tcatgatggc cagaaaagcg

3481 cgtgcgcgcg cgcgcgcaaa tggatcctgg atcatggcgg cgcgacctat gcgccgatgg

3541 tggcgaaagc gtggctgagc gtgctgggcg tgtatgattg gagcggctgc aaaccgctgc

3601 cgccggaaat ctggatgctg ccgagcttca gcccgatcaa cggcggcacc ctgtggatct

3661 atatccgcga tctgctgatg ggcatgagct atctgtatgg caagaaattc gtggcgaccc

3721 cgaccgcgct gatcctgcag ctgcgcgaag aactgtatcc gcagccgtat agcaaaatca

3781 tctggagcaa agcgcgcaac cgctgcgcga aagaagatct gctgtatccg aagagcttcg

3841 gccaggatct gttctgggaa ggcgtgcaca tgctgagcga aaacatcatc aaccgctggc

3901 cgctgaacaa attcgtgcgc cagcgcgcgc tgcgcaccac catggaactg gtgcattatc

3961 atgatgaaac cacccattat atcaccggcg cgtgcgtggc gaaaccgttc cacatgctgg

4021 cgtgctgggt ggaagatccg gatggcgatt atttcaagaa acatctggcg cgcgtgccgg

4081 atttcatctg gatcgcggaa gatggcctga aattccagct gatgggcatg cagagctgga

4141 acgcggcgct gagcctgcag gtgatgctgg cggcgaacat ggatgatgaa atccgcagca

4201 ccctgatcaa aggctatgat ttcctgaaac agagccagat cagcgaaaac ccgcagggcg

4261 atcatctgaa aatgttccgc gatatcacca aaggcggctg gaccttccag gatcgcgaac

4321 agggcctgcc gatcagcgat ggcaccgcgg aaagcatcga atgctgcatc catttccatc

4381 gcatgccgag cgaattcatc ggcgaaaaaa tggatgtgga aaaactgtat gatgcggtga

4441 acttcctgat ctatctgcag agcgataacg gcggcatgcc ggtgtgggaa ccggcgccgg

4501 gcaagaaatg gctggaatgg ctgagcccgg tggaacatgt ggaaaacacc gtggtggaac

4561 aggaatatct ggaatgcacc ggcagcgtga tcgcgggcct ggtgtgcttc aagaaagaat

4621 tcccggatca tcgcccgaaa gaaatcgaaa aactgatcaa gaaaggcctg aaatatatcg

4681 aagatctgca gatgccggat ggcagctggt atggcaactg gggcgtgtgc ttcacctatg

4741 gcaccctgtt tgcggtgcgc ggcctggcgg cggcgggcaa aaccttcggc aacagcgaag

4801 cgatccgccg cgcggtgcag ttcatcctga acacccagaa cgcggaaggc ggctggggcg

4861 aaagcgcgct gagctgcccg aacaagaaat atatcccgag caaaggcaac gtgaccaacg

4921 tggtgaacac cggccaggcg atgatggtgc tgctgatcgg cggccagatg gaacgcgatc

4981 cgagcccggt gcatcgcgcg gcgaaagtgc tgatcaacag ccagctggat atcggcgatt

5041 tcccgcagca ggaacgccgc ggcatctata tgaacatgct gctgcattat ccgacctatc

5101 gcaacatgtt cagcctgtgg gcgctggcgc tgtataccaa cgcgctgcgc ctgctggtga

5161 gctaaaagct tgcggccgca ctcgagcacc accaccacca ccactgagat ccggctgcta

5221 acaaagcccg aaaggaagct gagttggctg ctgccaccgc tgagcaataa ctagcataac

5281 cccttggggc ctctaaacgg gtcttgaggg gttttttg

//

**pRhofHi-2- SQS1-SQE1-THAS1**

LOCUS SQS1-SQE1-THAS1 5309 bp DNA linear SYN 25-OCT-2017

KEYWORDS pRhofHi-2-SQS-SQE-THAS1

FEATURES Location/Qualifiers

CDS 4..1236

/gene="SQS1"

/product="A. thaliana squalene synthase SQS1"

/translation="MGSLGTMLRYPDDIYPLLKMKRAIEKAEKQIPPEPHWGFCYSML

HKVSRSFSLVIQQLNTELRNAVCVFYLVLRALDTVEDDTSIPTDEKVPILIAFHRHIY

DTDWHYSCGTKEYKILMDQFHHVSAAFLELEKGYQEAIEEITRRMGAGMAKFICQEVE

TVDDYDEYCHYVAGLVGLGLSKLFLAAGSEVLTPDWEAISNSMGLFLQKTNIIRDYLE

DINEIPKSRMFWPREIWGKYADKLEDLKYEENTNKSVQCLNEMVTNALMHIEDCLKYM

VSLRDPSIFRFCAIPQIMAIGTLALCYNNEQVFRGVVKLRRGLTAKVIDRTKTMADVY

GAFYDFSCMLKTKVDKNDPNASKTLNRLEAVQKLCRDAGVLQNRKSYVNDKGQPNSVF

IIMVVILLAIVFAYLRAN"

regulatory 1245..1250

/regulatory_class=ribosome_binding_site

/gene="pET RBS"

/product="RBS of pET vector series"

CDS 1259..2854

/gene="SQE1"

/product="A. thaliana squalene epoxidase SQE1"

/translation="MESQLWNWILPLLISSLLISFVAFYGFFVKPKRNGLRHDRKTVS

TVTSDVGSVNITGDTVADVIVVGAGVAGSALAYTLGKDKRRVHVIERDLSEPDRIVGE

LLQPGGYLKLLELGIEDCVEEIDAQRVYGYALFKNGKRIRLAYPLEKFHEDVSGRSFH

NGRFIQRMREKAASLPNVQLEQGTVLSLLEENGTIKGVRYKNKAGEEQTAFAALTIVC

DGCFSNLRRSLCNPQVEVPSCFVGLVLENCNLPYANHGHVVLADPSPILMYPISSTEV

RCLVDVPGQKVPSIANGEMKNYLKTVVAPQMPHEVYDSFIAAVDKGNIKSMPNRSMPA

SPYPTPGALLMGDAFNMRHPLTGGGMTVALADIVVLRNLLRPLRDLSDGASLCKYLES

FYTLRKPVAATINTLANALYQVFCSSENEARNEMREACFDYLGLGGMCTSGPVSLLSG

LNPRPLTLVCHFFAVAVYGVIRLLIPFPSPKRIWLGAKLISGASGIIFPIIKAEGVRQ

MFFPATVPAYYYKAPTVGETKCS"

regulatory 2862..2873

/regulatory_class=ribosome_binding_site

/gene="BBa_0034 RBS"

/product="Biobrick RBS BBa_0034"

CDS 2880..5156

/gene="THAS1"

/product="A. thaliana thalianol synthase THAS1"

/translation="MWRLRTGPKAGEDTHLFTTNNYAGRQIWEFDANAGSPQEIAEVE

DARHKFSDNTSRFKTTADLLWRMQFLREKKFEQKIPRVIIEDARKIKYEDAKTALKRG

LLYFTALQADDGHWPAENSGPNFYTPPFLICLYITGHLEKIFTPEHVKELLRHIYNMQ

NEDGGWGLHVESHSVMFCTVINYVCLRIVGEEVGHDDQRNGCAKAHKWIMDHGGATYT

PLIGKALLSVLGVYDWSGCNPIPPEFWLLPSSFPVNGGTLWIYLRDTFMGLSYLYGKK

FVAPPTPLILQLREELYPEPYAKINWTQTRNRCGKEDLYYPRSFLQDLFWKSVHMFSE

SILDRWPLNKLIRQRALQSTMALIHYHDESTRYITGGCLPKAFHMLACWIEDPKSDYF

KKHLARVREYIWIGEDGLKIQSFGSQLWDTALSLHALLDGIDDHDVDDEIKTTLVKGY

DYLKKSQITENPRGDHFKMFRHKTKGGWTFSDQDQGWPVSDCTAESLECCLFFESMPS

ELIGKKMDVEKLYDAVDYLLYLQSDNGGIAAWQPVEGKAWLELLNIMIFRYVECTGSA

IAALTQFNKQFPGYKNVEVKRFITKAAKYIEDMQTVDGSWYGNWGVCFIYGTFFAVRG

LVAAGKTYSNCEAIRKAVRFLLDTQNPEGGWGESFLSCPSKKYTPLKGNSTNVVQTAQ

ALMVLIMGDQMERDPLPVHRAAQVLINSQLDNGDFPQQEIMGTFMRTVMLHFPTYRNT

FSLWALTHYTHALRRLLP"

regulatory 5262..5309

/regulatory_class=terminator

/gene="T7 terminator"

/product="bacteriophage T7 transcription terminator"

ORIGIN

1 catatgggca gcctgggcac catgctgcgc tatccggatg atatctatcc gctgctgaaa

61 atgaaacgcg cgatcgaaaa agcggaaaaa cagatcccgc cggaaccgca ttggggcttc

121 tgctatagca tgctgcataa agtgagccgc agcttcagcc tggtgatcca gcagctgaac

181 accgaactgc gcaacgcggt gtgcgtgttc tatctggtgc tgcgcgcgct ggataccgtg

241 gaagatgata ccagcatccc gaccgatgaa aaagtgccga tcctgatcgc gttccatcgc

301 catatctatg ataccgattg gcattatagc tgcggcacca aagaatataa aatcctgatg

361 gatcagttcc atcatgtgag cgcggcgttc ctggaactgg aaaaaggcta tcaggaagcg

421 atcgaagaaa tcacccgccg catgggcgcg ggcatggcga aattcatctg ccaggaagtg

481 gaaaccgtgg atgattatga tgaatattgc cattatgtgg cgggcctggt gggcctgggc

541 ctgagcaaac tgttcctggc ggcgggcagc gaagtgctga ccccggattg ggaagcgatc

601 agcaacagca tgggcctgtt cctgcagaaa accaacatca tccgcgatta tctggaagat

661 atcaacgaaa tcccgaaaag ccgcatgttc tggccgcgcg aaatctgggg caaatatgcg

721 gataaactgg aagatctgaa atatgaagaa aacaccaaca aaagcgtgca gtgcctgaac

781 gaaatggtga ccaacgcgct gatgcatatc gaagattgcc tgaaatatat ggtgagcctg

841 cgcgatccga gcatcttccg cttctgcgcg atcccgcaga tcatggcgat cggcaccctg

901 gcgctgtgct ataacaacga acaggtgttc cgcggcgtgg tgaaactgcg ccgcggcctg

961 accgcgaaag tgatcgatcg caccaaaacc atggcggatg tgtatggcgc gttctatgat

1021 ttcagctgca tgctgaaaac caaagtggat aaaaacgatc cgaacgcgag caaaaccctg

1081 aaccgcctgg aagcggtgca gaaactgtgc cgcgatgcgg gcgtgctgca gaaccgcaaa

1141 agctatgtga acgataaagg ccagccgaac agcgtgttca tcatcatggt ggtgatcctg

1201 ctggcgatcg tgttcgcgta tctgcgcgcg aactaactcg agataaggag atatacacat

1261 ggaaagccag ctgtggaact ggatcctgcc gctgctgatc agcagcctgc tgatcagctt

1321 cgtggcgttc tatggcttct tcgtgaaacc gaaacgcaac ggcctgcgcc atgatcgcaa

1381 aaccgtgagc accgtgacca gcgatgtggg cagcgtgaac atcaccggcg ataccgtggc

1441 ggatgtgatc gtggtgggcg cgggcgtggc gggcagcgcg ctggcgtata ccctgggcaa

1501 agataaacgc cgcgtgcatg tgatcgaacg cgatctgagc gaaccggatc gcatcgtggg

1561 cgaactgctg cagccgggcg gctatctgaa actgctggaa ctgggcatcg aagattgcgt

1621 ggaagaaatc gatgcgcagc gcgtgtatgg ctatgcgctg ttcaaaaacg gcaaacgcat

1681 ccgcctggcg tatccgctgg aaaaattcca tgaagatgtg agcggccgca gcttccataa

1741 cggccgcttc atccagcgca tgcgcgaaaa agcggcgagc ctgccgaacg tgcagctgga

1801 acagggcacc gtgctgagcc tgctggaaga aaacggcacc atcaaaggcg tgcgctataa

1861 aaacaaagcg ggcgaagaac agaccgcgtt cgcggcgctg accatcgtgt gcgatggctg

1921 cttcagcaac ctgcgccgca gcctgtgcaa cccgcaggtg gaagtgccga gctgcttcgt

1981 gggcctggtg ctggaaaact gcaacctgcc gtatgcgaac catggccatg tggtgctggc

2041 ggatccgagc ccgatcctga tgtatccgat cagcagcacc gaagtgcgct gcctggtgga

2101 tgtgccgggc cagaaagtgc cgagcatcgc gaacggcgaa atgaaaaact atctgaaaac

2161 cgtggtggcg ccgcagatgc cgcatgaagt gtatgatagc ttcatcgcgg cggtggataa

2221 aggcaacatc aaaagcatgc cgaaccgcag catgccggcg agcccgtatc cgaccccggg

2281 cgcgctgctg atgggcgatg cgttcaacat gcgccatccg ctgaccggcg gcggcatgac

2341 cgtggcgctg gcggatatcg tggtgctgcg caacctgctg cgcccgctgc gcgatctgag

2401 cgatggcgcg agcctgtgca aatatctgga gagcttctat accctgcgca aaccggtggc

2461 ggcgaccatc aacaccctgg cgaacgcgct gtatcaggtg ttctgcagca gcgaaaacga

2521 agcgcgcaac gaaatgcgcg aagcgtgctt cgattatctg ggcctgggcg gcatgtgcac

2581 cagcggcccg gtgagcctgc tgagcggcct gaacccgcgc ccgctgaccc tggtgtgcca

2641 tttcttcgcg gtggcggtgt atggcgtgat ccgcctgctg atcccgttcc cgagcccgaa

2701 acgcatctgg ctgggcgcga aactgatcag cggcgcgagc ggcatcatct tcccgatcat

2761 caaagcggaa ggcgtgcgcc agatgttctt cccggcgacc gtgccggcgt attattataa

2821 agcgccgacc gtgggcgaaa ccaaatgcag ctaaacgcgt aaaagaggag aaatactaga

2881 tgtggcgcct gcgcaccggc ccgaaagcgg gcgaagatac ccatctgttc accaccaaca

2941 actatgcggg ccgccagatc tgggaattcg atgcgaacgc gggcagcccg caggaaatcg

3001 cggaagtgga agatgcgcgc cataaattca gcgataacac cagccgcttc aaaaccaccg

3061 cggatctgct gtggcgcatg cagttcctgc gcgaaaaaaa attcgaacag aaaatcccgc

3121 gcgtgatcat cgaagatgcg cgcaaaatca aatatgaaga tgcgaaaacc gcgctgaaac

3181 gcggcctgct gtatttcacc gcgctgcagg cggatgatgg ccattggccg gcggaaaaca

3241 gcggcccgaa cttctatacc ccgccgttcc tgatctgcct gtatatcacc ggccatctgg

3301 aaaaaatctt caccccggaa catgtgaaag aactgctgcg ccatatctat aacatgcaga

3361 acgaagatgg cggctggggc ctgcatgtgg aaagccatag cgtgatgttc tgcaccgtga

3421 tcaactatgt gtgcctgcgc atcgtgggcg aagaagtggg ccatgatgat cagcgcaacg

3481 gctgcgcgaa agcgcataaa tggatcatgg atcatggcgg cgcgacctat accccgctga

3541 tcggcaaagc gctgctgagc gtgctgggcg tgtatgattg gagcggctgc aacccgatcc

3601 cgccggaatt ctggctgctg ccgagcagct tcccggtgaa cggcggcacc ctgtggatct

3661 atctgcgcga taccttcatg ggcctgagct atctgtatgg caaaaaattc gtggcgccgc

3721 cgaccccgct gatcctgcag ctgcgcgaag aactgtatcc ggaaccgtat gcgaaaatca

3781 actggaccca gacccgcaac cgctgcggca aagaagatct gtattatccg cgcagcttcc

3841 tgcaggatct gttctggaaa agcgtgcaca tgttcagcga aagcatcctg gatcgctggc

3901 cgctgaacaa actgatccgc cagcgcgcgc tgcagagcac catggcgctg atccattatc

3961 atgatgaaag cacccgctat atcaccggcg gctgcctgcc gaaagcgttc cacatgctgg

4021 cgtgctggat cgaagatccg aaaagcgatt atttcaaaaa acatctggcg cgcgtgcgcg

4081 aatatatctg gatcggcgaa gatggcctga aaatccagag cttcggcagc cagctgtggg

4141 ataccgcgct gagcctgcat gcgctgctgg atggcatcga tgatcatgat gtggatgatg

4201 aaatcaaaac caccctggtg aaaggctatg attatctgaa aaaaagccag atcaccgaaa

4261 acccgcgcgg cgatcatttc aaaatgttcc gccataaaac caaaggcggc tggaccttca

4321 gcgatcagga tcagggctgg ccggtgagcg attgcaccgc ggaaagcctg gaatgctgcc

4381 tgttcttcga aagcatgccg agcgaactga tcggcaaaaa aatggatgtg gaaaaactgt

4441 atgatgcggt ggattatctg ctgtatctgc agagcgataa cggcggcatc gcggcgtggc

4501 agccggtgga aggcaaagcg tggctggaac tgctgaacat catgatcttc cgctatgtgg

4561 aatgcaccgg cagcgcgatc gcggcgctga cccagttcaa caaacagttc ccgggctata

4621 aaaacgtgga agtgaaacgc ttcatcacca aagcggcgaa atatatcgaa gatatgcaga

4681 ccgtggatgg cagctggtat ggcaactggg gcgtgtgctt catctatggc accttctttg

4741 cggtgcgcgg cctggtggcg gcgggcaaaa cctatagcaa ctgcgaagcg atccgcaaag

4801 cggtgcgctt cctgctggat acccagaacc cggaaggcgg ctggggcgag agcttcctga

4861 gctgcccgag caaaaaatat accccgctga aaggcaacag caccaacgtg gtgcagaccg

4921 cgcaggcgct gatggtgctg atcatgggcg atcagatgga acgcgatccg ctgccggtgc

4981 atcgcgcggc gcaggtgctg atcaacagcc agctggataa cggcgatttc ccgcagcagg

5041 aaatcatggg caccttcatg cgcaccgtga tgctgcattt cccgacctat cgcaacacct

5101 tcagcctgtg ggcgctgacc cattataccc atgcgctgcg ccgcctgctg ccgtaaaagc

5161 ttgcggccgc actcgagcac caccaccacc accactgaga tccggctgct aacaaagccc

5221 gaaaggaagc tgagttggct gctgccaccg ctgagcaata actagcataa ccccttgggg

5281 cctctaaacg ggtcttgagg ggttttttg

//
