# Supplementary material for: The photosynthetic bacteria Rhodobacter capsulatus and Synechocystis sp. PCC 6803 as new hosts for cyclic plant triterpene biosynthesis
Source: PLoS One. 2017 Dec 27;12(12):e0189816. doi: 10.1371/journal.pone.0189816 (PMC5744966; doi:10.1371/journal.pone.0189816)
Supplement: S3 Table — (PDF) [file pone.0189816.s003.pdf]

The photosynthetic bacteria *Rhodobacter capsulatus* and *Synechocystis* sp. PCC 6803 as new hosts for cyclic plant triterpene biosynthesis

Anita Loeschcke, Dennis Dienst Dienst, Vera Wewer, Jennifer Hage-Hülsmann, Maximilian Dietsch, Sarah Kranz-Finger, Vanessa Hüren, Sabine Metzger, Vlada B. Urlacher, Tamara Gigolashvili, Stanislav Kopriva, Ilka M. Axmann, Thomas Drepper, Karl-Erich Jaeger

S3 Table. Sum formulas and calculated m/z of selected triterpenes. Proton adducts $[M+H]^+$ and sodium adducts $[M+Na]^+$ were detected for all triterpenes during LC-MS analysis. Loss of H_2O due to in-source decay during electrospray ionization was observed for all triterpenes except squalene, most notably for lupeol. M/z corresponding to $[M+H-H_2O]^+$ and $[M+H-(H_2O)_2]^+$ were observed for hydroxymarnerol, and the product of LUP1, the putative 3,20-dihydroxylupane.

triterpene	formula	$[M+H]^+$ (m/z)	$[M+Na]^+$ (m/z)	$[M+H-H_2O]^+$ (m/z)	$[M+H-(H_2O)_2]^+$ (m/z)
squalene	$C_{30}H_{50}$	411.399	433.380	-	-
2,3-oxidosqualene	$C_{30}H_{50}O$	427.393	449.375	409.383	-
cycloartenol	$C_{30}H_{50}O$	427.393	449.375	409.383	-
lupeol	$C_{30}H_{50}O$	427.393	449.375	409.383	-
thalianol	$C_{30}H_{50}O$	427.393	449.375	409.383	-
marneral	$C_{30}H_{50}O$	427.393	449.375	409.383	-
marnerol	$C_{30}H_{52}O$	429.409	451.391	411.399	-
hydroxymarnerol	$C_{30}H_{52}O_2$	445.404	467.386	427.393	409.383
lupanediol/3,20-dihydroxylupane	$C_{30}H_{52}O_2$	445.404	467.386	427.393	409.383
