# Supplementary material for: The photosynthetic bacteria Rhodobacter capsulatus and Synechocystis sp. PCC 6803 as new hosts for cyclic plant triterpene biosynthesis
Source: PLoS One. 2017 Dec 27;12(12):e0189816. doi: 10.1371/journal.pone.0189816 (PMC5744966; doi:10.1371/journal.pone.0189816)
Supplement: S1 Fig — Document containing plasmid maps of vectors for expression in R. capsulatus (pRhon5Hi-2, pRhon5Hi-2-SQS1, pRhon5Hi-2-SQS1-SQE1, and pRhon5Hi-2-LUP1-SQS1-SQE1) and of vectors for expression in Synechocystis (pVZ-spec, pVZ-PcoaT-SQE1, and pVZ-PcoaT-SQE1-LUP1). (DOCX) [file pone.0189816.s004.docx]

**The photosynthetic bacteria *Rhodobacter capsulatus* and *Synechocystis* sp. PCC 6803 as new hosts for cyclic plant triterpene biosynthesis**

Anita Loeschcke, Dennis Dienst Dienst, Vera Wewer, Jennifer Hage-Hülsmann, Maximilian Dietsch, Sarah Kranz-Finger, Vanessa Hüren, Sabine Metzger, Vlada B. Urlacher, Tamara Gigolashvili, Stanislav Kopriva, Ilka M. Axmann, Thomas Drepper, Karl-Erich Jaeger

**S1 Fig. Plasmid maps.**

**content**

**page**

**Plasmid maps**

S1 Fig.A-D pRhon5Hi-2-based vectors for expression in *R. capsulatus* 2-5

S1 Fig.E-G pVZ-spec-based vectors for expression in *Synechocystis* 6-8

All plant triterpene biosynthesis genes, derived from *Arabidopsis thaliana*, were obtained as synthetic genes with adapted codon usage for expression in bacterial hosts.

Sequences of inserted expression cassettes in shown plasmids are provided in the separate file of **S2 Table**.


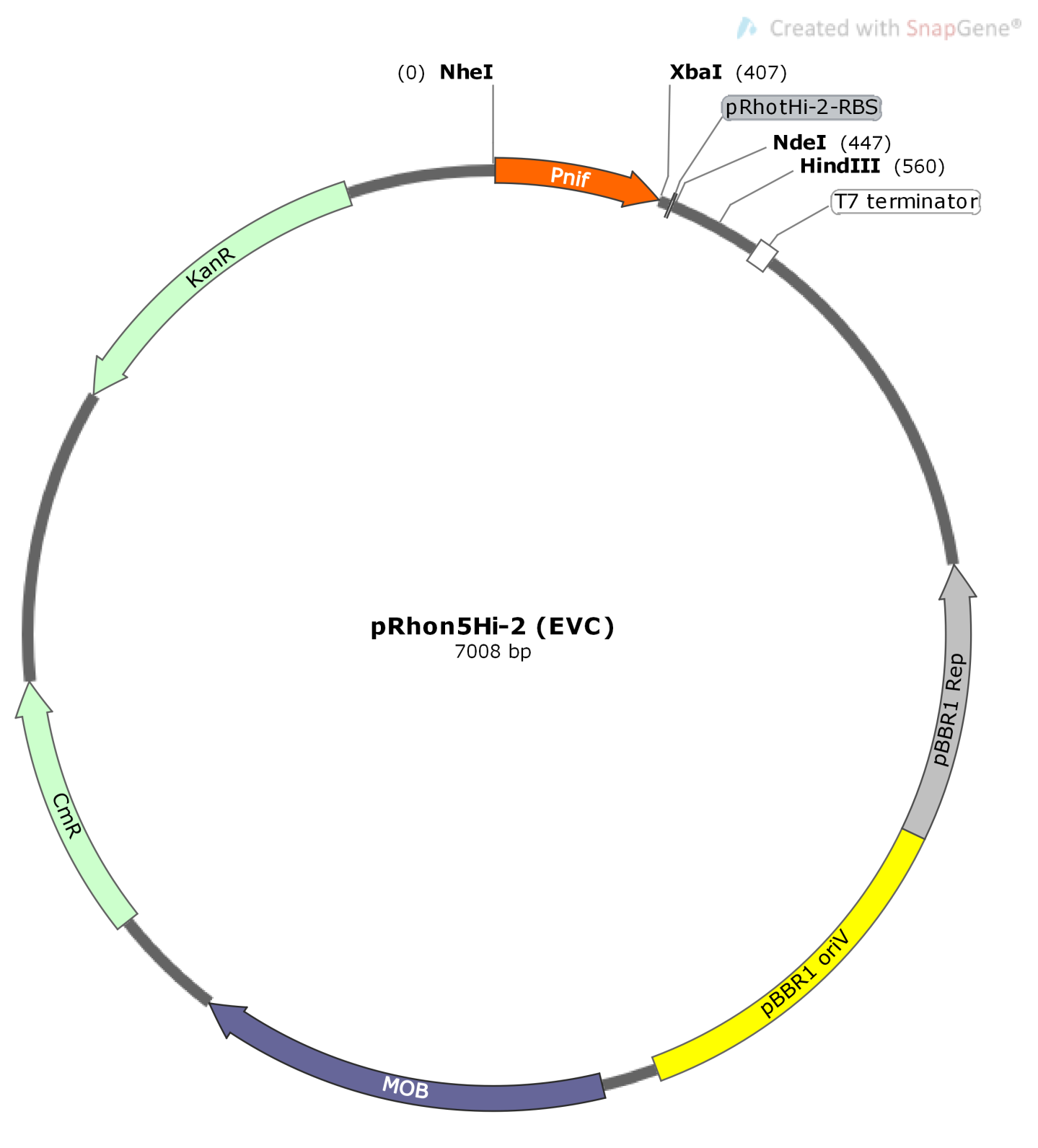


**S1 Fig.A**: Map of vector pRhon5Hi-2 used as plasmid backbone for expression of triterpene biosynthesis cassettes in *R. capsulatus*. ’EVC‘ indicates that this plasmid was used as empty vector control in the experiments. Restriction sites used for cloning are indicated. CmR, KanR: resistance cassettes for chloramphenicol and kanamycin, respectively. P*nif*, native promoter of the nitrogenase *nifHDK* operon *R. capsulatus* B10S*;* pRhotHi-2 RBS: 5‘UTR including ribosomal binding site from vector pRhotHi-2; REP: gene relevant for replication; MOB: mobilization cassette.


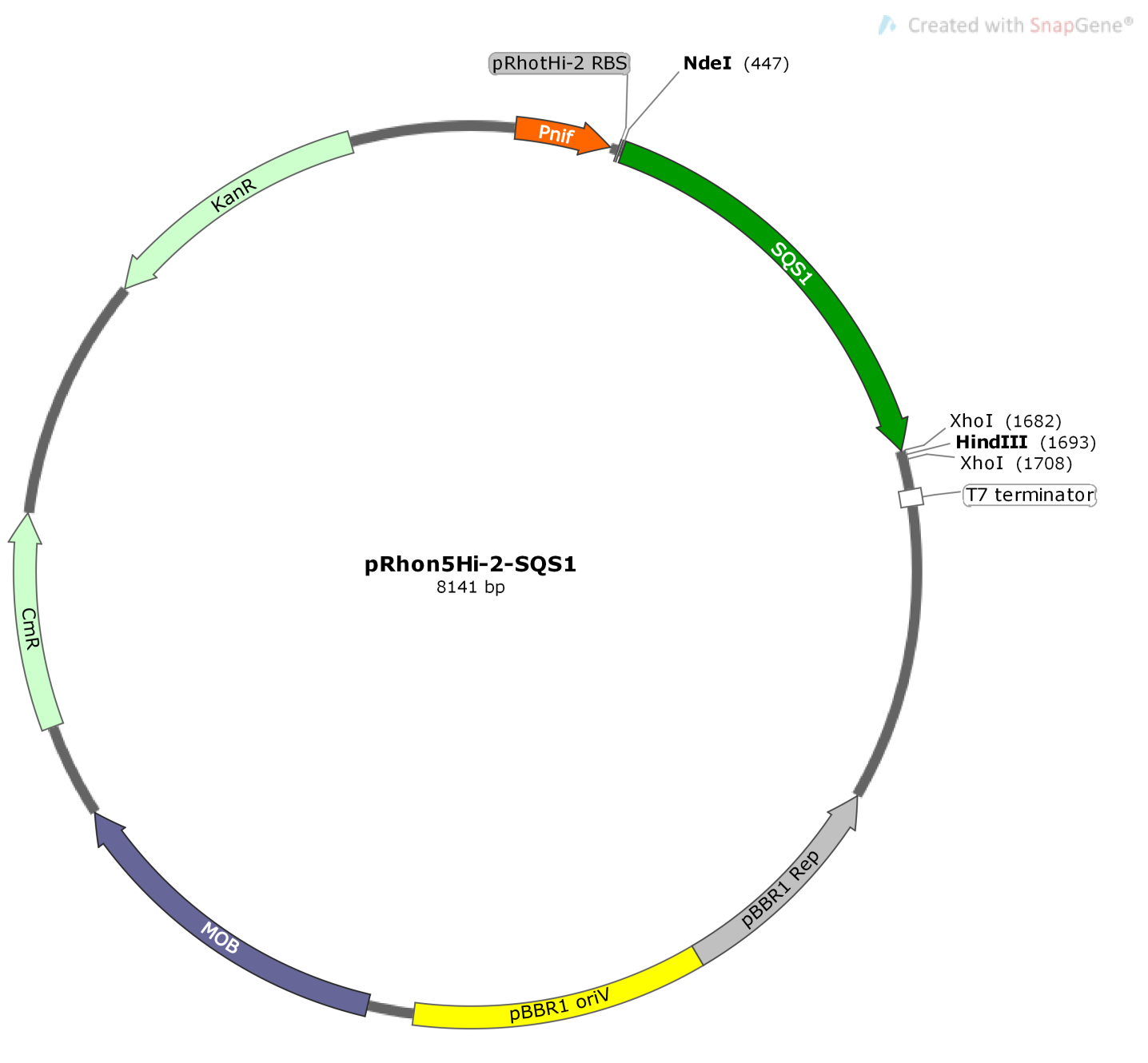


**S1 Fig.B**: Map of vector pRhon5Hi-2-SQS1 used for heterologous biosynthesis of squalene in *R. capsulatus.* The codon optimized CDS (coding sequence) for SQS1 from *A. thaliana* was inserted into vector pRhon5Hi-2 (S1 Fig.A) by *Nde*I/*Hind*III cloning. Gene expression is mediated by promoter P*nif* and the RBS from expression plasmid pRhotHi-2. Transcription terminates at a phage T7 terminator.


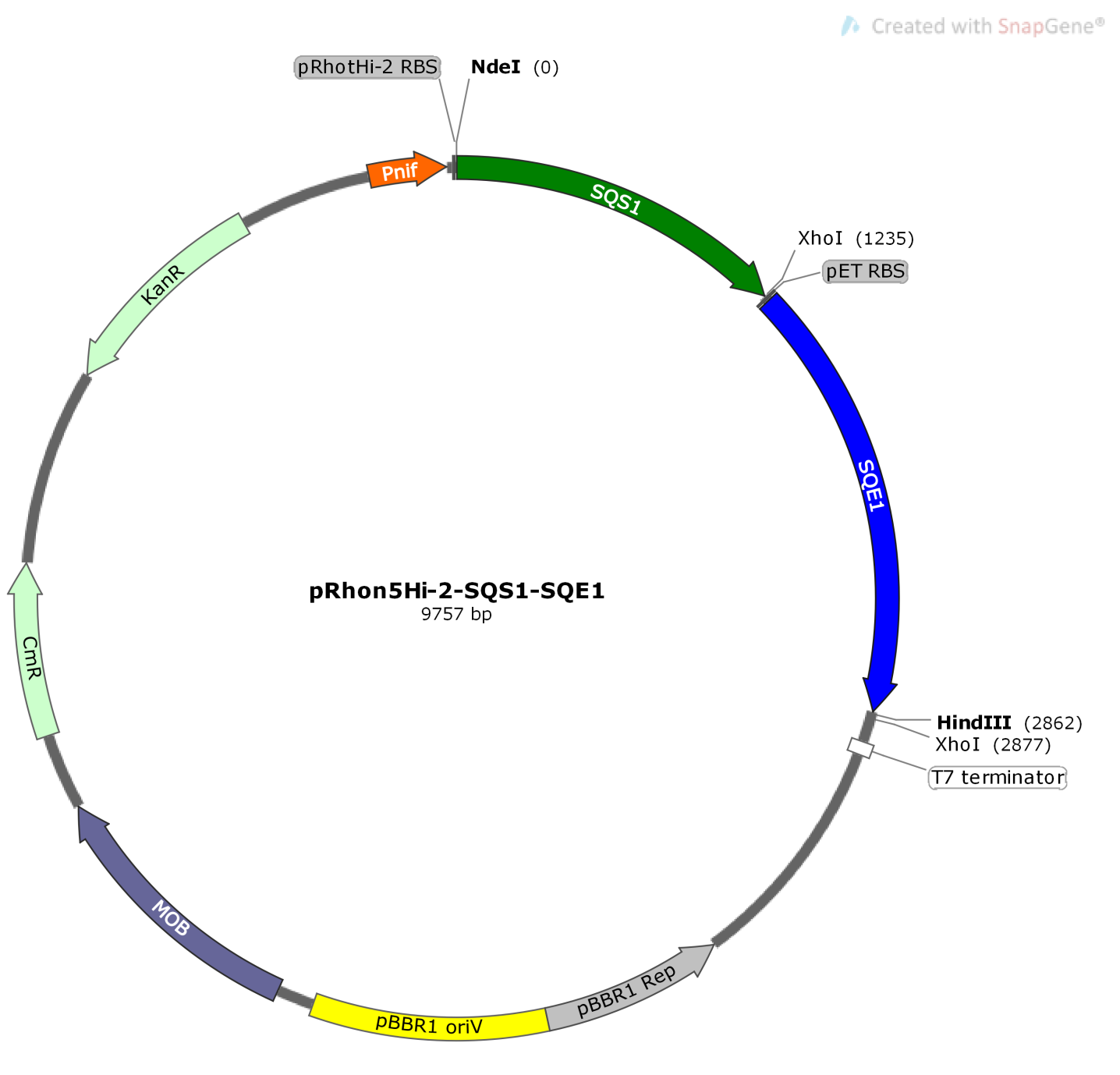


**S1 Fig.C**: Map of vector pRhon5Hi-2-SQS1-SQE1 used for heterologous biosynthesis of 2,3‑oxidosqualene in *R. capsulatus.* The codon usage adapted CDS for SQS1 and SQE1 from *A. thaliana* were inserted into vector pRhon5Hi-2 (S1 Fig.A) by *Nde*I/*Hind*III cloning. Gene expression is mediated by promoter P*nif* and the RBS sequences from expression plasmid pRhotHi-2 (*SQS1*), and pET (*SQE1*).Transcription terminates at a phage T7 terminator.


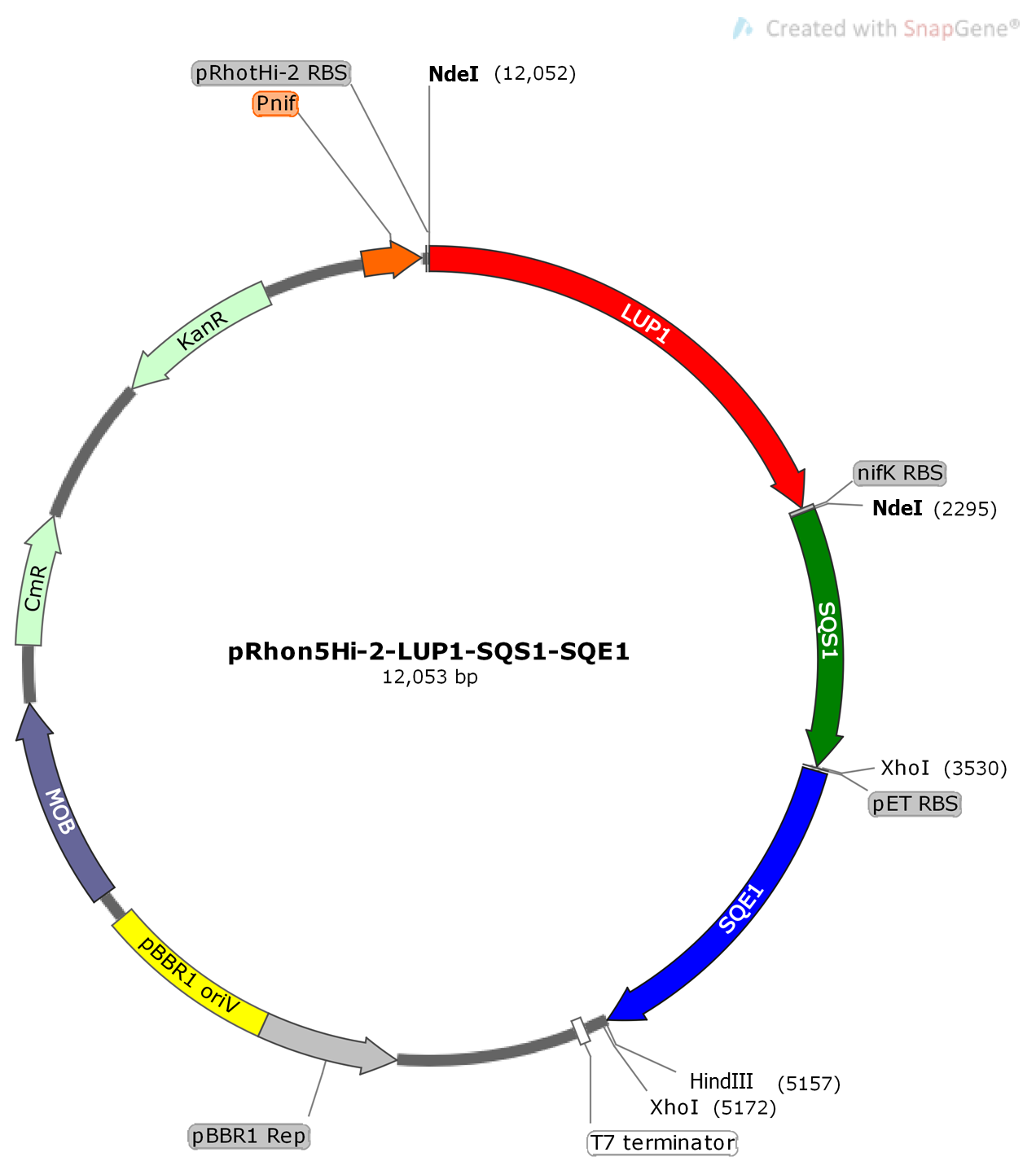


**S1 Fig.D**: Map of vector pRhon5Hi-2-LUP1-SQS1-SQE1 used for heterologous biosynthesis of lupeol/lupX in *R. capsulatus.* A fragment harboring the codon usage adapted *LUP1* CDS from *A. thaliana* followed by the 5‘UTR of the native *nifK* gene at the 3’ end was inserted into pRhon5Hi-2-*SQS1*-*SQE1* (S1 Fig.C) by *Nde*I cloning. Gene expression is mediated by promoter P*nif* and the RBS sequences from vector pRhotHi-2 (*LUP1*), the *nifK* gene (*SQS1*), and pET vectors (*SQE1*). This map is representative for all four pRhon5Hi-2-based OSC expression vectors used in this study.


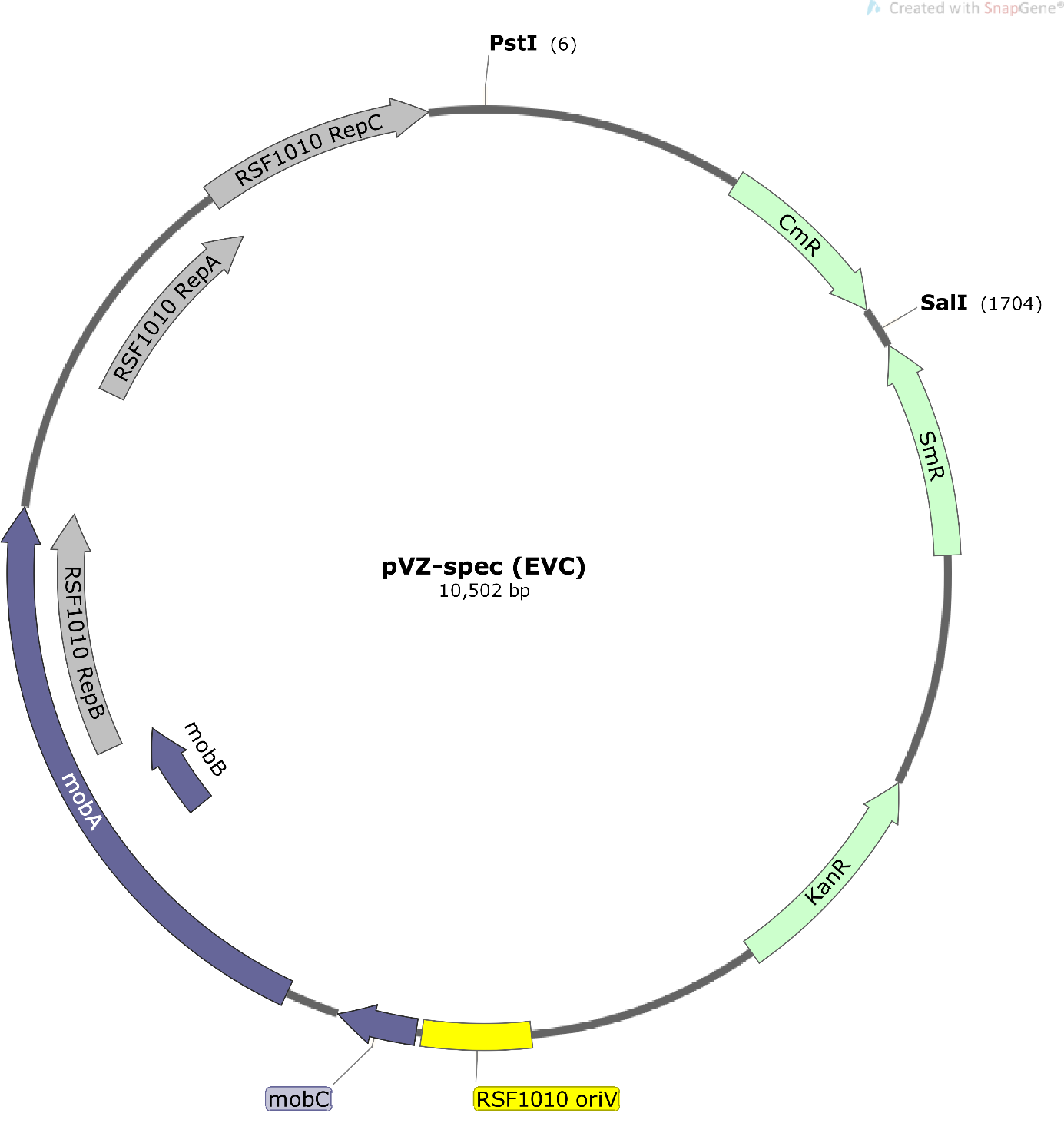


**S1 Fig.E**: Map of vector pVZ-spec used as plasmid backbone for expression of triterpene biosynthesis cassettes in *Synechocystis*. ‘EVC’ indicates that this plasmid was used as empty vector control in the experiments. Restriction sites used for cloning are indicated. CmR, KanR and SmR: resistance cassettes for chloramphenicol, kanamycin and spectinomycin/streptomycin, respectively. Rep, genes encoding replication proteins; mobABC: genes encoding mobilization proteins required for conjugational transfer.


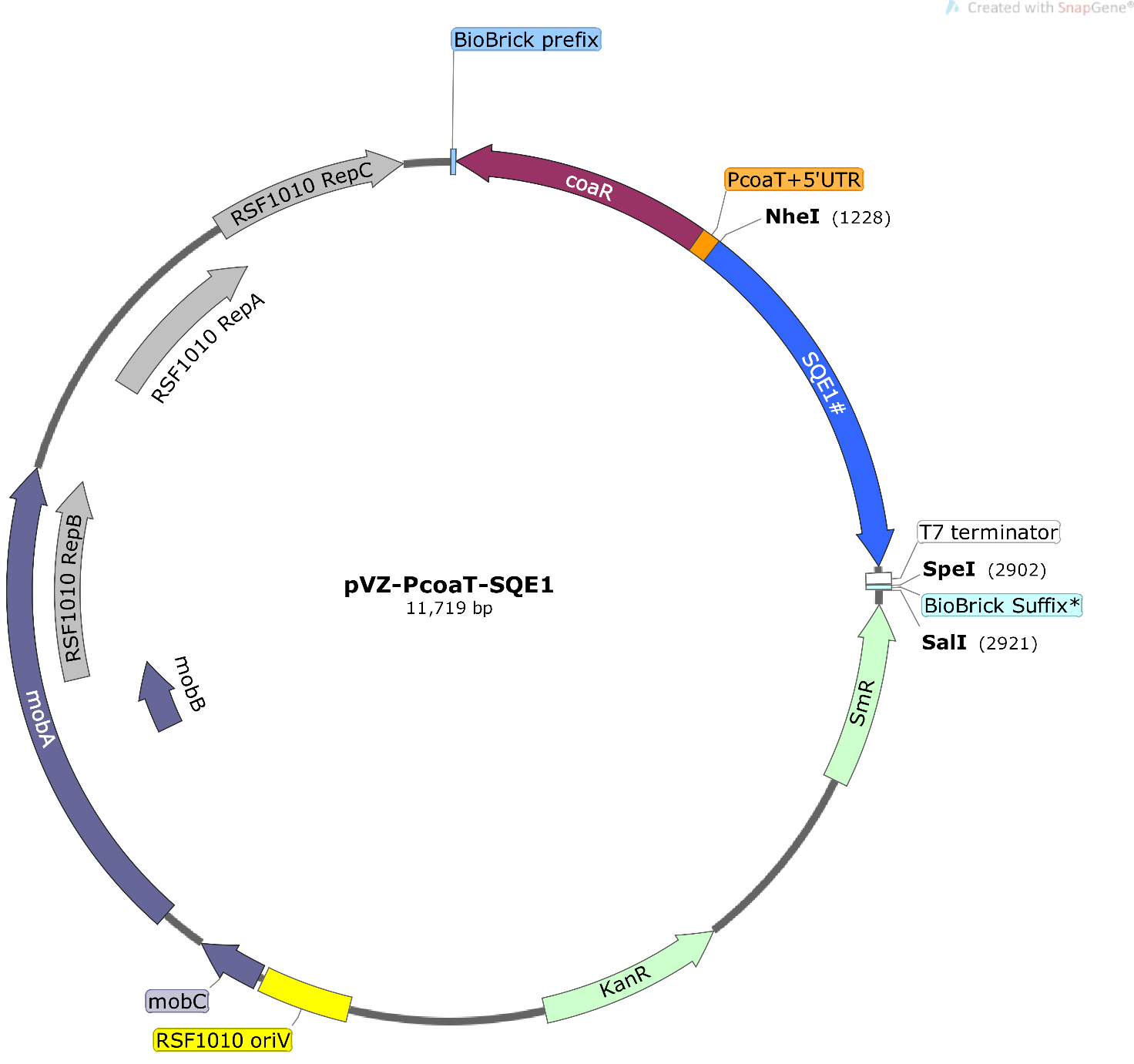


**S1 Fig.F**: Map of vector pVZ-PcoaT-SQE1 used for heterologous biosynthesis of 2,3-oxidosqualene in *Synechocystis.* Insertion of the construct ‚BioBrick - Prefix - coaR - PcoaT+5‘UTR - SQE1# - T7 terminator - BioBrickSuffix*‘ into pVZ‑spec (S1 Fig.E) was conducted via the plasmid´s restriction sites *Pst*I and *Sal*I. The original *Pst*I site was destroyed by ligation with the construct´s compatible *Psi*I site. Gene expression in *Synechocystis* is mediated by the native cobalt-inducible promoter P*coaT* and the respective RBS from gene *coaT*. Transcription terminates at a phage T7 terminator. # for pVZ-PcoaT-SQE1, an *Nhe*I site was introduced into the 2^nd^ and 3^rd^ codon of *SQE1*, *the BioBrick suffix in plasmid pVZ-PcoaT-SQE1 lacks the 5’T of the standard suffix.


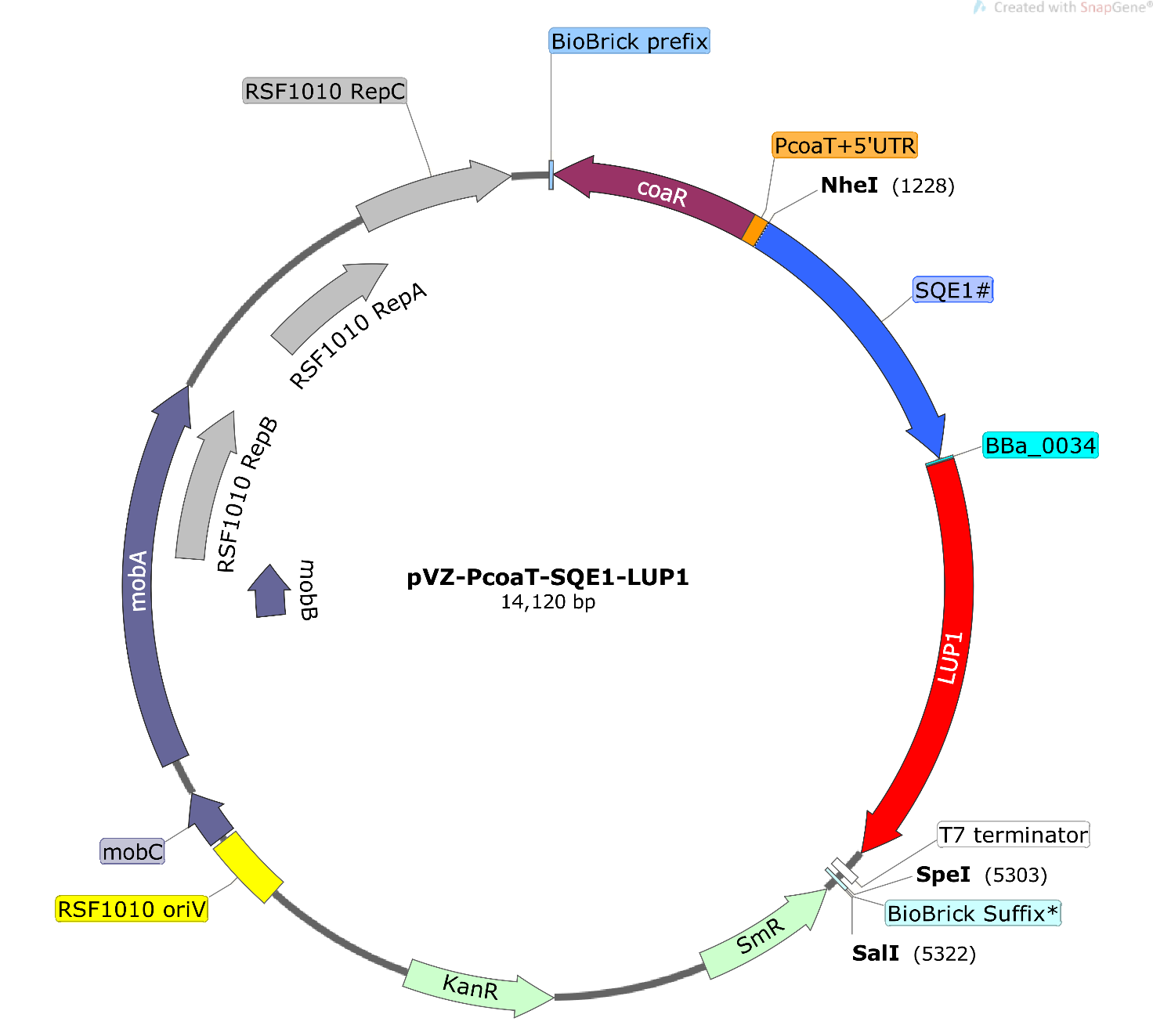


**S1 Fig.G**: Map of vector pVZ-PcoaT-SQE1-LUP1 used for heterologous biosynthesis of lupeol/lupX in *Synechocystis.* Insertion of the construct ‚BioBrick - Prefix - coaR - PcoaT+5‘UTR - SQE1* - BBa0034 - LUP1 - T7 terminator - BioBrick Suffix‘ into pVZ-spec (S1 Fig.E) was conducted via the plasmid´s restriction sites *Pst*I and *Sal*I. The *Pst*I site was destroyed by ligation with the construct´s compatible *Psi*I site. Gene expression in *Synechocystis* was mediated by the native cobalt-inducible promoter P*coaT* and – for *SQE1* – the respective RBS from gene *coaT*. Translation of *LUP1* is mediated by RBS B0034 from BioBrick part BBa_0034.This map is representative for all four pVZ-P*coaT*-based OSC expression vectors used in this study.
