# Supplementary material for: The photosynthetic bacteria Rhodobacter capsulatus and Synechocystis sp. PCC 6803 as new hosts for cyclic plant triterpene biosynthesis
Source: PLoS One. 2017 Dec 27;12(12):e0189816. doi: 10.1371/journal.pone.0189816 (PMC5744966; doi:10.1371/journal.pone.0189816)
Supplement: S2 Fig — Document containing MS spectra of all analyzed triterpenoids as detected in bacteria; MS/MS spectra of triterpenoids squalene, 2,3-oxidosqualene, cycloartenol, lupeol and lupX as detected in bacteria; LC-MS chromatrograms of extracts from THAS1-expressing bacteria; MS/MS spectra of marnerol/hydroxymarnerol as detected in Synechocystis. (DOCX) [file pone.0189816.s005.docx]

**The photosynthetic bacteria *Rhodobacter capsulatus* and *Synechocystis* sp. PCC 6803 as new hosts for cyclic plant triterpene biosynthesis**

Anita Loeschcke, Dennis Dienst Dienst, Vera Wewer, Jennifer Hage-Hülsmann, Maximilian Dietsch, Sarah Kranz-Finger, Vanessa Hüren, Sabine Metzger, Vlada B. Urlacher, Tamara Gigolashvili, Stanislav Kopriva, Ilka M. Axmann, Thomas Drepper, Karl-Erich Jaeger

**S2 Fig. LC-MS data.**

**content**

**page**

**LC-MS data**

S2 Fig.A MS spectra of all analyzed triterpenoids

[(a) *R. capsulatus*, (b) *Synechocystis*] 2-3

S2 Fig.B MS/MS spectra of triterpenoids

squalene, 2,3-oxidosqualene and cycloartenol [(a) *R. capsulatus*, (b) *Synechocystis*] 4-5

S2 Fig.C MS/MS spectra of triterpenoids lupeol and lupX

[(a) *R. capsulatus*, (b) *Synechocystis*] 6-7

S2 Fig.D LC-MS chromatrograms of extracts from THAS1-expressing bacteria

[(a) *R. capsulatus*, (b) *Synechocystis*] 8

S2 Fig.E MS/MS spectra of marnerol/hydroxymarnerol

[*Synechocystis*] 9

(a)

**
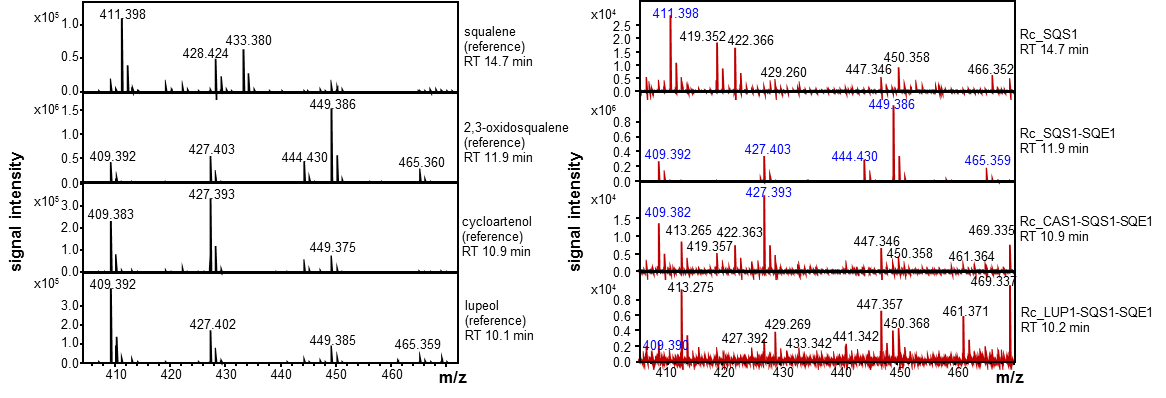
**

**S2 Fig.A (a): MS spectra of triterpenoids as detected by LC-MS in *R. capsulatus* expression strains.** Signals of expected masses obtained with reference compounds (left) are highlighted blue in extracts from bacteria (right). See **S3 Table** for triterpenoid sum formulas and calculated m/z. Rc, *R. capsulatus*.

Figure part (b) for *Synechocystis* is shown on next page.

(b)


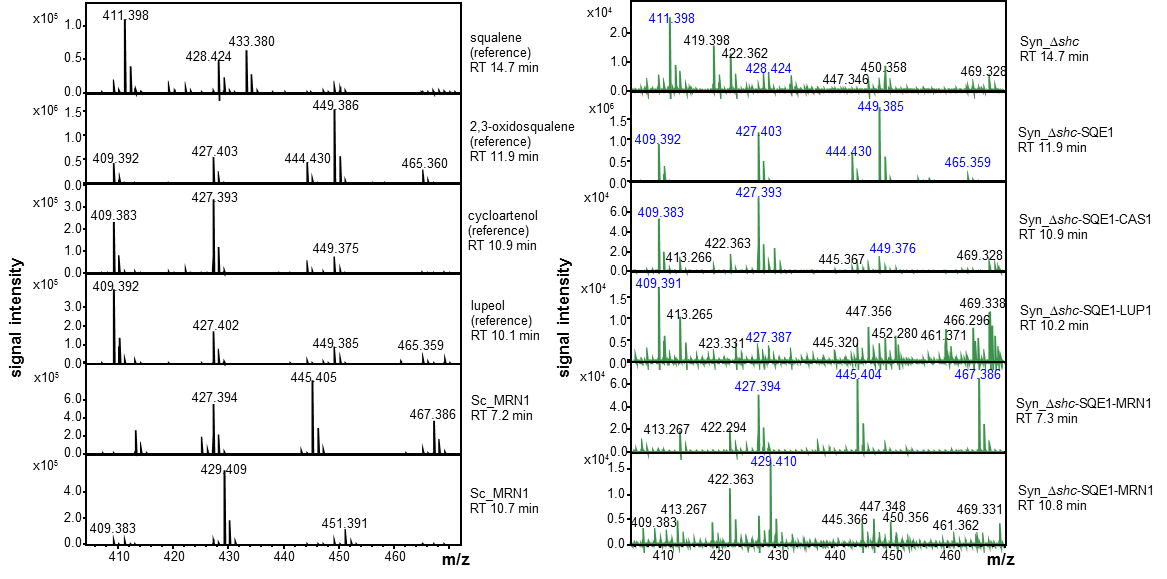


**S2 Fig.A (b): MS spectra of triterpenoids as detected by LC-MS in *Synechocystis* expression strains.** Signals of expected masses obtained with references (left) are highlighted blue in extracts from bacteria (right). Sc_MRN1 extracts were used as a reference for hydroxymarnerol (RT 7.2 min) and marnerol (RT 10.8 min). See **S3 Table** for triterpenoid sum formulas and calculated m/z. Sc, *S. cerevisiae*; Syn, *Synechocystis*.

Figure part (a) for *R. capsulatus* is shown on previous page.

(a)


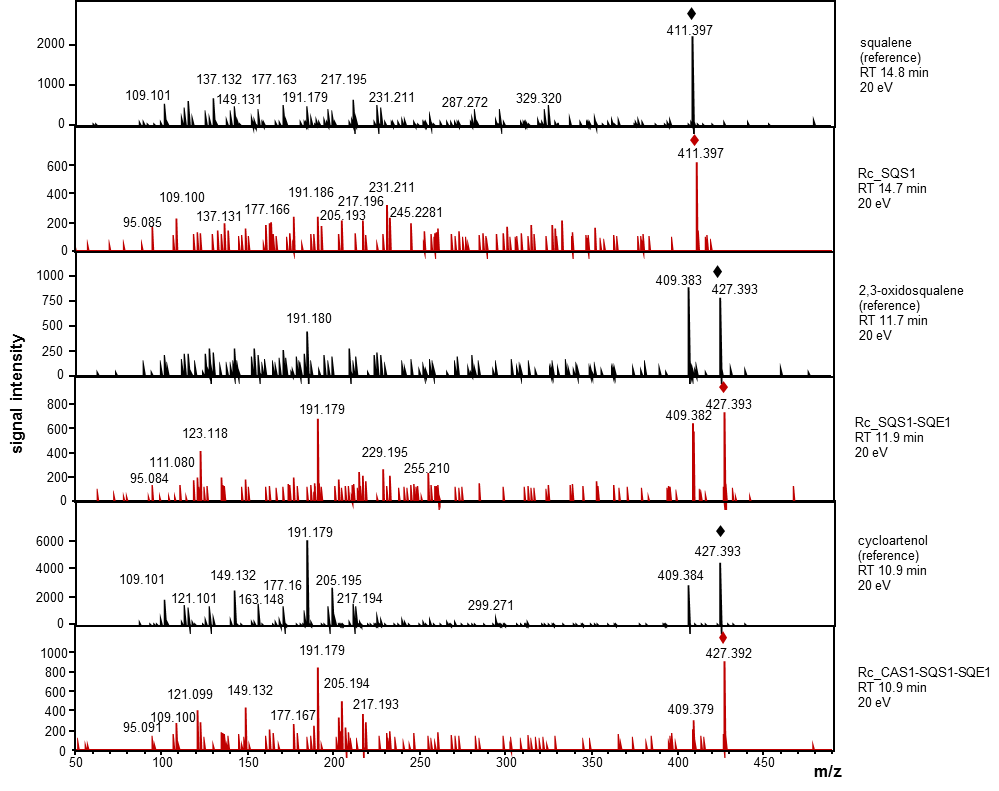


**S2 Fig.B (a): MS/MS spectra of triterpenoids squalene, 2,3-oxidosqualene, and cycloartenol as detected by LC-MS/MS in *R. capsulatus* expression strains.** See **S3 Table** for triterpenoid sum formulas and calculated m/z. The [M+H]^+^ ion (marked with diamond) was selected for fragmentation with 20 eV. Rc, *R. capsulatus*.

Figure part (b) for *Synechocystis* is shown on next page.

(b)


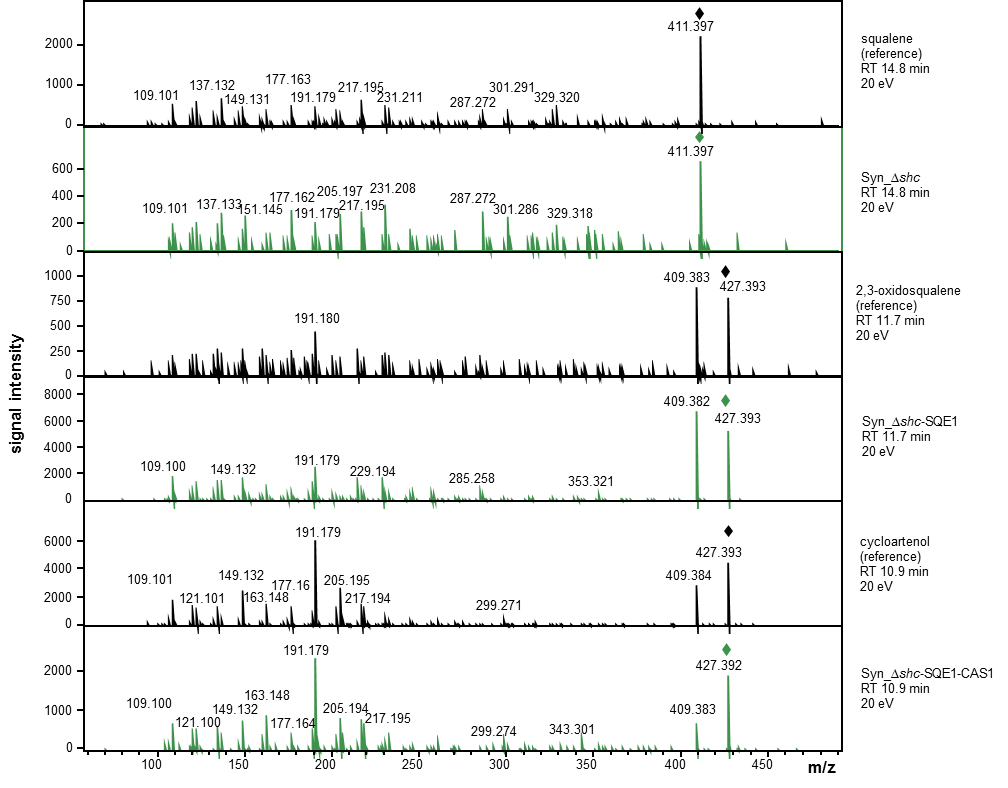


**S2 Fig.B (b): MS/MS spectra of triterpenoids squalene, 2,3-oxidosqualene, and cycloartenol as detected by LC-MS/MS in *Synechocystis* expression strains.** See **S3 Table** for triterpenoid sum formulas and calculated m/z. The [M+H]^+^ ion (marked with diamond) was selected for fragmentation with 20 eV. Syn, *Synechocystis*.

Figure part (a) for *R. capsulatus* is shown on previous page.

(a)


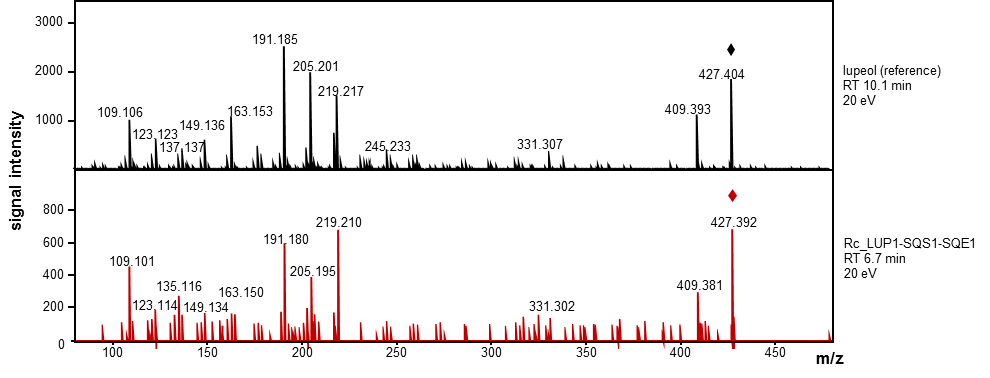


**S2 Fig.C (a): MS/MS spectra of triterpenoids lupX as detected by LC-MS/MS in *R. capsulatus* expression strains and lupeol as reference.** The [M+H]^+^ ion of lupeol (marked with diamond) was selected for fragmentation at 20 eV. M/z 427.404 in lupX presumably corresponds to [M+H-H_2_O]^+^ of a lupeol-related diol (C_30_H_52_O_2_) such as lupan‑3β,20‑diol (Segura *et al*., 2000; DOI: 10.1021/ol006016b). See **S3 Table** for sum formulas and calculated m/z. MS/MS spectra obtained after fragmentation of m/z 427.404 in lupX in Rc_LUP1*-*SQS1-SQE1 were highly similar to lupeol. Rc, *R. capsulatus*.

Figure part (b) for *Synechocystis* is shown on next page.

(b)


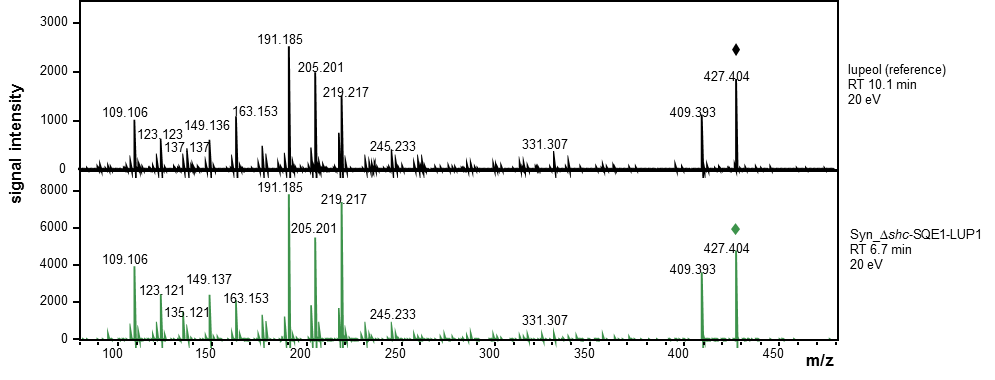


**S2 Fig.C (b): MS/MS spectra of triterpenoids lupX as detected by LC-MS/MS in *Synechocystis* expression strains and lupeol as reference.** The [M+H]^+^ ion of lupeol (marked with diamond) was selected for fragmentation at 20 eV. M/z 427.404 in lupX presumably corresponds to [M+H-H_2_O]^+^ of a lupeol-related diol (C_30_H_52_O_2_) such as lupan‑3β,20‑diol (Segura *et al*., 2000; DOI: 10.1021/ol006016b). See **S3 Table** for sum formulas and calculated m/z. MS/MS spectra obtained after fragmentation of m/z 427.404 in lupX in Syn_*∆shc*-SQE1-LUP1 were highly similar to lupeol. Syn, *Synechocystis*.

Figure part (a) for *R. capsulatus* is shown on previous page.

1. (b)


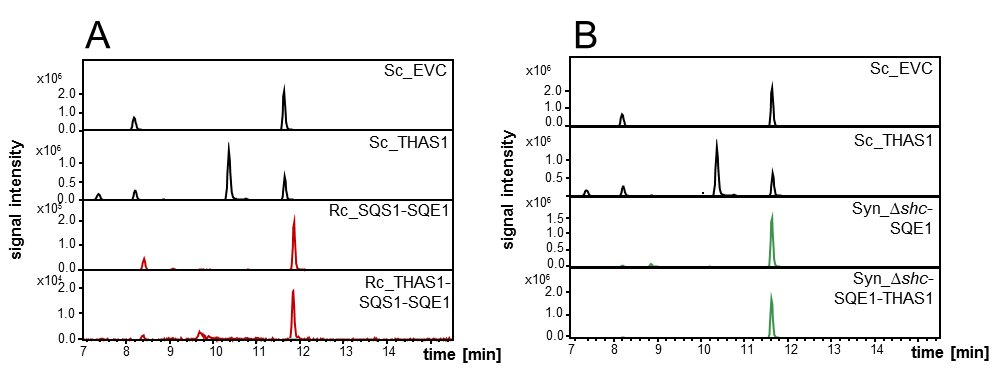


**S2 Fig.D: LC-MS detection of triterpenes produced in *R. capsulatus* strain Rc_THAS1-SQS1-SQE1 (a) and *Synechocystis* strain Syn_Δ*shc*-SQE1-THAS1 (b)*.*** Signal of EIC 427.393 at RT 10.4 min corresponding to thalianol, signal of EIC 427.393 at RT 11.7 min corresponding to 2,3-oxidosqualene. As a reference, chromatograms of samples from *S. cerevisiae*GIL77 (Sc), carrying pYES/DEST-52 with *THAS1* or as empty vector control (EVC) (Field *et al*., 2011; DOI: 10.1073/pnas.1109273108), are shown. No signal corresponding to thalianol was detected in *R. capsulatus* or *Synechocystis*. Sc, *S. cerevisiae*; Rc, *R. capsulatus*; Syn, *Synechocystis*.


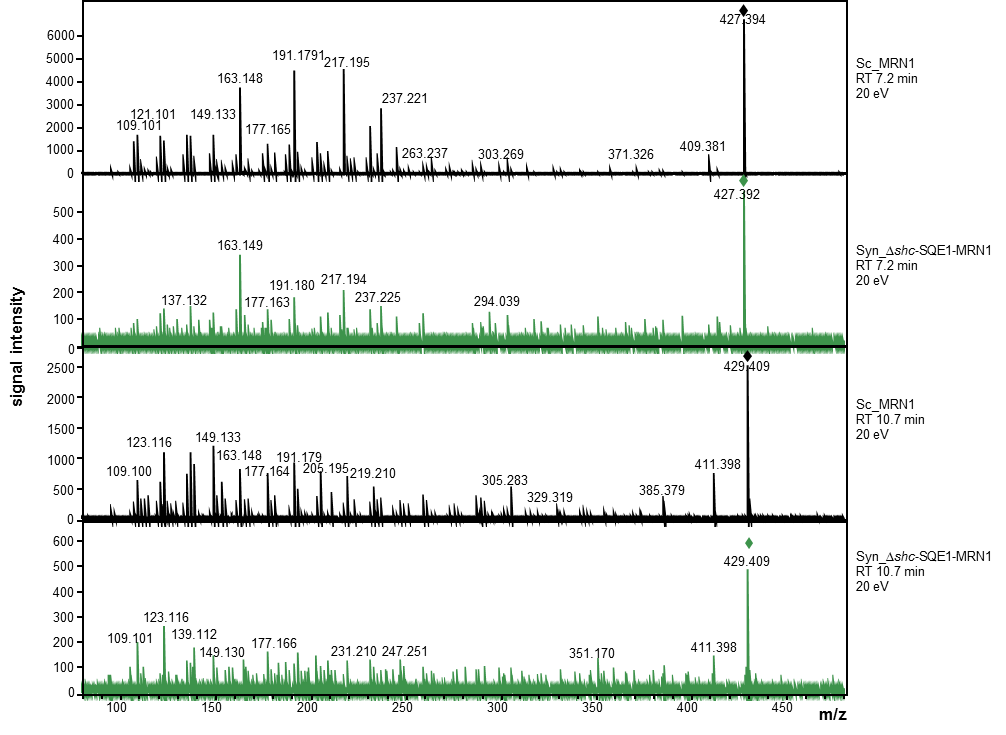


**S2 Fig.E: MS/MS spectra of triterpenoids marnerol and hydroxymarnerol as detected by LC-MS/MS in *Synechocystis* expression strains.** The [M+H]^+^ ion of marnerol (m/z 429.409) at RT 10.7 min was selected for fragmentation at 20 eV (marked with diamond). M/z 427.392 at RT 7.2 min corresponds to [M+H-H_2_O]^+^ of hydroxymarnerol (see **S3 Table** for sum formulas and calculated m/z). Syn, *Synechocystis*; Sc, *S. cerevisiae*.
